# Autotaxin suppresses cytotoxic T cells via LPAR5 to promote anti–PD-1 resistance in non–small cell lung cancer

**DOI:** 10.1172/JCI163128

**Published:** 2023-09-01

**Authors:** Jessica M. Konen, B. Leticia Rodriguez, Haoyi Wu, Jared J. Fradette, Laura Gibson, Lixia Diao, Jing Wang, Stephanie Schmidt, Ignacio I. Wistuba, Jianjun Zhang, Don L. Gibbons

**Affiliations:** 1 Department of Thoracic/Head and Neck Medical Oncology, University of Texas MD Anderson Cancer Center, Houston, Texas, USA.; 2 Department of Hematology and Medical Oncology, Emory University, Atlanta, Georgia, USA.; 3 Department of Surgical Oncology,; 4 Department of Bioinformatics and Computational Biology,; 5 Department of Genomic Medicine,; 6 Department of Translational Molecular Pathology, Division of Pathology/Lab Medicine, and; 7 Department of Molecular and Cellular Oncology, University of Texas MD Anderson Cancer Center, Houston, Texas, USA.

**Keywords:** Immunology, Oncology, Cancer immunotherapy, Lung cancer, Phosphodiesterases

## Abstract

Non–small cell lung cancers that harbor concurrent *KRAS* and *TP53* (KP) mutations are immunologically warm tumors with partial responsiveness to anti–PD-(L)1 blockade; however, most patients observe little or no durable clinical benefit. To identify novel tumor-driven resistance mechanisms, we developed a panel of KP murine lung cancer models with intrinsic resistance to anti–PD-1 and queried differential gene expression between these tumors and anti–PD-1–sensitive tumors. We found that the enzyme autotaxin (ATX), and the metabolite it produces, lysophosphatidic acid (LPA), were significantly upregulated in resistant tumors and that ATX directly modulated antitumor immunity, with its expression negatively correlating with total and effector tumor-infiltrating CD8^+^ T cells. Pharmacological inhibition of ATX, or the downstream receptor LPAR5, in combination with anti–PD-1 was sufficient to restore the antitumor immune response and efficaciously control lung tumor growth in multiple KP tumor models. Additionally, ATX was significantly correlated with inflammatory gene signatures, including a CD8^+^ cytolytic score in multiple lung adenocarcinoma patient data sets, suggesting that an activated tumor-immune microenvironment upregulates ATX and thus provides an opportunity for cotargeting to prevent acquired resistance to anti–PD-1 treatment. These data reveal the ATX/LPA axis as an immunosuppressive pathway that diminishes the immune checkpoint blockade response in lung cancer.

## Introduction

Immune checkpoint blockade (ICB), which targets immunosuppressive axes such as PD-1/PD-L1 via specific antibody treatment, has revolutionized oncological treatment strategies in many cancer types. In particular, non–small cell lung carcinoma (NSCLC) patients have benefited from these inhibitors, including those with late-stage and treatment-refractory disease for which the 5-year survival is only about 8% (1). When treated with PD-1/PD-L1 blocking antibodies, there is an objective response in about 15%–20% of these patients, with some achieving long-term durable responses (2–5). Additional FDA approvals have expanded to include the use of ICB for frontline treatment, alone or in various combinations with chemotherapy or radiotherapy, in the perioperative setting, and/or in a combination of ICB antibodies.

Given the limited number of patients with durable responses to these treatments, research efforts have focused on better understanding those patients who are particularly amenable to ICB, and have discovered that numerous markers, such as tumor mutational burden and PD-L1 status, can be predictors of response in some cases (6–10). Additionally, the tumor genomic landscape can influence inflammatory signatures and treatment response. *KRAS* is one the most prevalent oncogenes in lung adenocarcinoma, mutated in about 25%–30% of patients (11–13). Interestingly, the response of *KRAS* mutant patients to ICB is dependent on the co-occurring mutational profile. Specifically, those with concurrent *KRAS* and *TP53* mutations (termed KP) have an approximately 35% response rate to anti–PD-1 monotherapy, with increased expression of inflammatory signatures and PD-L1 in comparison with other *KRAS*-mutant subtypes (14, 15). These data indicate that KP patients are a logical population to receive ICB, while also underscoring the critical need to identify mechanisms of intrinsic and acquired resistance to generate rational combinatorial treatment strategies to amplify responses.

Using previously described clinically relevant genetically engineered and syngeneic KP mouse models of lung cancer (16), our group has extensively explored ICB response and resistance. Like patients with KP patients, these models initially respond to PD-1/PD-L1 blockade but rapidly acquire resistance. Via overlapping transcriptomic and proteomic data sets from tumors treated with anti–PD-L1 over time in these models, we revealed one mechanism of acquired resistance to be upregulation of the ectoenzyme CD38, which can contribute to the extracellular pools of the suppressive metabolite adenosine (17). These data are in direct alignment with those of others in the field who have discovered that cotargeting the canonical adenosine-generating pathway (CD39/CD73) or the downstream adenosine receptors with anti–PD-1 works efficaciously to promote antitumor immunity, and several clinical trials are currently ongoing for these combination treatments (18–24). Additionally, we previously performed a powerful in vivo shRNA dropout screen using the KP syngeneic models to identify those genes necessary for survival of tumor cells challenged with anti–PD-1 (25). One of these genes was *Ntrk1*, which we found to correlate with an immunologically suppressed microenvironment, likely via upregulation of JAK/STAT signaling cascades and suppressive cytokine expression to promote CD8^+^ T cell dysfunction. Together, these studies underscore the value of the KP syngeneic and autochthonous mouse models of lung cancer in dissecting the immune microenvironment at baseline and with immunotherapy treatment. However, targeting of either CD38 or Ntrk1 was not curative in KP lung tumor models, suggesting that additional mechanisms can drive tumor cell survival in the face of ICB.

To continue to probe tumor-intrinsic mechanisms of ICB resistance, we generated a panel of novel tumor models from KP syngeneic and genetically engineered mouse models (GEMMs) with upfront anti–PD-1 treatment resistance. Analyses of the tumor-immune microenvironment revealed aberrant CD8^+^ T cell signatures in these models that could not be rescued by ICB, specifically a downregulation of total and effector CD8^+^ cells. Using these unique tools and transcriptome data, we probed novel mechanisms driving resistance, revealing *Enpp2*/autotaxin (*Enpp2*/ATX) to be aberrantly upregulated with resistance and a major contributor to CD8^+^ T cell dysfunction in these tumors.

## Results

### Generation of tumor models with intrinsic resistance to anti–PD-(L)1 blockade.

**Figure 1:**
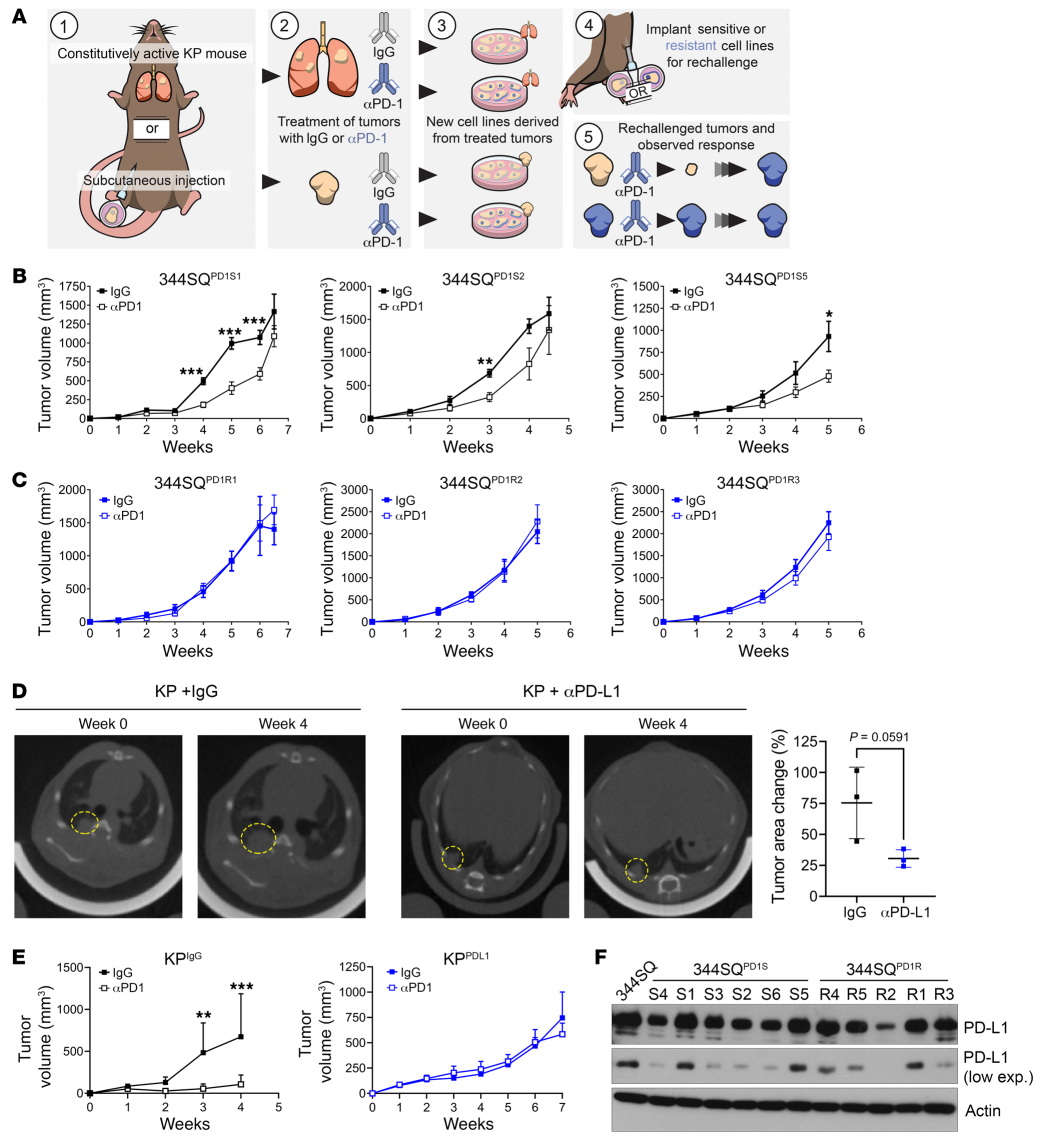
Tumor models created from KP subcutaneous tumors or GEMM lung tumors treated with anti–PD-(L)1 display intrinsic resistance when rechallenged in vivo. (**A**) Schematic illustrating the development of anti–PD-1– or anti–PD-L1–resistant KP tumor models. Tumors were generated either with subcutaneous implantation models using syngeneic 344SQ KP murine lung cancer cells or from autochthonous lung tumors developed in the Kras^LA1-G12D^/p53^R172HΔg^ GEMM. Mice were then treated with IgG control or PD-1/PD-L1 axis–blocking antibodies until the development of resistance. At this point, tumors were excised, cultured, and expanded ex vivo, and then reimplanted into wild-type (WT) mice for rechallenge with anti–PD-(L1). (**B**) Three of the 344SQ IgG-treated tumors described in **A** (344SQ^PD1S^) were implanted into WT mice and treated with either IgG or anti–PD-1. Tumors were measured weekly with calipers. *n* = 5 mice per group. *P < 0.05, **P < 0.01, ***P < 0.001 by multiple *t* tests (1 per time point). (**C**) The anti–PD-1–treated tumors described in **A** (344SQ^PD1R^) were implanted and treated as in **B**. (**D**) Kras^LA1-G12D^/p53^R172HΔg^ mice were imaged by micro-CT to confirm lung nodule formation. Mice were randomly distributed into IgG or anti–PD-L1 treatment arms and treated for 4 weeks. Endpoint images using micro-CT were taken (left). The percentage change in tumor area was measured for 3 independent tumors per mouse (right). (**E**) Cell lines were derived from the IgG-treated (KP^IgG^) or anti–PD-L1–treated (KP^PDL1^) GEMMs described in **D** and implanted into WT mice. Mice were rechallenged with anti–PD-L1 or IgG control antibodies and tumor response measured over time using calipers. *n* = 5 mice per group. **P < 0.01, ***P < 0.001 by multiple *t* tests (1 per time point). (**F**) 344SQ^PD1S^ and 344SQ^PD1R^ cells were analyzed for PD-L1 expression by Western blotting (see supplemental material for full, uncut gels). Actin was used as a loading control.

To examine PD-1 blockade resistance, we created new lung tumor models via in vivo challenge of previously described PD-(L)1–sensitive KP syngeneic and autochthonous models (17) (Figure 1A). Tumors were treated with IgG control or anti–PD-(L)1 until resistance developed, at which point lung or subcutaneous tumors were excised, processed into single cells, and grown in vitro over several passages to remove all stroma and non-malignant cell types from the cultures. For the 344SQ subcutaneous model, we confirmed that defining cellular characteristics of the newly derived models matched those of the parental line, such as cellular morphology and features of the epithelial-mesenchymal transition (Supplemental Figure 1, A–C; supplemental material available online with this article; https://doi.org/10.1172/JCI163128DS1). After ex vivo passaging, cells were implanted into wild-type mice and rechallenged with ICB. Those tumors that were previously treated and acquired resistance to anti–PD-1 demonstrated upfront resistance when rechallenged in vivo (termed 344SQ^PD1R^), whereas those that were treated with the IgG antibody demonstrated initial response but eventual resistance to ICB (termed 344SQ^PD1S^) (Figure 1, B and C). We also tested the effects of targeting the axis via anti–PD-L1 and found similar results, with no response in either primary tumor growth or metastasis in the 344SQ^PD1R^ models (Supplemental Figure 1, D and E). Similarly, we found that the KP autochthonous model of lung cancer demonstrated a partial response to single-agent anti–PD-L1 as measured by lung CT scans taken after 4 weeks of treatment (Figure 1D); however, the model generated from anti–PD-L1–treated lung tumors (termed KP^PDL1^) demonstrated upfront resistance when rechallenged subcutaneously in vivo as compared with IgG-treated lung tumors (KP^IgG^) (Figure 1E).

One documented mechanism of resistance to ICB is altered expression of the target axis within the tumor. Therefore, we examined the expression of PD-L1 in the 344SQ^PD1R^ cells and tumors compared with the sensitive models. By Western blotting and flow cytometry, we found heterogeneous expression of PD-L1 across all models (Figure 1F and Supplemental Figure 2A), providing evidence that downregulation of this axis is not correlated with response. Additionally, other pathways known to be vital in creating an immune response were also intact in the 344SQ^PD1R^ panel, including IFN-γ response, JAK/STAT signaling cascades, and expression of antigen presentation machinery including Tap1/2 and MHCI (Supplemental Figure 2, B–E). Lastly, our group revealed that tumor-expressing CD38 promotes anti–PD-1 resistance in KP lung cancer (17); therefore, we also examined the expression of CD38 in the 344SQ^PD1R^ models. Interestingly, we did not find evidence that the resistant models upregulate CD38 (Supplemental Figure 2, F and G), suggesting that CD38 is transiently upregulated in tumors but does not persist during ex vivo expansion. These data indicate that the anti–PD-1–resistant models maintain known immune-activating pathways, but do not respond to ICB, suggesting that these tumor models utilize novel mechanism(s) to promote survival.

### Anti–PD-1–resistant tumor models display suppressed CD8^+^ T cell populations in treatment-naive and anti–PD-1 rechallenge settings.

**Figure 2:**
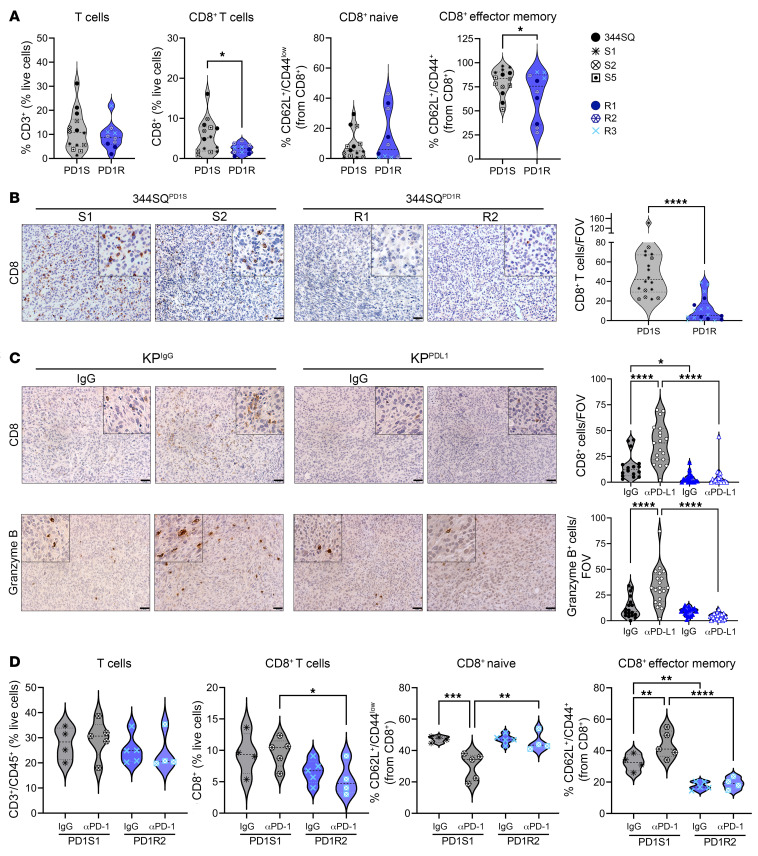
Anti–PD-1–resistant tumor models demonstrate reduced CD8^+^ T cell and effector functions compared with sensitive tumors. (**A**) Three of the 344SQ^PD1R^ lines, 3 of the 344SQ^PD1S^ lines, and the 344SQ parental line were implanted into WT mice. After 3 weeks, tumors were excised, processed into single cells, and stained for multicolor flow cytometry analysis of immune cell subsets. The total intratumoral T cells were gated as CD3^+^ as a percentage of total CD45^+^ cells. Under total T cells, we then analyzed the CD8^+^ T cells for total amounts and effector memory (CD62L^–^CD44^+^) or naive (CD62L^+^CD44^–^) status. Individual models are denoted by different symbols and colors. *n* = 2–5 mice per model. *P < 0.05 by *t* test. (**B**) Two representative cell lines for both 344SQ^PD1S^ and 344SQ^PD1R^ were implanted into WT mice and tumors grown until endpoint (about 6–7 weeks). Tumors were collected and analyzed via IHC for CD8^+^ T cells. A representative image per model is depicted (left). All tumors per group were combined and graphed as total CD8^+^ T cells per field of view (FOV) (right). *n* = 2 mice per cell line, 3–6 images per mouse tumor. ****P < 0.0001 by *t* test. Scale bars: 50 μm; insets zoomed 200%. (**C**) The KP^IgG^ and KP^PDL1^ tumors from Figure 1E were collected for IHC and analyzed for total CD8^+^ T cells (top) and granzyme B staining (bottom). *n* = 3 tumors per condition, 5–6 images per tumor. *P < 0.05, ****P < 0.0001 by 1-way ANOVA with multiple comparisons corrected. Scale bars: 50 μm; insets zoomed 200%. (**D**) 344SQ^PD1S1^ and 344SQ^PD1R2^ models were implanted into WT mice and then treated with either IgG control or anti–PD-1 antibody. After 2 weeks of treatment, tumors were excised and analyzed via multicolor flow as described in **A**. *P < 0.05, **P < 0.01, ***P < 0.001, ****P < 0.0001.

To determine whether the survival of the ICB-resistant models is correlated with alterations in the immune microenvironment, we analyzed tumor-infiltrating immune populations within anti–PD-1–resistant models at baseline and in the face of ICB. The 344SQ parental line, three 344SQ^PD1S^, and three 344SQ^PD1R^ models were implanted into mice. After 3 weeks, tumors were processed for multicolor flow cytometry to analyze immune subpopulations and their functional status (gating schema depicted in Supplemental Figure 3A). Compared with the sensitive models, 344SQ^PD1R^ tumors had significantly fewer CD8^+^ and CD4^+^ T cells (Figure 2A and Supplemental Figure 3B). Additionally, those CD8^+^ T cells present were less in the effector memory state as measured by CD62L and CD44 (Figure 2A). We confirmed that these results persist into later-stage tumors by assaying endpoint tumors (weeks 6–7) by IHC analysis and found that the resistant tumors had approximately 5 times fewer CD8^+^ T cells compared with the sensitive tumors (Figure 2B). We also used the KP^IgG^ and KP^PDL1^ GEMM-derived models, confirming a reduction in CD8^+^ T cells in KP^PDL1^ tumors with or without treatment (Figure 2C). Our flow analysis included cells within the myeloid compartment, as these immune subsets can be essential for T cell activation. We found a trend toward a reduction in dendritic cells, a significant reduction in M1-like macrophages, and a significant increase in M2-like macrophages in the anti–PD-1–resistant models (Supplemental Figure 3C).

To understand the effects of PD-1 blockade on immune populations in these tumors, we analyzed one representative sensitive and resistant model with anti–PD-1 treatment by flow cytometry. Our data confirmed the findings above that the 344SQ^PD1R2^ model had fewer CD8^+^ T cells and the effector status of these T cells was reduced (Figure 2D). PD-1 blockade had little beneficial impact on the CD4^+^ compartment (Supplemental Figure 3D); however, it significantly increased the CD8^+^ effectors in the 344SQ^PD1S1^ tumors, but these cells remained unaffected in anti–PD-1–treated 344SQ^PD1R2^ tumors (Figure 2D). While we found significantly reduced M1-like macrophages with resistance in baseline tumors, we did not observe any changes to this population with anti–PD-1 treatment (Supplemental Figure 3E); therefore, we only focused on the CD8^+^ T cell populations moving forward, though more studies are required to explore the macrophage compartment. Together, these data suggest that the 344SQ^PD1R^ tumors have diminished total and effector CD8^+^ T cells, even when challenged with anti–PD-1.

### Enpp2/ATX and its bioactive metabolite, LPA, are upregulated in resistant models.

**Figure 3:**
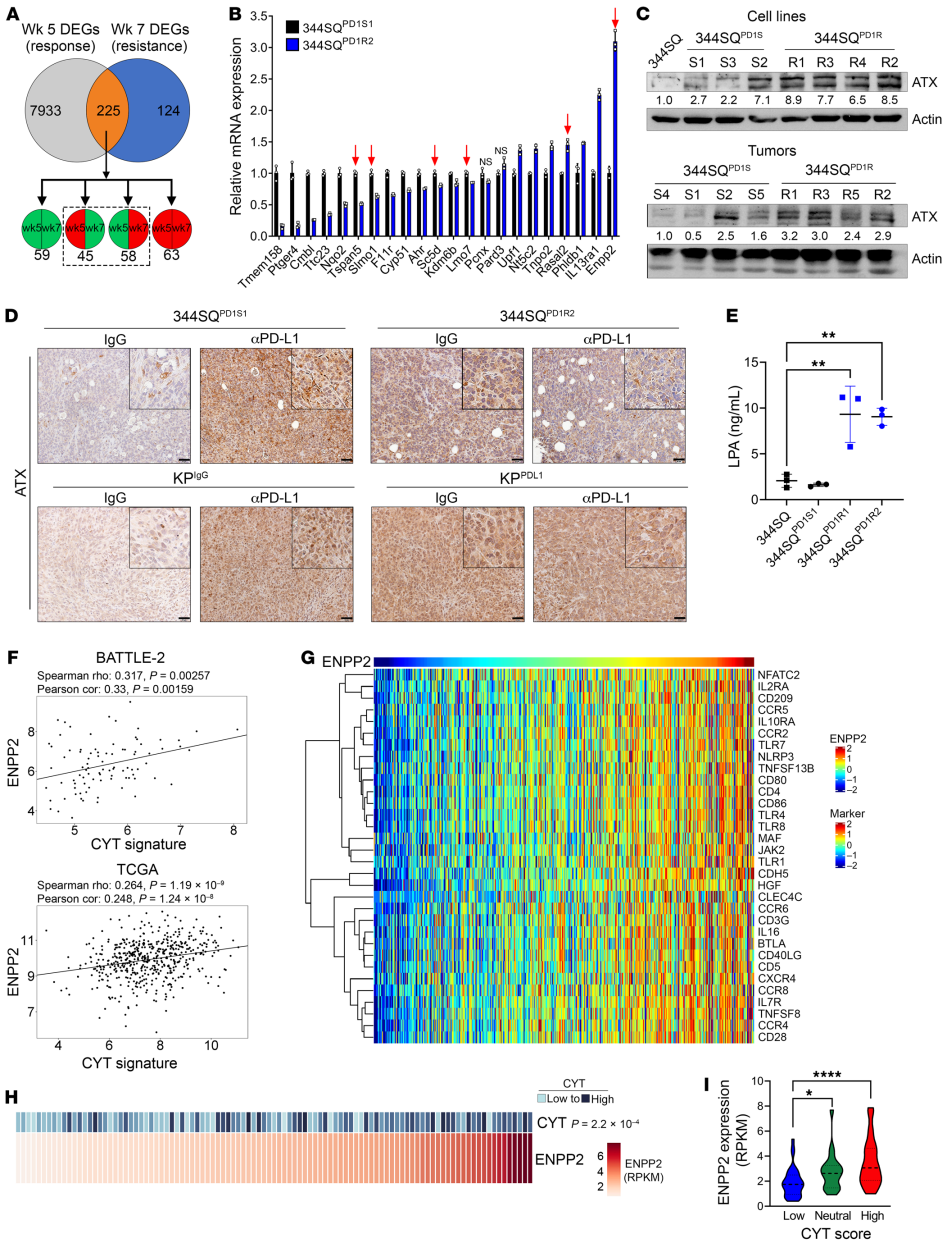
*Enpp2*/ATX is upregulated with PD-(L)1 resistance in KP murine models and cytolytic gene signature in patients with human lung adenocarcinoma. (**A**) Previously published transcriptomics from IgG or anti–PD-L1–treated 344SQ tumors were analyzed at week 5 (response) and week 7 (resistance) (17). DEGs between treatments at each time point (225 total) were analyzed for directionality, and we focused on DEGs that changed in directionality between time points (dashed box). (**B**) The top DEGs from **A** were analyzed via quantitative PCR in 344SQ^PD1S1^ and 344SQ^PD1R2^ cells and are graphed relative to 344SQ^PD1S1^. Arrows denote genes changing in the same direction as the microarray. All genes except those marked “NS” are significantly different at *P* < 0.05, by *t* test. (**C**) The 344SQ^PD1S^ and 344SQ^PD1R^ cells (top) and tumors (bottom) were analyzed via Western blotting for *Enpp2*/ATX expression. Actin densitometric values were normalized to the corresponding actin band and then to the first lane. (**D**) Representative ATX IHC images in anti–PD-L1– or IgG-treated 344SQ^PD1S1^ and 344SQ^PD1R2^ (top) or KP^IgG^ and KP^PDL1^ (bottom) tumors. Scale bars: 50 μm; insets zoomed 200%. (**E**) Conditioned media from 344SQ^PD1S^ and 344SQ^PD1R^ models were analyzed for LPA via ELISA. **P < 0.01, by 1-way ANOVA. (**F**) *ENPP2* expression in lung adenocarcinoma patients with lung adenocarcinoma was correlated with a previously described T cell cytolytic score (CYT) (62) in BATTLE-2 (top) and TCGA Firehouse Legacy (bottom) data sets. (**G**) *ENPP2* expression in TCGA Firehouse Legacy samples was correlated with a previously published inflammatory gene signature (33) (rho cutoff, 0.4; FDR, 0.05). (**H** and **I**) Analysis of *ENPP2* in the MD Anderson ICON data set. (**H**) Correlation of *ENPP2* with the CYT score as described in **F**. (**I**) *ENPP2* expression was compared across ICON patients grouped as having a low, neutral, or high CYT score. *P < 0.05 and ****P < 0.0001, by Wilcoxon’s rank-sum testing.

To identify tumor cell mechanisms involved in driving the immune phenotype described above, we used previously published transcriptomic data of anti–PD-L1–treated 344SQ tumors, analyzed during response (week 5) and at the development of resistance (week 7) (17). Comparing anti–PD-L1– with IgG-treated tumors, we found 8,158 significantly differentially expressed genes (DEGs) at week 5 and 349 DEGs at week 7. Of these, 225 genes overlapped between the 2 time points (Figure 3A and Supplemental Table 1). We then compared the directionality of expression of these 225 DEGs and focused only on those that changed over time (e.g., went from downregulated to upregulated), as these genes may be specifically associated with resistance. We analyzed the mRNA expression of the top 22 DEGs in a representative pair of 344SQ sensitive and resistant models and found that only 2, *Rasal2* and *Enpp2*, were significantly different between the models with the same directionality as the transcriptome data (Figure 3B). However, only *Enpp2* was found to be consistently upregulated across the broader panel of the anti–PD-1–resistant models; thus, we focused on this potential candidate moving forward.

*Enpp2* encodes the protein ATX, a secreted phosphodiesterase with enzymatic function to convert lysophosphatidylcholine (LPC) to lysophosphatidic acid (LPA) (26, 27). We confirmed that ATX was upregulated at the protein level via Western blotting of 344SQ^PD1S^ and 344SQ^PD1R^ cells or tumors (Figure 3C). The GEMM-derived models demonstrated a similar trend in ATX expression (Supplemental Figure 4A). Lastly, we performed IHC staining for ATX expression within tumors challenged with anti–PD-L1 in the syngeneic 344SQ and the autochthonous KP models. In the sensitive models, long-term treatment with anti–PD-L1 caused an upregulation of ATX, again confirming that the development of acquired resistance correlates with higher ATX expression (Figure 3D). Similarly, ATX was ubiquitously expressed at high levels across tumor sections in the models with intrinsic resistance to anti–PD-(L)1 (Figure 3D).

ATX is synthesized as a pre-proenzyme, becoming activated and secreted with glycosylation and proteolytic cleavage (28); therefore, its enzymatic activity occurs largely within the extracellular space. Therefore, we examined secreted ATX levels. Using conditioned media (CM) collected from anti–PD-1 sensitive and resistant models, we found that secreted ATX was also significantly higher in 344SQ^PD1R^ CM compared with 344SQ^PD1S^ CM samples (Supplemental Figure 4B). Interestingly, the phospholipid phosphatase enzymes (*Plpp1–Plpp3*), which are important for rapid degradation of LPA into monoacylglycerol, were also downregulated in the 344SQ^PD1R^ tumors (Supplemental Figure 4C), suggesting that more LPA is generated and is not as efficiently metabolized in an anti–PD-1–resistant setting. We confirmed this by using an ELISA measuring total LPA concentration in CM from sensitive and resistant lines, finding approximately 5 times the amount of LPA secreted from the resistant models (Figure 3E). Taken together, these data demonstrate that the ATX/LPA axis is aberrantly upregulated in anti–PD-1 models.

To understand how the observed expression in the experimental models relates to patient samples, we analyzed *ENPP2* gene expression as a function of inflammatory gene signatures in 3 independent NSCLC data sets: the BATTLE-2 trial of metastatic NSCLC (29) and the Immunogenomic Profiling of Non–Small Cell Lung Cancer Project (ICON) (30) and The Cancer Genome Atlas (TCGA) data sets of early-stage surgically resected tumors (Figure 3, F–I). We found that *ENPP2* expression positively correlated with a cytolytic gene signature (*GRZA*, *PRF1*, *CD8A*) in all 3 data sets (Figure 3, F, H, and I), suggesting that ATX may be induced during an activated immune response in ICB-naive tumors. These data support evidence from previous studies demonstrating that inflammatory cytokines like IL-1β and TNF-α can promote ATX transcription (31, 32). Other features, like tumor mutational burden and tumor stage, did correlate with *ENPP2* (Supplemental Figure 4D). Additionally, we used a previously published inflammatory gene signature that includes markers for immunosuppressive cell types, immune checkpoint molecules, and cytokines (17, 33) and found a significant correlation between many of these markers and *ENPP2* (Figure 3G), including immune checkpoints PD-1, PD-L1, TIM-3, and BTLA (Table 1).

Because data sets with RNA profiling of large numbers of patients for ICB-treated NSCLC are not readily available, we used a published melanoma data set taken from paired pretreatment and on-treatment biopsy samples, where patients received nivolumab and response data are available (34), to correlate *ENPP2* expression with immunotherapy treatment response. We analyzed *ENPP2* expression in these samples and found that a portion of patients demonstrated an increase in *ENPP2* expression while on treatment, while others showed no change or a decrease (Supplemental Figure 4E). To determine the impact of changing *ENPP2* expression on treatment response, we correlated the delta in expression between pretreatment and on-treatment samples with the clinical response to treatment. Those patients with progressive or stable disease tended to have a higher delta in *ENPP2* (increased on treatment) compared with those patients with a partial or complete response (Supplemental Figure 4F). Overall, these clinical data from NSCLC and melanoma, while limited by lack of ICB treatment and tumor type, respectively, support our preclinical studies demonstrating an increase in ATX after an initial response and subsequent resistance to anti–PD-1 treatment.

### ATX expression negatively correlates with CD8^+^ T cell infiltration and effector status.

**Figure 4:**
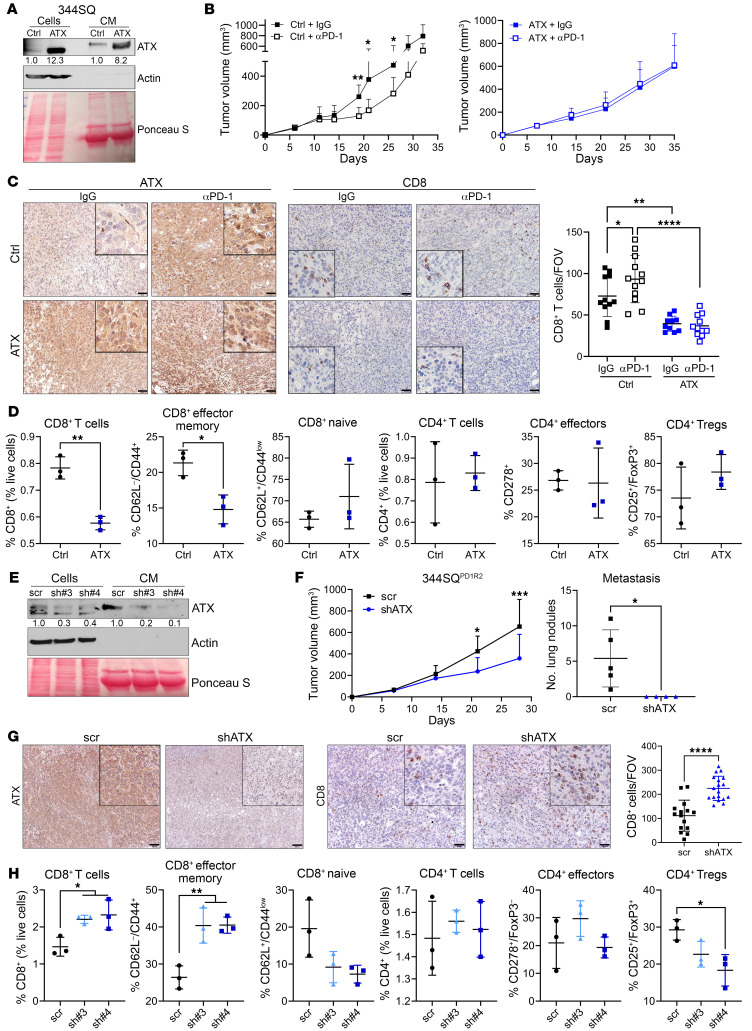
ATX expression negatively correlates with CD8^+^ T cell infiltration and effector status in tumors. (**A**) 344SQ-control (ctrl) or ATX-overexpressing cells were analyzed via Western blotting of cells and conditioned media (CM). ATX densitometric values were normalized to the corresponding actin or Ponceau bands and then to 344SQ-ctrl. (**B**) Tumor growth was measured from mice implanted with 344SQ-ctrl or -ATX cells and treated with IgG or anti–PD-1. *n* = 5 mice per group. *P < 0.05 and **P < 0.01, by multiple *t* tests (per time point). (**C**) Representative ATX and CD8 IHC images completed on tumors from **B**. CD8^+^ T cells were quantified as number per FOV. *n* = 3 mice each. *P < 0.05, **P < 0.01, and ****P < 0.0001, by 1-way ANOVA. Scale bars: 50 μm; insets zoomed 300% (ATX) or 250% (CD8). (**D**) 344SQ-ctrl or -ATX cells were cocultured with naive immune cells over time, and immune cell populations were analyzed by flow cytometry. The experiment was completed twice. *P < 0.05 and **P < 0.01, by *t* test. (**E**) 344SQ^PD1R2^ cells depleted of ATX using 2 shRNAs or a control (scr) were analyzed as in **A**. (**F**) Tumor growth from 344SQ^PD1R2^-scr and shATX#4 cells implanted into mice was monitored via calipers (left). Metastatic lung nodules were quantified at necropsy (right). *n* = 4–5 mice per group. *P < 0.05 and ***P < 0.001, by multiple *t* tests (**G**) Representative ATX and CD8 IHC images completed on tumors from **F**. *n* = 2 mice each, 6–9 FOV per tumor. ****P < 0.0001, by *t* test. Scale bars: 100 μm (ATX), 50 μm (CD8); insets zoomed 200%. (**H**) The 344SQ^PD1R2^-scr and shATX cells from **E** were cocultured with naive immune cells as in **D**. The experiment was completed twice. *P < 0.05, **P < 0.01, and ***P < 0.001, by 1-way ANOVA.

We next sought to understand whether forced expression of ATX is sufficient to create anti–PD-1 treatment resistance. To address this, we created a constitutively overexpressed ATX in the 344SQ cells (Figure 4A and Supplemental Figure 5A). ATX expression does not contribute to tumor cell growth in vitro (even with addition of exogenous LPC substrate); however, ATX promoted a more invasive phenotype in Transwell assays and 3D cultures (Supplemental Figure 5, B–D). To determine the interaction between ATX expression and the immune microenvironment, we next tested the impact of ATX overexpression on anti–PD-1 response. Like the 344SQ parental tumors, the vector control tumors demonstrated a partial response to anti–PD-1 (Figure 4B). However, ATX overexpression was sufficient to generate upfront treatment resistance (Figure 4B), providing evidence that this axis can directly impact tumor response to PD-1 blockade. To determine the effects of ATX overexpression on the infiltrating immune populations, we performed IHC for CD8^+^ T cells and also confirmed ATX overexpression (Figure 4C). The CD8^+^ T cell infiltration mirrored the results seen in the 344SQ^PD1R^ and KP^PDL1^ tumor models, with reduced CD8^+^ T cell infiltration with ATX overexpression in IgG-treated tumors and a marked reduction in the face of anti–PD-1 (Figure 4C). We corroborated these data with flow cytometry analyses of immune cell populations and found that constitutive overexpression of ATX negatively correlated with an activated CD8^+^ T cell phenotype, but no alterations to CD4^+^ subpopulations (Figure 4D and Supplemental Figure 5, E and F).

To connect ATX expression in an intrinsic anti–PD-1–resistant setting with tumor survival and immune functionality, we stably depleted ATX with targeted shRNAs in one of the representative 344SQ^PD1R^ models (Figure 4E). Interestingly, ATX knockdown was sufficient to reduce primary tumor growth in vivo in comparison with the control tumors, as well as completely abolish the metastatic propensity of these tumors (Figure 4F). We confirmed that ATX expression was reduced in tumors by IHC analysis and found an inverse correlation between ATX levels and the infiltration of CD8^+^ T cells (Figure 4G). Lastly, we demonstrated via flow cytometry analysis that ATX expression altered CD8^+^ but not CD4^+^ T cell populations, with knockdown significantly increasing CD8^+^ effectors (Figure 4H and Supplemental Figure 5, G and H). Taken together, these data provide evidence that ATX expression can alter intratumoral immune cell functionality and the antitumor efficacy of PD-1 blockade.

### Cotargeting ATX with anti–PD-1 promotes antitumor CD8^+^ T cell activity.

**Figure 5:**
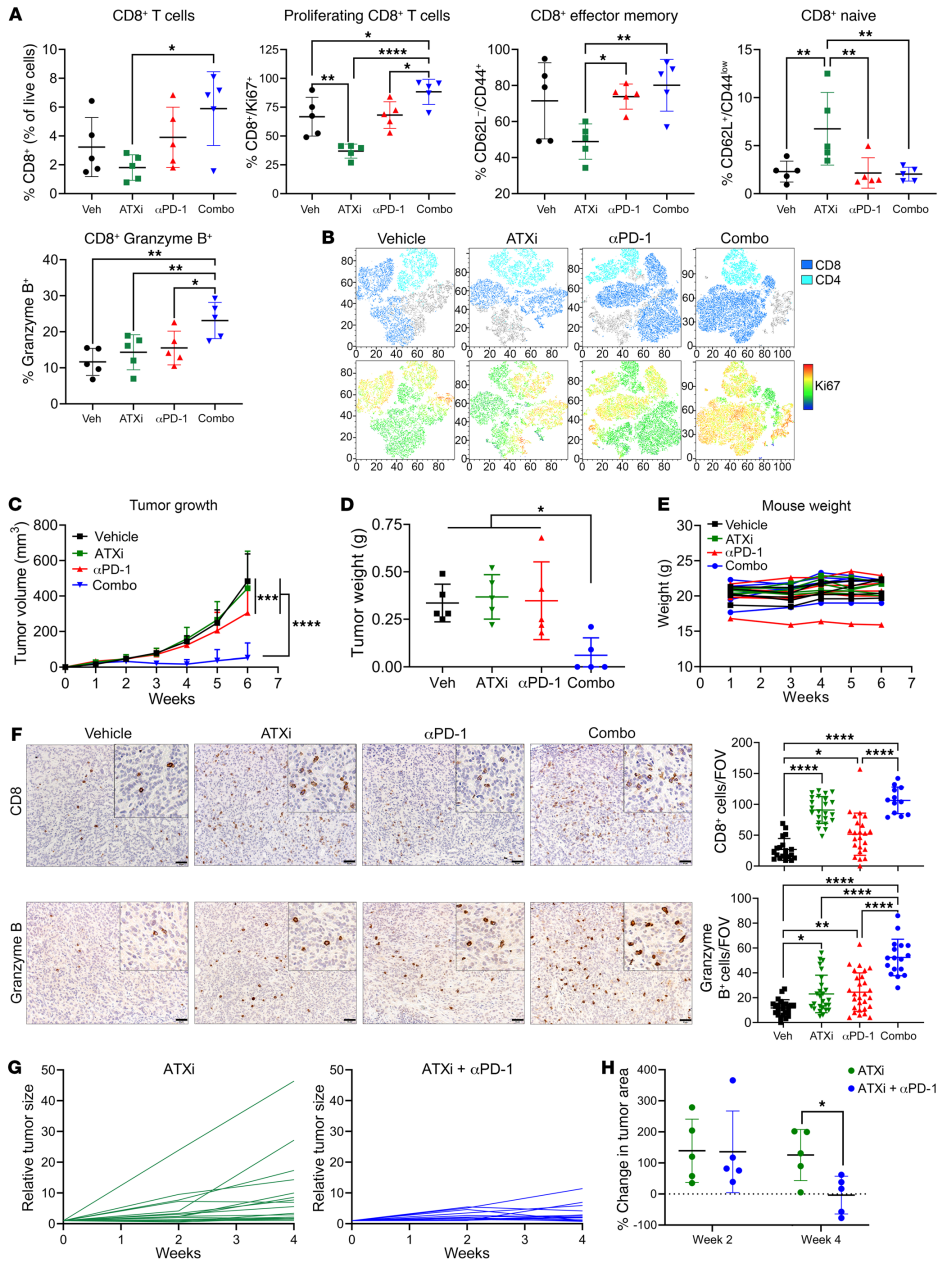
Pharmacological targeting of ATX in combination with PD-1 blockade promotes CD8^+^ T cell proliferation and activation, effectively controlling tumor growth in vivo. (**A**) 344SQ cells were implanted into mice and treated with IgG/vehicle, ATX inhibitor (ATXi), anti–PD-1, or a combination. After 1 week of treatment, tumors were processed for flow cytometry of immune populations. *P < 0.05, **P < 0.01, and ****P < 0.0001, by 1-way ANOVA. (**B**) Representative *t*-distributed stochastic neighbor embedding (TSNE) plots of data from **A**. Total CD3^+^ (top) and CD3^+^Ki67^+^ (bottom) cells are depicted. (**C**–**F**) 344SQ cells were implanted into mice and treated as described in **A**. *n* = 5 mice per group. (**C**) Tumor growth was measured via calipers. ***P < 0.001 and ****P < 0.0001, by 2-way ANOVA with Tukey’s correction. (**D**) Tumor weights were collected at necropsy. *P < 0.05, by 1-way ANOVA with Tukey’s correction. (**E**) Mouse weights were recorded weekly. (**F**) Representative CD8 (top) and granzyme B (bottom) IHC images on tumors from **C**. Cells per FOV were quantified as in Figure 2D. *n* = 3 mice per group (except the combination, which had 2 tumors at endpoint), 6–9 FOV per tumor. *P < 0.05, **P < 0.01, and ****P < 0.0001, by 1-way ANOVA with Tukey’s correction. Scale bars: 50 μm; insets zoomed 200%. (**G** and **H**) Kras^LSL-G12D^/p53^wmR172H^ mice were given adenoviral Cre recombinase intratracheally, and tumor formation was monitored via micro-CT imaging (Supplemental Figure 7D). After tumor development, mice were randomized and treated for 4 weeks. *n* = 5 mice per group. (**G**) Individual tumors were measured at weeks 0, 2, and 4 and normalized to week 0. (**H**) Percentage change of tumor size was calculated between each time point. All individual tumors per mouse were measured, and median growth is shown. *n* = 5 mice per group. *P < 0.05, by *t* test.

Our data suggest that ATX/LPA levels correlate with resistance to anti–PD-1, which has translational relevance as ATX/LPA inhibitors are being investigated for treatment of pulmonary fibrosis. To analyze the effects on immune functionality and provide preclinical evidence for this treatment strategy, we tested the efficacy of the ATX inhibitor PF-8380, which we confirmed inhibits LPA accumulation (Supplemental Figure 6, A and B), alone and in combination with anti–PD-1. 344SQ tumors were implanted and, after 1 week, were treated with each treatment arm for an additional week (Supplemental Figure 6C). Tumors were processed for flow cytometry to analyze immune subpopulations. We found an increase in both CD4^+^ and CD8^+^ T cell populations, accompanied by a robust increase in Ki67^+^ proliferating cells in each subset, with the combination treatment (Figure 5, A and B, and Supplemental Figure 6D). Anti–PD-1 alone increased the CD8^+^ effector memory status as seen previously, while combination treatment increased this population to an even greater extent. Lastly, the combination tumors had significantly increased granzyme B^+^ CD8^+^ T cells compared with all other arms (Figure 5A). Together, these data indicate that the combination of ATX inhibition with anti–PD-1 robustly improved tumor-infiltrating CD8^+^ T cell functionality.

### ATX inhibition combined with anti–PD-1 significantly controls KP tumor growth.

To determine whether these immune changes correlated with reduced tumor growth, we tested the long-term efficacy of ATX inhibitor with PD-1 blockade as described above. After 5 weeks of treatment, the combination was significantly more efficacious than either single agent at controlling tumor growth, and 60% of mice treated with the combination demonstrated complete tumor regressions (Figure 5, C and D), with no significant changes in mouse body weight observed (Figure 5E), suggesting it is well tolerated. We also tested ATX inhibition with anti–PD-L1 in the KP^IgG^ model and found that after 4 weeks of treatment, the combination treatment arm had significantly smaller tumors compared with the vehicle-treated tumors (Supplemental Figure 6E), whereas in the KP^PDL1^ resistant model, ATX inhibitor alone was sufficient to significantly repress tumor growth (Supplemental Figure 6F).

We also analyzed CD8^+^ T cells in the late-stage tumors by IHC and found that single-agent ATX inhibitor and the anti–PD-1/ATX inhibitor combination significantly increased CD8^+^ T cells, from about 20 CD8^+^ T cells per field of view (FOV) in the vehicle treatment to about 90 and 100 CD8^+^ T cells per FOV in the ATX inhibitor and combination tumors, respectively (Figure 5F). However, while single agents caused a minor increase in the granzyme B^+^ cells in tumors, only the combination demonstrated a robust increase. These data indicate that early changes in immune populations persist throughout a longer treatment regimen, leading to significantly improved CD8^+^ T cell functionality that controls tumor growth and, in some cases, promotes tumor rejection.

Lastly, we tested the efficacy of this combination in a clinically relevant GEMM of lung cancer. For this, we used the conditional Kras^LSL-G12D^/p53^wm-R172H^ GEMM, specifically activating the Kras/p53 mutations in the lung via intratracheal delivery of adenoviral Cre recombinase as previously described (35, 36) (Supplemental Figure 7, A–C). These mice develop primary lung adenocarcinomas approximately 8–12 weeks after infection, which is monitored by CT imaging. Once lung tumors were observed, mice were randomly enrolled into either ATX inhibitor or ATX inhibitor plus anti–PD-1 treatments. Mice were treated for 4 weeks and were imaged before treatment (week 0), on treatment (week 2), and at endpoint (week 4) (Supplemental Figure 7D). Comparing week 0 to week 4 CT scans, the mice treated with the combination had significantly smaller tumors (Figure 5, G and H, and Supplemental Figure 7E). In fact, 2 of 5 mice treated with the ATX inhibitor/anti–PD-1 combination demonstrated net tumor regressions between weeks 2 and 4 (Figure 5H). Taken together, these data demonstrate the efficacy of combining an ATX inhibitor with anti–PD-1 to significantly control and shrink KP mutant tumors, with associated increases in the CD8^+^ T cell proliferation, activation, and cytotoxic function.

### CD8^+^ T cells express multiple LPARs that can respond to LPA stimulation.

**Figure 6:**
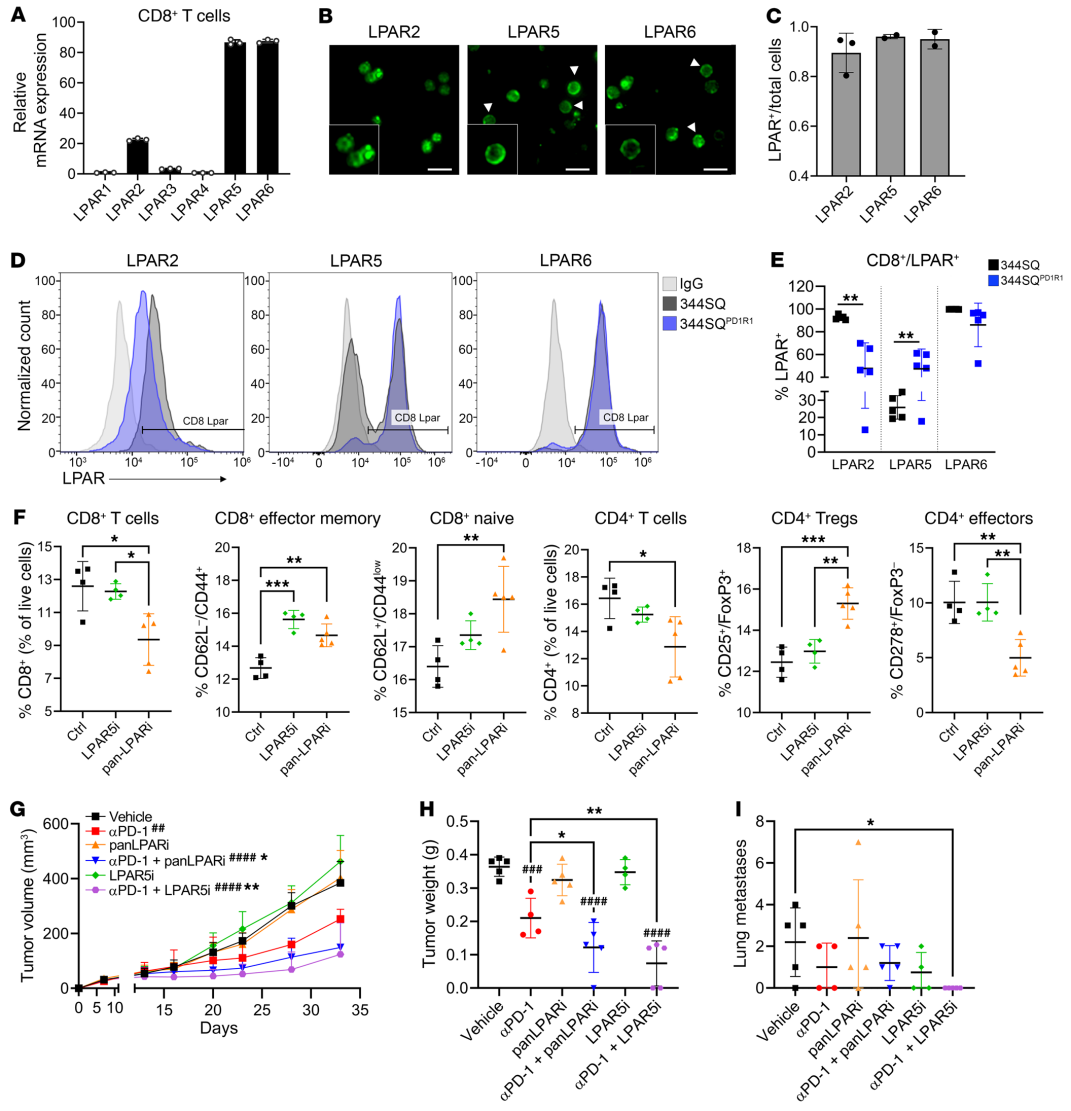
Targeting LPAR5 on CD8^+^ T cells significantly increases effector functions and antitumor activity. (**A**) CD8^+^ T cells were purified from murine spleens and collected for quantitative PCR analysis of LPARs, which were then normalized to LPAR1. (**B**) Immunofluorescence images of LPAR2, LPAR5, and LPAR6 on murine CD8^+^ T cells. Arrowheads denote cells with membranous LPAR. Scale bars: 10 μm; insets zoomed 150%. (**C**) Images from **B** were quantified as a fraction of LPAR^+^ cells compared with total nuclei (DAPI). (**D**) 344SQ and 344SQPD1R1 cells were implanted into mice (*n* = 5 mice each). After 3 weeks, tumors were processed for flow cytometry. Each tumor was separated into 3 samples and stained with LPAR2, LPAR5, or LPAR6. Histograms depict CD8^+^LPAR^+^ cells. An IgG-stained sample is shown as a negative control. (**E**) Quantification of the experiment in **D**, which was completed twice. **P < 0.01, by *t* test. (**F**) 344SQ^PD1R2^ cells were cocultured with naive immune cells and treated with vehicle, LPAR5 inhibitor (AS2717638), or pan-LPAR inhibitor (BrP-LPA). Immune cells were then analyzed by flow cytometry. The experiment was completed twice. *P < 0.05, **P < 0.01, and ***P < 0.001, by 1-way ANOVA. (**G**–**I**) 344SQ cells were implanted into mice and treated with vehicle, anti–PD-1, BrP-LPA alone or with anti–PD-1, or AS2717638 alone or with anti–PD-1. *n* = 5 mice per group. (**G**) Tumor growth was monitored with calipers. ^##^*P* < 0.01 and ^####^*P* < 0.0001, by 1-way ANOVA compared with vehicle; *P < 0.05 and **P < 0.01, by 1-way ANOVA compared with anti–PD-1. (**H**) Tumor weight recorded at necropsy. ^###^*P* < 0.001 and ^####^*P* < 0.0001, by 1-way ANOVA compared with vehicle; *P < 0.05 and **P < 0.01, by 1-way ANOVA compared with anti–PD-1. (**I**) Lung metastases recorded at necropsy. *P < 0.05, by 1-way ANOVA.

Our data have revealed that the ATX/LPA axis contributes to PD-1 blockade resistance, and cotargeting of both axes is efficacious in multiple models of KP lung cancer. However, it is unknown in this model how accumulation of ATX/LPA alters immune functionality, particularly CD8^+^ T cell activation. To determine whether CD8^+^ T cells functionally respond to LPA, we stimulated purified naive CD8^+^ T cells with exogenous LPA and measured downstream signaling, cytokine production, and cellular differentiation. As others have shown, the addition of LPA significantly reduced phospho-ERK activation downstream of antigenic stimuli, IFN-γ secretion, and differentiation into effector memory cells (37, 38) (Supplemental Figure 8, A–C). LPA binds to and activates one of 6 different receptors (LPAR1–6), which are G protein–coupled receptors that can stimulate numerous downstream signaling cascades. Thus, we analyzed the expression of these receptors on CD8^+^ T cells, first focusing on purified naive CD8^+^ T cells (Figure 6A). While all receptors are expressed, LPAR2, LPAR5, and LPAR6 are expressed to the highest degree in a naive setting. We confirmed the expression of these 3 LPARs at the protein level by performing immunofluorescence staining, with 90%–95% of naive CD8^+^ T cells demonstrating positive staining (Figure 6, B and C). Because LPA can promote autocrine activation of key oncogenic pathways, we also analyzed tumor cell expression of LPARs. We found that the 344SQ sensitive and resistant cells expressed several LPAR genes (Supplemental Figure 8D), but when they were stimulated with exogenous LPA, no common downstream signaling cascades appeared to be activated as a result (Supplemental Figure 8E), suggesting that the impact of aberrant LPA within the tumor microenvironment (TME) has primarily paracrine effects.

### CD8^+^ T cells within anti–PD-1 resistant tumors have altered expression of LPAR2 and LPAR5.

While almost all naive CD8^+^ T cells express LPAR2, LPAR5, and LPAR6, we next wanted to analyze the expression of these receptors on tumor-infiltrating CD8^+^ T cells. To do so, we implanted the 344SQ and 344SQ^PD1R1^ models into mice. After 3 weeks, tumors were collected for flow cytometry staining of LPAR2, LPAR5, or LPAR6 specifically on CD8^+^ T cells. Interestingly, we found that the CD8^+^ T cells in the 344SQ model had about 95% positivity for LPAR2, whereas only about 50% had expression in the 344SQ^PD1R1^ model (Figure 6, D and E). Conversely, we observed significantly higher LPAR5 expression on the CD8^+^ T cells from the 344SQ^PD1R1^ tumors compared with the 344SQ model. Lastly, LPAR6 was highly expressed on CD8^+^ T cells irrespective of tumor model. The expression of these receptors on peripheral T cells in a tumor-bearing mouse has not been analyzed, so the possibility exists that these changes may occur prior to tumor infiltration. Despite this, these data provide evidence that not only is the ATX/LPA axis aberrantly upregulated with PD-1 blockade resistance, but the expression of LPAR2 and LPAR5 on infiltrating CD8^+^ T cells may also be altered, and this may further contribute to changes in immune cell functionality in the TME.

### LPAR5 inhibition promotes effector memory CD8^+^ T cells and represses tumor growth and metastasis when combined with anti–PD-1.

To further define the role of LPARs on CD8^+^ T cells, we focused on specifically blocking LPAR5, as this was recently shown to be involved in diminishing TCR signaling (38), and because we found this receptor to be highly expressed on CD8^+^ T cells within anti–PD-1–resistant tumors. Using an LPAR5 inhibitor and a pan-LPAR inhibitor, we performed coculture assays, culturing 344SQ^PD1R2^ cells and naive immune cells together for 4 days in the presence of these inhibitors. We found that both pan-LPAR and LPAR5-specific inhibition promoted the CD8^+^ effector memory population by flow cytometry (Figure 6F). Interestingly, LPAR5 inhibition proved to be more beneficial, especially within the CD4^+^ T cell compartment. Specifically, the pan-LPAR inhibitor significantly decreased the ICOS^+^ effectors and increased the FoxP3^+^CD25^+^ Treg populations (Figure 6F). Thus, the overall immune profile is more favorable with specific LPAR5 targeting, whereas blocking other LPARs may stimulate immunosuppressive populations especially within the CD4^+^ T cell compartment. Further studies are required to better define the pleiotropic effects of LPA on these immune cell subsets.

To support these data with preclinical evidence, we performed an in vivo experiment in the 344SQ model with both LPAR inhibitors, alone and in combination with anti–PD-1. We found that the pan-LPAR and the LPAR5-specific inhibitors worked equally well in controlling primary tumor growth when combined with anti–PD-1 treatment (Figure 6, G–I, and Supplemental Figure 8F). Interestingly, the combination of LPAR5 and PD-1 blockade also significantly inhibited metastatic burden, with complete abolition of metastatic lesions in these mice.

**Figure 7:**
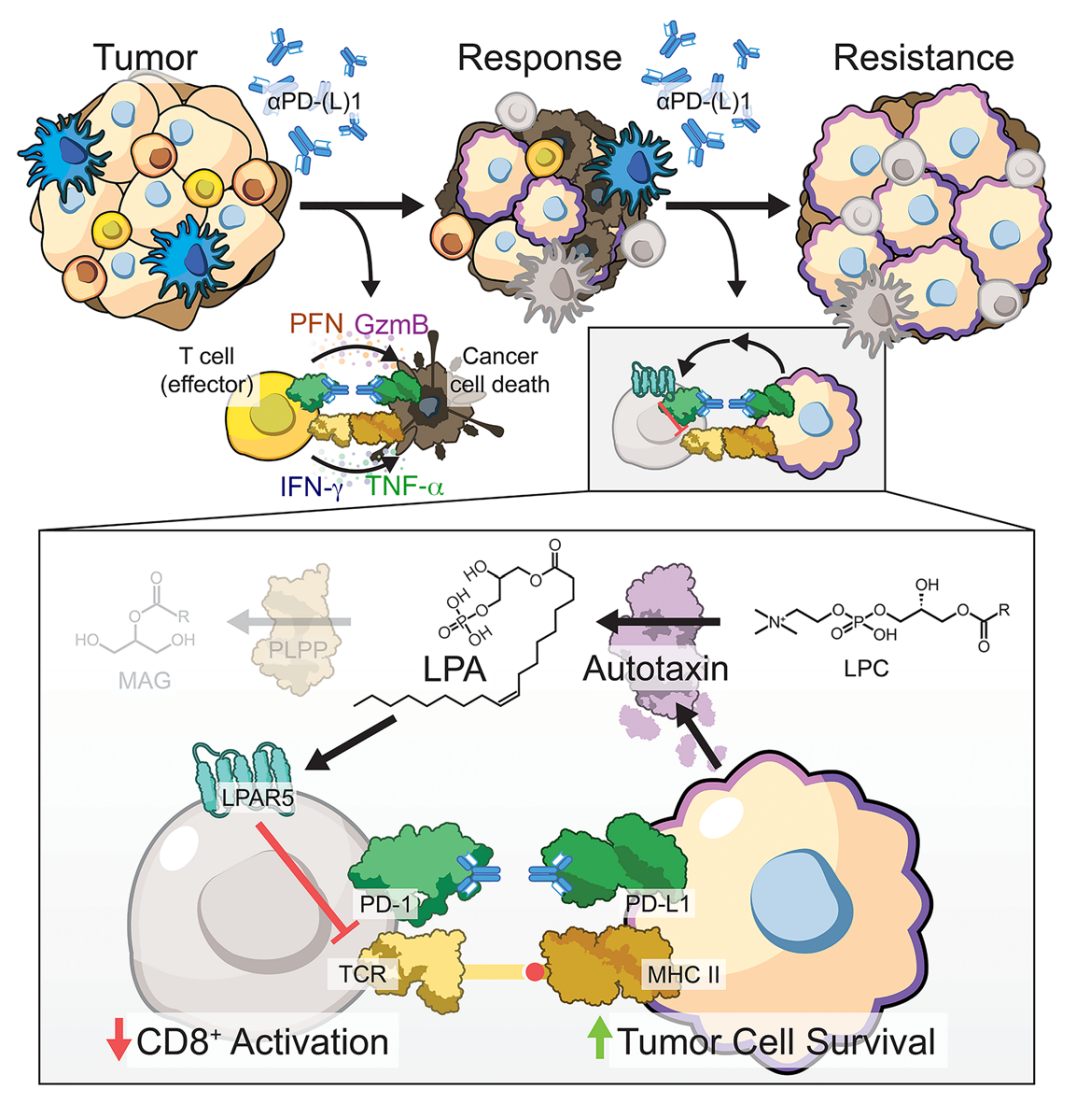
The ATX/LPA axis is upregulated with anti–PD-(L)1 treatment resistance, modulating CD8^+^ T cell functionality via LPAR5 activation. Kras/p53 mutant lung cancers respond initially to PD-1/PD-L1 axis blockade, but eventually acquire resistance. Our data indicate that a robust and stable upregulation of the enzyme autotaxin (ATX) occurs with resistance, which causes an aberrant accumulation of its bioactive metabolite, lysophosphatidic acid (LPA). LPA acts in a paracrine manner on tumor-resident immune cells, particularly the CD8^+^ T cell compartment. Activation of LPA receptor 5 (LPAR5) via LPA diminishes T cell receptor signaling and downstream activation required for effective antitumor functionality, thereby promoting tumor cell survival. Targeting ATX or LPAR5 with anti–PD-1 treatment can promote antitumor immunity by restoring T cell proliferation and activation, leading to more efficacious control of lung cancer growth.

Taken together, these data indicate that dysregulation of the ATX/LPA/LPAR5 axis contributes to PD-1 blockade therapy resistance in KP mutant lung cancer, and cotargeting this immunosuppressive axis efficaciously controls lung cancer progression and metastasis (Figure 7).

## Discussion

In generating and characterizing the KP anti–PD-1–resistant tumor models, we found that they maintain the major pathways and molecules involved in responding to an invigorated immune response despite losing responsiveness to treatment. Specifically, known mechanisms of ICB resistance, including downregulation or loss of JAK/STAT signaling, IFN-γ response, antigen presentation, and PD-L1 expression, were analyzed, and we found these inflammatory pathways to be intact in the anti–PD-1–resistant models. However, flow cytometry analysis of resistant tumors revealed baseline differences in the tumor-infiltrating immune microenvironment in comparison with sensitive tumors, with downregulation of total and effector memory CD8^+^ T cells and a decrease in M1-like macrophages and corresponding increase in M2-like macrophages, indicating broad immunosuppression within the microenvironment of resistant tumors. Importantly, we found the CD8^+^ T cell compartment, and specifically the effector CD8^+^ T cells, to be increased in anti–PD-1 sensitive models with ICB treatment but decreased and unchanged by treatment in the resistant models. Therefore, the anti–PD-1–resistant tumor models provided a unique opportunity to analyze novel tumor-intrinsic mechanisms driving notable immunosuppression and PD-1 blockade resistance.

To this end, we analyzed transcriptomic data from KP tumors with acquired resistance to anti–PD-L1 and discovered the enzyme ATX to be significantly upregulated in an acquired resistance setting and in the newly developed, intrinsically resistant tumor models. In fact, we found many proteins involved in the ATX/LPA pathway to be aberrantly expressed in the anti–PD-1–resistant tumor models compared with sensitive models; however, the underlying mechanisms remain to be elucidated. We found that an inflammatory signature and cytolytic T cell score correlate with increasing *ENPP2* expression in human lung adenocarcinoma patients, which is in line with findings in the literature that inflammatory cytokines like IL-1β, TNF-α, and IFN-γ can promote ATX expression (31, 32). However, these data are limited by a lack of temporal information to link immune activation with ATX expression and a lack of longitudinal ICB therapy samples in human lung cancer patients to further connect the experimental model results with the more relevant human disease. Additionally, these data fail to explain how the anti–PD-1–resistant cell lines maintain high expression of ATX, especially considering that other mechanisms of acquired resistance such as upregulation of CD38 are not maintained after ex vivo culturing. Therefore, additional analyses of known ATX regulators are required to dissect the mechanism by which it becomes stably increased upon development of ICB treatment resistance. Additionally, we found a concurrent downregulation of the *Plpp1*–*Plpp3* enzymes in anti–PD-1–resistant tumors, which are important for rapid degradation of LPA, further contributing to aberrant accumulation of LPA within these tumors, another result that requires further exploration. Lastly, we also discovered disparate expression of LPAR2 and LPAR5 on CD8^+^ T cells within the microenvironment of anti–PD-1–resistant tumors, with significant downregulation of LPAR2 and upregulation of LPAR5 in comparison with CD8^+^ cells within sensitive tumors. However, as we did not analyze systemic CD8^+^ T cells from tumor-bearing mice for their expression of LPARs, it is unclear at this time whether these alterations are found on T cells in the periphery as well. These data are the first to our knowledge to demonstrate LPAR expression differences on tumor-infiltrating CD8^+^ T cells as a function of anti–PD-1 treatment resistance and could reveal important biology about the involvement of these receptors in trafficking, activation, and cytotoxicity. Future studies will aim to reveal the underlying mechanisms controlling the expression of these various ATX/LPA axis members.

We found that tumor cells expressing ATX can directly impact immune functionality via local accumulation of LPA in the TME. Specifically, ATX knockdown in a representative 344SQ^PD1R^ tumor line significantly delayed tumor growth and increased total and effector intratumoral CD8^+^ T cells, whereas overexpression of ATX suppressed these populations and was sufficient to create intrinsic resistance in a sensitive model. The role of the ATX/LPA axis in tumor progression has largely focused on its being a metastatic driver (39–42), with the discovery of ATX arising from analysis of metastatic versus non-metastatic melanoma conditioned media samples (43). However, more recent data have begun to highlight the paracrine effects that ATX and LPA have on tumor-resident immune cells. LPA has previously been shown to influence naive T cell migration and TCR-activated IL-2 secretion (44, 45), activated NK cell functions (46), and immature dendritic cell migration (44), indicating pleiotropism in response that can be favorable for antitumor activity. However, additional studies revealed that LPA activation of LPAR5 on CD8^+^ T cells negatively regulates T cell receptor signaling via disrupted calcium mobilization (37, 38), which we corroborated in our data. In addition, another group discovered that LPAR6 can negatively regulate T cell migration and found a significant inverse correlation between RNA levels of ATX and CD8^+^ T cell infiltration in melanoma patients (47). Our data show similar trends in murine models of KP lung cancer; however, we did not specifically analyze the role of LPAR6 in contributing to T cell migration. Additional studies have focused on the impact of LPA on the extracellular cytokine milieu, with LPA promoting the transcription of IL-6 and IL-8 in breast and ovarian cancer cells (48, 49), while also diminishing type I interferon responses in ovarian cancer (50). Our models vary from these data in that interferon responses are largely unaffected between anti–PD-1 sensitive and resistant models, although we did find direct effects of LPA on cytokine secretion. Additional analyses on the cytokine milieu as a function of ATX/LPA expression need to be completed, as these may be contributing factors to diminished CD8^+^ T cell infiltration and activation. Taken together, these studies and ours indicate a broader applicability of the immunomodulatory role of this axis and highlight the importance of dissecting the involvement of specific LPARs in various tumor-infiltrating immune cells.

Cotargeting ATX with anti–PD-1 treatment not only controlled KP lung tumor growth but also promoted tumor regressions in both syngeneic and autochthonous KP tumor models. These results were mirrored by specific blockade of LPAR5, which likely plays an immunosuppressive role on CD8^+^ T cells by blocking TCR signaling as discussed above. However, analysis of immune cell subsets cultured with anti–PD-1–resistant tumor cells in the presence of a pan-LPAR inhibitor revealed that some immunosuppressive populations are affected by LPA, as pan-LPAR blockade increased suppressive CD4^+^ Tregs and decreased effector CD4^+^ cells. Thus, better understanding of LPA-related effects on the CD4^+^ T cell compartment will be necessary. Additionally, evidence in the literature indicates that LPA can have proinflammatory effects such as promotion of naive T cell calcium mobilization and chemotaxis (44, 45). While we achieved efficacious results with the ATX inhibitor when given as an upfront combination, a staggered treatment schedule may be more beneficial to allow for increased homing of naive cell subsets to the tumor before subsequently inhibiting LPA generation to achieve the best immune-mediated tumor cell killing. Additional preclinical studies will be imperative to support the combinatorial treatment strategy of ATX/LPAR inhibition with ICB to prevent resistance and control the progression of lung cancer.

## Methods

### Cell lines.

All cell lines were cultured at 37°C in a humidified incubator at 5% CO_2_ and maintained in RPMI 1640 plus 10% FBS. Mycoplasma-negative cells were used for all experiments and tested regularly using LookOut Mycoplasma PCR Detection Kit (MilliporeSigma).

The 344SQ Kras/p53 mutant murine cell line was created previously (16). To generate the anti–PD-1 sensitive and resistant derivatives, the 344SQ cells were implanted subcutaneously into 129S2/SvPasCrl mice (referred to as 129/Sv; Charles River). After 1 week, mice were treated with anti–PD-1 blocking or IgG control antibodies weekly. After 5–6 weeks of treatment, mice were sacrificed, and tumors excised. Tumor tissue was sterilized using povidone-iodine (Betadine) wash for 1 minute. Several Dulbecco’s PBS rinses were used to remove residual iodine. Tumor tissue was cut into 1- to 2-mm fragments and placed in a tissue culture dish with RPMI plus 10% FBS plus 1% penicillin/streptomycin. Once cells began to grow (usually around day 4–7), the tumor fragments were removed. Cells were subcultured several times to obtain a single cell layer. At passage number 5, cell lines were tested for mycoplasma and antibiotics were removed. Cells were passaged until passage 10 to obtain a cell line composed primarily of tumor cells. To create the KP^IgG^ and KP^PDL1^ lines, the Kras^LA1-G12D^/p53^R172HΔg^ constitutive GEMM was imaged by CT scan to confirm tumor burden. Mice were treated with anti–PD-L1 or IgG control antibody for 4 weeks. After treatment, mice were sacrificed, and lung tissue was examined for tumors. The largest tumors were dissected from the lungs and processed following the protocol above. Generation of the ATX-overexpressing and ATX-knockdown cells is described in Supplemental Methods.

### Animal studies.

All animal studies were completed under the approval of the University of Texas MD Anderson Cancer Center Institutional Animal Care and Use Committee (IACUC) (protocol 1271) or the Emory University IACUC (protocol 201700322). Murine lung cancer cells were implanted subcutaneously into the right flank of 129/Sv male or female mice between 3 and 6 months of age. Tumor growth was measured via calipers beginning at 1 week after implantation. The constitutive Kras^LA1-G12D^/p53^R172HΔg^ mice were generated as previously described (16, 51), and the Kras^LSL-G12D/+^/p53^wm-R172H/wm-R172H^ mice (52) were infected starting at 4 months of age with a titer of 2.5 × 10^7^ PFU adenovirus Cre (Ad5-CMV-Cre) using the method described previously (53). Disease was allowed to progress until it was deemed inhumane to let it continue. Both male and female mice were used for these studies. Genotyping of the mice was done on ear snips obtained from the mice at time of weaning (21 days). Ear snips were digested with and according to the QuantaBio Extracta DNA Prep for PCR. Dirty DNA was then used for PCR using Apex Hot Start Taq BLUE Master Mix, 2×, and primers as listed in Supplemental Table 2.

### Drug treatments.

Anti–PD-1 and anti–PD-L1 antibodies were purchased from Bio X Cell (clone RMP1-14 and clone 10F.9G2, respectively), and treatments were given once weekly via i.p. injection at 200 μg/treatment in 100 μL total volume. PF-8380 was purchased from Selleckchem and dissolved in 1% CMC-Na plus 0.9% NaCl. Mice were dosed orally daily at 50 mg/kg in 100 μL. BrP-LPA was purchased from Echelon Biosciences and was dissolved in sterile 0.9% saline. Treatments were given at 1 mg/mL twice weekly via 100 μL i.p. injection. AS2717638 was purchased from MedChemExpress and was dissolved in the following: 10% DMSO plus 40% PEG300 plus 5% Tween-80 plus 45% saline. Mice were treated orally daily at 10 mg/kg in 100 μL. For syngeneic tumor experiments, treatments were started after palpable tumor formation at 1 week after implantation. For GEMM treatments, once lung tumors were confirmed by CT scanning, mice were randomly enrolled into treatment arms and treated for 4 weeks. The pretreatment CT image was used for baseline measurements, and the week 4 image was used as the endpoint measurement. The percentage difference between these time points was calculated for each mouse. In vitro drug treatment information can be found in Supplemental Methods.

### Flow cytometry.

For flow cytometry on intratumoral immune cell populations, tumors were processed as described previously (54). Briefly, tumors were chopped using a sterile scalpel until 2–3 mm in size, then placed in digestion media containing collagenase I (0.05% wt/vol; MilliporeSigma), DNase type IV (30 U/mL; MilliporeSigma), and hyaluronidase type V (0.01% wt/vol; MilliporeSigma). Mechanical dissociation using the gentleMACS Octo Dissociator (Miltenyi Biotec) was performed followed by a 40-minute incubation at 37°C. Tumor samples were mechanically dissociated again, then passed through a 70 μm filter. RBC lysis (BioLegend) was performed on tumor cell suspension following the manufacturer’s recommendations. For the ATX inhibitor plus anti–PD-1 flow studies, 344SQ tumor samples were stained with a 34-color panel and acquired using a Cytek Aurora. Antibodies and dilutions are listed in Supplemental Table 2. Additional details can be found in Supplemental Methods.

### Analysis of ENPP2 in human data sets.

RNA-Seq and clinical attribute data from the Immunogenomic Profiling of Non–Small Cell Lung Cancer Project (ICON) were analyzed from patient tumor and matched uninvolved tissue. Samples underwent processing and analysis as previously described (55–59). Expression of *CD8A*, *GZMA*, and *PRF1* from the RNA-Seq data was used to generate a composite score representative of T cell cytolytic score (CYT) activity. These genes were selected based on previously reported findings from our group (17), and the score was calculated using single-sample gene set enrichment analysis (60). ICON samples were divided into 3 groups, low, neutral, and high, based on observed breaks in the distribution of CYT scores. *ENPP2* expression reads per kilobase million (RPKM) was then compared across the 3 groups, and Wilcoxon’s rank-sum testing was performed for each pair of groups. For this analysis and all subsequent analysis of the ICON data set, 2 outlier data points based on *ENPP2* expression were removed from consideration. *ENPP2* expression was also examined alongside clinical attributes including overall survival, recurrence, histology group, stage, treatment received, and non-synonymous tumor mutational burden. The BATTLE-2 data set (89 patients) was generated and accessed as previously described (17, 29), and the TCGA data set (515 patients) was accessed from cBioPortal based on data generated by the TCGA Research Network. The software R (version 3.5.1) was used to perform all statistical analyses. The pretreatment and on-treatment melanoma samples were accessed from the previously published and deposited data set GSE91061 (Gene Expression Omnibus [GEO]) (34). Of those patients, 32 had progressive or stable disease, and 9 had partial or complete responses. In each of these categories of response, the delta in *ENPP2* expression was calculated by subtraction of on-treatment expression values from pretreatment expression values.

### Statistics.

Unpaired 2-tailed Student’s *t* tests were performed for all statistical analysis with 2 comparisons, 1-way ANOVA for comparisons with 3 or more groups, and 2-way ANOVA for grouped analyses including tumor growth curves. Tukey’s correction was used to correct for multiple comparisons unless otherwise stated. A *P* value of less than 0.05 was considered statistically significant. Error bars represent standard deviation around the mean unless otherwise noted. All analyses were performed in GraphPad Prism (v9.3.1) unless otherwise noted.

### Study approval.

All animal studies were completed under the approval of the University of Texas MD Anderson Cancer Center Institutional Animal Care and Use Committee (IACUC) (protocol 1271) or the Emory University IACUC (protocol 201700322). Informed consent for ICON was provided via protocol PA15-1112, which was approved by the University of Texas MD Anderson Cancer Center Institutional Review Board (IRB). Informed consent for the BATTLE-2 study was provided by all patients and was approved by the University of Texas MD Anderson Cancer Center IRB as previously described (29).

### Data availability.

Mouse transcriptome data were previously collected and published (17). The ICON patient data set can be accessed from the previously published ICON data browser (61). The melanoma patient data were previously deposited to GEO at NCBI and are publicly available under accession number GSE91061. Data included in this article are provided in the Supporting Data Values file and are also available upon request from the authors.

Additional methodology details are described in Supplemental Methods.

## Author contributions

JMK designed research studies, acquired and analyzed data, wrote and edited the manuscript, and acquired funding. BLR designed research studies, acquired and analyzed data, and edited the manuscript. HW conducted experiments and acquired and analyzed data. JJF prepared figures, acquired and analyzed data, and edited the manuscript. LG acquired data and designed research studies. LD analyzed data and prepared figures. JW provided resources and software, supervised the research, acquired funding, and reviewed the manuscript. SS analyzed data, prepared figures, and reviewed and edited the manuscript. IIW and JZ acquired data and funding. DLG designed research studies, supervised the research, acquired funding, and edited and reviewed the manuscript.

## Supplementary Material

Supplemental data

Supplemental table 1

Supplemental table 2

Supporting data values

## Figures and Tables

**Table 1:**
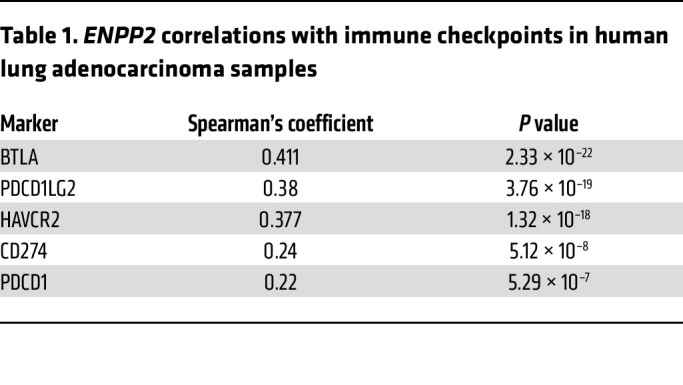
*ENPP2* correlations with immune checkpoints in human lung adenocarcinoma samples

## Version 1 09/01/2023

Electronic publication
