# Supplementary material for: Autotaxin suppresses cytotoxic T cells via LPAR5 to promote anti–PD-1 resistance in non–small cell lung cancer
Source: J Clin Invest. 2023 Sep 1;133(17):e163128. doi: 10.1172/JCI163128 (PMC10471170; doi:10.1172/JCI163128)

Supplemental Methods

Cell Lines

To create the 344SQ-ATX overexpressing cells, ATX was subcloned from the pCMV6-Enpp2 plasmid from Origene (Cat# MR223207) using SalI-HF and MluI-HF and ligated into the pLenti-puro plasmid using XhoI and MluI-HF. This sequenced plasmid was transfected into HEK293T cells with psPAX2 and pMD2G packaging plasmids to create lentiviral particles which were then added to the 344SQ parental cell line. Puromycin was used to select for transduced cells (5 µg/ml). The $344SQ^{PD1R2}$-shATX knockdown cells were created using pLKO.1-shRNA plasmids ordered from Sigma, targeting murine *Enpp2*. Viral transduction of *Enpp2* targeting and non-targeting control shRNAs was performed as described above with the $344SQ^{PD1R2}$ cell line (puromycin 10 µg/ml).

In vitro Drug/Compound treatments

For in vitro treatments, PF-8380, BrP-LPA, and AS2717638 were reconstituted in DMSO according to their solubility profiles, and DMSO was used as the vehicle control for these experiments. 1-Oleoyl lysophosphatidic acid (LPA, 18:1) was purchased from Cayman Chemicals as a solution in ethanol (cat. # 10010093). Lysophosphatidylcholine (LPC, 18:1) was purchased from Avanti Polar Lipids as a solution in chloroform (cat. # 845875). To CD8+ T cells, LPA was added at 1 µM pre-stimulation with anti-CD3 and anti-CD28 antibodies. To tumor cells, LPA was added at either 1 or 10 µM concentrations and cells were collected for western blot after 15 minutes of incubation. Ethanol was used as a vehicle control.

In vitro immune cell assays

Spleens were collected from wildtype 129/sv mice and processed via 40 µm filtration of ground organ. Red blood cells were lysed using 1x RBC lysis buffer (10x buffer, Biolegend), then resulting

splenocytes were cryopreserved in 90% FBS + 10% DMSO. For co-cultures, splenocytes were thawed, counted, and stimulated with 3 µg/mL of anti-CD3 and anti-CD28 (Invitrogen). Splenocytes were plated onto tumor cells in 24 well plates in replicates and cultured for 4-5 days at 37°C at 1:10 ratio. After culturing, splenocytes were collected from the media and stained for flow cytometry, described below.

For CD8+ T cell isolation, splenocytes were thawed from cryopreservation. CD8+ T cells were purified using a CD8+ T cell negative isolation kit (Miltenyi) following the recommended protocol. After magnetic sorting, purified CD8+ T cells were then stimulated with 3 µg/mL of anti-CD3 and anti-CD28 alone or in the presence of 1 µM LPA. Samples were incubated for various times over a 60-minute time course of stimulation, at which point they were fixed using 1% paraformaldehyde. These samples were then stained for flow cytometry analysis, described below.

Flow cytometry

Immune cells collected from co-culture assays were plated into round bottom 96 well plates and stained with cell membrane T cell markers as described previously (25). After membrane staining, samples were washed, fixed, permeabilized, and then stained with intracellular markers. The purified CD8+ T cells stimulated with anti-CD3 and anti-CD28 with and without LPA were fixed and permeabilized before staining with a phospho-ERK antibody. An IgG control antibody was used for one sample as a negative control. Cells were then washed and stained with AlexaFluor-488 secondary. An unstained control was also included. These samples were acquired using the BD LSR Fortessa (BD Biosciences) and analyzed using FlowJo software (version 10.6.1).

For LPAR staining of CD8+ T cells, 344SQ and 344SQ^{PD1R1} tumors and spleens were collected and processed as described in methods section. Single cell suspensions were then stained with

a live/dead marker, CD45, CD3, CD8 and CD4 antibodies. Samples were fixed and permeabilized, then separated into 3 different tubes. Each one was stained with a different LPAR antibody (LPAR2, LPAR5, or LPAR6). An isotype control was used to account for non-specific binding. After washes, the experimental samples and the IgG controls were stained with a fluorophore conjugated secondary antibody (AlexaFluor-488). Two additional controls included were an unstained control and an LPAR fluorescence minus one (FMO) sample, which included all T cell markers without the LPAR antibody, IgG control antibody, or the secondary antibody. Samples were acquired using BD LSR Fortessa (BD Biosciences) and analyzed using FlowJo software.

Semi-quantitative real-time PCR

RNA was extracted from cells in culture using Trizol Reagent (Thermo Fisher) following the recommended protocol. All RNA samples were quantified, and reverse transcription was performed with 2 µg of RNA using qSCRIPT cDNA SuperMix (Quantabio). Real-time PCR was performed using primer sets specific for each gene (sequences listed in Table S2) and the SYBR® Green PCR Master Mix (Life Technologies). L32 (60S ribosomal gene) was used to normalize expression across samples.

Western blot

For the conditioned media samples collection, cells were plated in a 6 well plate at equal cell numbers, grown overnight to ~95% confluency, then the media was changed from complete media to serum free media and incubated for 24 hours. The media was collected, and 1 mL was processed using acetone precipitation. Samples were stored at -80°C overnight. The samples were then spun at 14,000 rpm for 10 min at 4°C and precipitated protein was resuspended in RIPA buffer. Protein from adherent cells was collected in 1X RIPA buffer (10x from Cell Signaling) containing protease and phosphatase inhibitors. Samples were sonicated then spun to collect the

supernatant. Cell lysates and conditioned media samples were boiled for 5 min at 95°C in 1x Laemmli buffer. The samples were run on polyacrylamide gels and transferred to nitrocellulose membranes. Membranes were blocked in 10% non-fat dry milk and antibodies were diluted in 1%BSA/TBST/Sodium azide solution and incubated overnight at 4°C (antibodies and dilutions listed in Table S2). Secondary antibodies were added the next day after washes, and signal was observed using ECL reagents and a film processor.

Immunohistochemistry

Paraffin-embedded tumor sections were processed following a standard protocol. Following heat-mediated antigen retrieval (Dako Technologies) and blocking with 5% goat serum, the primary antibodies were added to tissue based upon manufacturer recommendations. Images were acquired using brightfield microscopy on the Olympus IX73. For the CD8 and granzyme B stains, ImageJ software was used to quantify the number of positive cells per field of view. Image deconvolution was used to view DAB staining separately from the nuclear stain, and a threshold was applied to all images. The "analyze particles" feature was used to quantify the number of positively stained cells per field of view.

Migration and invasion assays

Transwell migration of 8 µM inserts (BD-Biosciences) and invasion (BD-Bioscience) assays were performed for 16 hours as previously described(25). Cells were plated in 200 µl and placed in 24 well plates as per standard protocol(16). Inserts were imaged using crystal violet solution and migratory or invasive cells analyzed on an Olympus IX73 microscope and counted using ImageJ software. Four fields of view were imaged per transwell insert for quantitation.

Single cells were seeded on a matrix comprised of Matrigel (BD-Biosciences) or a Matrigel/Collagen (1.5 mg/ml) mixture (BD-Biosciences) (53). Media was replenished every 48

hours. Spheres were imaged using an inverted Olympus IX73 microscope. ImageJ software was used for analysis. Invasive spheres were counted and graphed as a percentage of total spheres per field of view.

ELISA

The total amount of LPA was measured in conditioned media of cells using a competitive ELISA kit (LS Bio, Cat# LS-F25111-1). Tumor cells were plated in equal numbers in a 96 well plate, incubated overnight and then media was aspirated and replaced with fresh media. For ATX inhibitor treatments, DMSO control or PF-8380 (1 μM) was added at this time. Cells were then incubated for an additional 24 hours at which point, the conditioned media was collected and 50 μl was added directly to the ELISA plate for analysis. For analysis of LPA levels in tumor samples, snap frozen tumors treated with either vehicle control or the ATX inhibitor were weighed, minced on ice into ~1-3 mm pieces, collected into a microcentrifuge tube. The dish was then rinsed with 100 μl of ice-cold PBS and combined with minced tumor samples. Samples were centrifuged at 440 x rcf for 10 minutes at 4°C. 50 μl of supernatant was carefully removed and added directly to the LPA ELISA plate. The protocol was followed as recommended by the manufacturer.

For IFN-γ ELISAs (mouse and human kits from Biolegend, Cat# 430804 and 430104, respectively), human PBMCs extracted from normal donor whole blood or splenocytes extracted from wildtype mice (as described above) were plated in 6 well plates coated with 1 μg/ml anti-CD3. Anti-CD28 (3μg/ml) was added to the complete growth media. Cells were plated with control or LPA (18:1) at 20 μM. Unstimulated cells (without anti-CD3/CD28) were used as a negative control. After 24 hours, 200 μl of media was collected from each condition and diluted (1 to 5 for mouse samples, 1 to 20 for human samples). The diluted samples were then added to a 96 well plate pre-coated with the IFN-γ capture antibody included with the kits. The protocol was then followed as recommended by the manufacturer.

Immunofluorescence

Cryopreserved splenocytes, collected as described above, were thawed and CD8+ T cells were specifically extracted using the CD8+ T cell Isolation Kit from Miltenyi. Purified CD8+ T cells were then plated at 100,000 cells per well in a 24 well plate containing glass coverslips. The plate was spun for 10 min at 2,000 rpm to adhere T cells to the coverslips. Cells were then fixed in 1% paraformaldehyde for 15 min at room temperature, washed with glycine, then permeabilized in 0.1% triton-X. Blocking was performed using 5% normal goat serum for 1 hour, then individual LPAR antibodies were added overnight. Secondary was added the next day and Alexa-Fluor 488 goat anti-rabbit was used for all. A control well with secondary only was used to control for non-specific secondary binding. Coverslips were mounted onto glass slides using Prolong-Gold with DAPI to stain the cell nuclei. Images were obtained using an Olympus IX71 widefield microscope. Quantification of LPAR+ cells was done using ImageJ software. A threshold was applied to images and the “analyze particles” feature was used to identify LPAR+ cells on the GFP channel and the total number of cells on the DAPI channel.

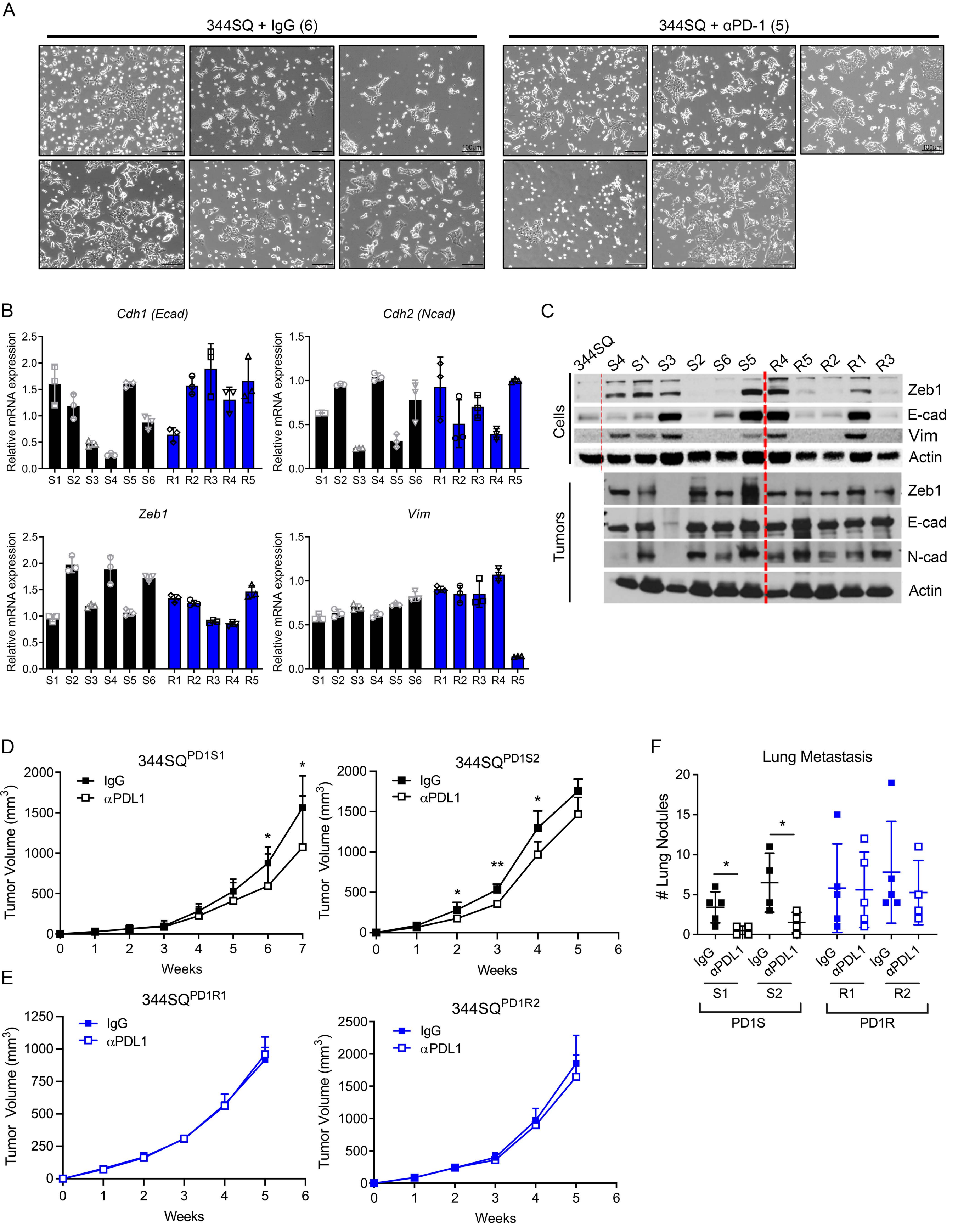
A
344SQ + IgG (6)
344SQ + αPD-1 (5)
100μm
B
Cdh1 (Ecad)
Cdh2 (Ncad)
Zeb1
Vim
Relative mRNA expression
S1 S2 S3 S4 S5 S6 R1 R2 R3 R4 R5
C
344SQ S4 S1 S3 S2 S6 S5 R4 R5 R2 R1 R3
Cells
Tumors
Zeb1
E-cad
Vim
N-cad
Actin
D
344SQ^PD1S1
344SQ^PD1S2
IgG
αPDL1
Tumor Volume (mm^3)
Weeks
E
344SQ^PD1R1
344SQ^PD1R2
F
Lung Metastasis
Lung Nodules
IgG αPDL1
S1 S2 R1 R2
PD1S
PD1R

Fig S1. 344SQ^{PD1R} cells display no overt differences in morphology or EMT status and are resistant to anti-PD-L1 re-challenge. A. 344SQ lung cancer cells were treated in vivo with either IgG control or anti-PD-1 antibody until the development of resistance, at which point tumors were excised and cell lines developed from them (see Figure 1A). Phase contrast microscopy was performed to demonstrate the morphology of the 6 different IgG treated cell lines and the 5 different anti-PD-1 treated cell lines. **B.** The 6 IgG treated 344SQ cell lines (termed 344SQ$^{PD1S1-6}$ or S1-6) and the 5 anti-PD-1 treated 344SQ cells lines (termed 344SQ$^{PD1R1-5}$ or R1-5) were collected for RNA. QPCR analysis was performed to analyze the expression of the epithelial marker E-cadherin (Cdh1) and the mesenchymal markers N-cadherin (Cdh2), Vimentin, and Zeb1. L32 was used as an internal normalization control, and values were then normalized the parental 344SQ expression levels. **C.** The anti-PD-1 sensitive and resistant models (cell lines and tumors) were collected for protein and analyzed via western blotting for epithelial and mesenchymal markers described in B. Actin was used as a loading control. Red dashed line used to demarcate the sensitive versus resistant samples. **D-E.** Two representative 344SQ anti-PD-1 sensitive and resistant cell lines (S1 and S2; R1 and R2) were implanted into mice and treated with either IgG control or anti-PD-L1 antibody. n = 4-5 mice/ group. **D.** Tumor growth was measured weekly via caliper measurements. The 2 sensitive models are shown on top, and the 2 resistant models are shown below. *$p<0.05$, **$p<0.01$ by multiple *t* tests (one per timepoint). **E.** Total macroscopic lung metastases were counted at necropsy. *$p<0.05$ by *t* test.

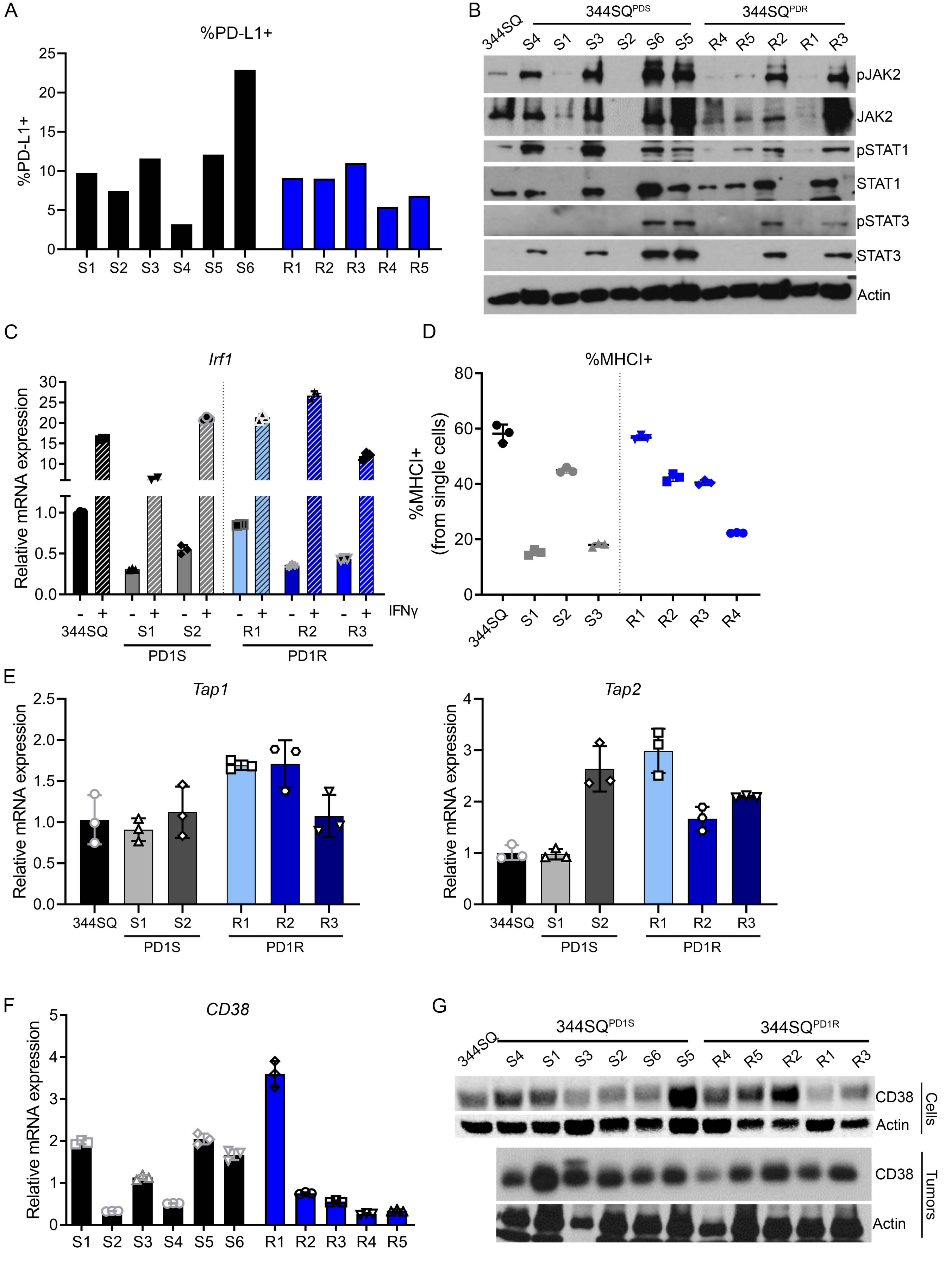

A
%PD-L1+
%PD-L1+
0
5
10
15
20
25
S1 S2 S3 S4 S5 S6 R1 R2 R3 R4 R5
B
344SQ
344SQ^PDS
344SQ^PDR
S4 S1 S3 S2 S6 S5 R4 R5 R2 R1 R3
pJAK2
JAK2
pSTAT1
STAT1
pSTAT3
STAT3
Actin
C
Irf1
Relative mRNA expression
0.0
0.5
1.0
10
15
20
25
30
- + - + - + - + - + - +
IFNγ
344SQ
S1
S2
R1
R2
R3
PD1S
PD1R
D
%MHCI+
%MHCI+ (from single cells)
0
20
40
60
80
344SQ S1 S2 S3 R1 R2 R3 R4
E
Tap1
Relative mRNA expression
0.0
0.5
1.0
1.5
2.0
2.5
344SQ S1 S2 R1 R2 R3
PD1S
PD1R
Tap2
Relative mRNA expression
0
1
2
3
4
344SQ S1 S2 R1 R2 R3
PD1S
PD1R
F
CD38
Relative mRNA expression
0
1
2
3
4
5
S1 S2 S3 S4 S5 S6 R1 R2 R3 R4 R5
G
344SQ
344SQ^PD1S
344SQ^PD1R
S4 S1 S3 S2 S6 S5 R4 R5 R2 R1 R3
CD38
Actin
Cells
CD38
Actin
Tumors

Fig S2. 344SQ^{PD1R} cells maintain key inflammatory molecules and pathways, including PD-L1 expression, IFNγ response and antigen presentation machinery. A. The cell surface expression of PD-L1 was measured on 344SQ$^{PD1S1-6}$ and 344SQ$^{PD1R1-5}$ cells via flow cytometry. The value is graphed as the percentage of total live single cells. **B.** 344SQ$^{PD1S1-6}$ and 344SQ$^{PD1R1-5}$ cells were collected for protein and analyzed via western blotting for phosphorylated and total levels of JAK2, STAT1, and STAT3. Actin was used as a loading control. **C.** The parental 344SQ, 2 of the anti-PD-1 sensitive (S1 and S2) and 3 of the anti-PD-1 resistant (R1-3) cell lines were grown in culture with the addition of murine IFNγ to the culture media or in control media. After 48 hours, cells were collected for qPCR analysis of Irf1. L32 was used as an internal control, and all samples are normalized to the 344SQ control (no IFNγ) sample. **D.** The 344SQ parental cells or representative anti-PD-1 sensitive and resistant lines were collected and analyzed via flow cytometry for surface expression of MHCI. The values are graphed as a percentage of total live single cells. **E.** The Tap1 and Tap2 gene expression was analyzed using qPCR in the 344SQ parental, PD-1 sensitive (S1 and S2) and the PD-1 resistant (R1-3) cells. **F.** The 344SQ anti-PD-1 sensitive (S1-6) and anti-PD-1 resistant (R1-5) cells were analyzed via qPCR for the expression of CD38. L32 was used as an internal normalization control, and values were then normalized the parental 344SQ expression levels. **G.** The protein expression of CD38 was analyzed across the 344SQ anti-PD-1 sensitive and resistant panels (cell lines and tumors) via western blotting. Actin was used as a loading control. The cell line actin blot was also shown in Figure S1C.

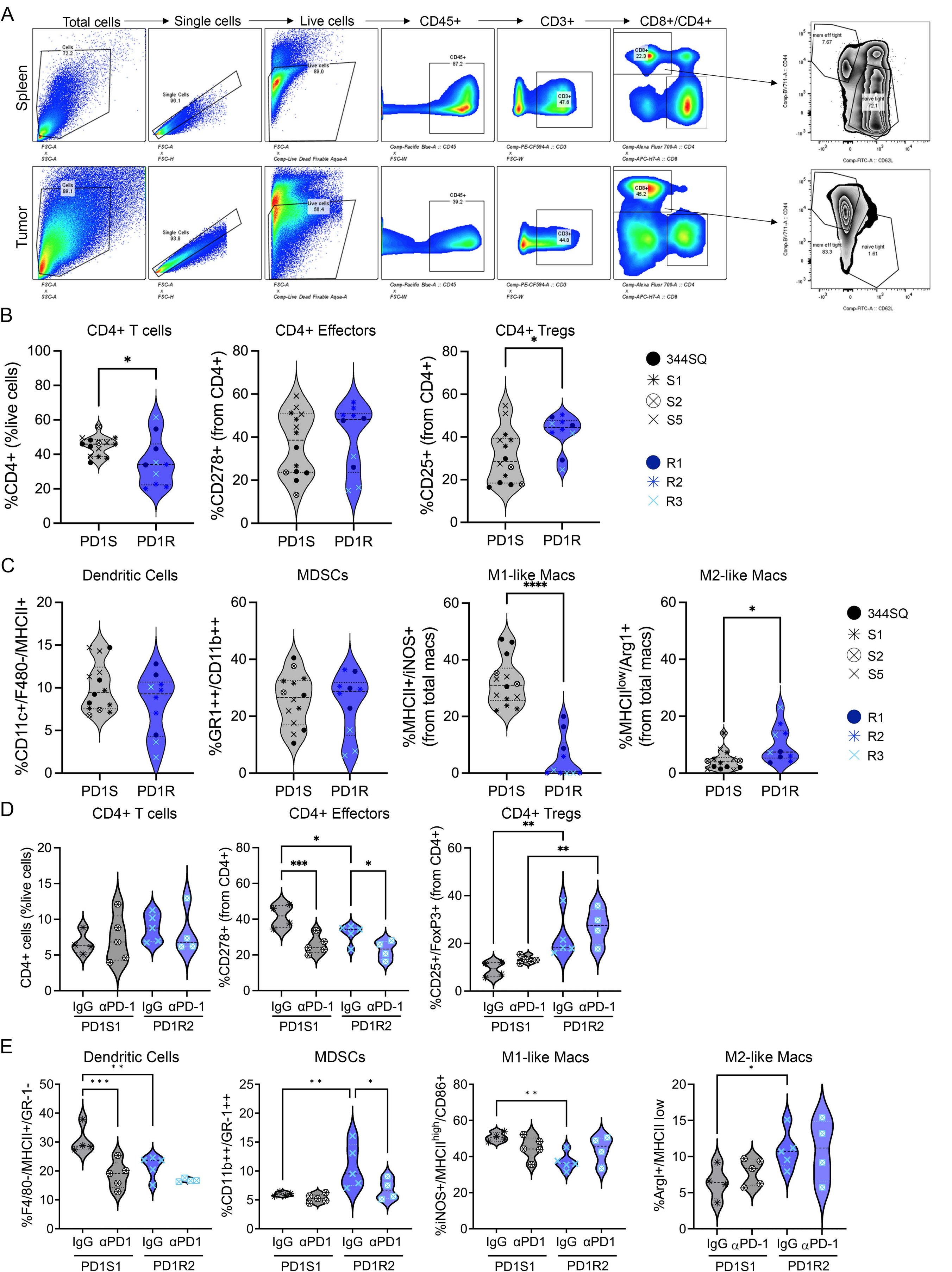
A
Total cells
Single cells
Live cells
CD45+
CD3+
CD8+/CD4+
Spleen
Tumor
B
CD4+ T cells
CD4+ Effectors
CD4+ Tregs
%CD4+ (%live cells)
%CD278+ (from CD4+)
%CD25+ (from CD4+)
PD1S
PD1R
344SQ
S1
S2
S5
R1
R2
R3
C
Dendritic Cells
MDSCs
M1-like Macs
M2-like Macs
%CD11c+/F480-/MHCII+
%GR1++/CD11b++
%MHCII+/iNOS+ (from total macs)
%MHCIIlow/Arg1+ (from total macs)
D
CD4+ cells (%live cells)
%CD25+/FoxP3+ (from CD4+)
IgG
αPD-1
PD1S1
PD1R2
E
%F4/80-/MHCII+/GR-1-
%CD11b++/GR-1++
%iNOS+/MHCIIhigh/CD86+
%ArgI+/MHCII low
αPD1

Fig S3. Flow cytometry analysis of anti-PD-1 resistant tumor models revealed increased CD4+ Tregs and M2-like macrophages with resistance. A. Gating schema for immunophenotyping of anti-PD-1 sensitive and resistant tumor and spleens. **B.** The tumors generated from the 344SQ, $344SQ^{PD1S}$, and $344SQ^{PD1R}$ models were analyzed via flow cytometry for tumor-infiltrating CD4+ subsets (Figure 2A). CD4+ T cells are graphed as a percentage of total T cells. CD4+ effectors (CD278+) and CD4+ Tregs (FoxP3+/CD25+) are graphed as a percentage of CD4+ T cells. *p<0.05 by *t* test. **C.** The myeloid populations from the experiment described in Figure 2A were also analyzed. Dendritic cells are depicted as a percentage of CD11c+ (from CD45+). Myeloid-derived suppressor cells (MDSCs) are graphed as a percentage of the CD45+ subset. Macrophages are gated underneath CD45+/CD11b+ cells. Total macrophages (not shown) are then divided into M1 (iNOS+/MHCII+/CD86+) or M2 (Arg1+/MHCII-). *p<0.05, ****p<0.0001 by *t* test. **D-E.** Additional flow cytometry analysis from the experiment depicted in Figure 2D. **D.** CD4+ T cell subsets are shown as described in panel B. **E.** Myeloid cell populations were analyzed as described in panel C. *p<0.05, **p<0.01, ***p<0.001, ****p<0.0001 by 1-way ANOVA.

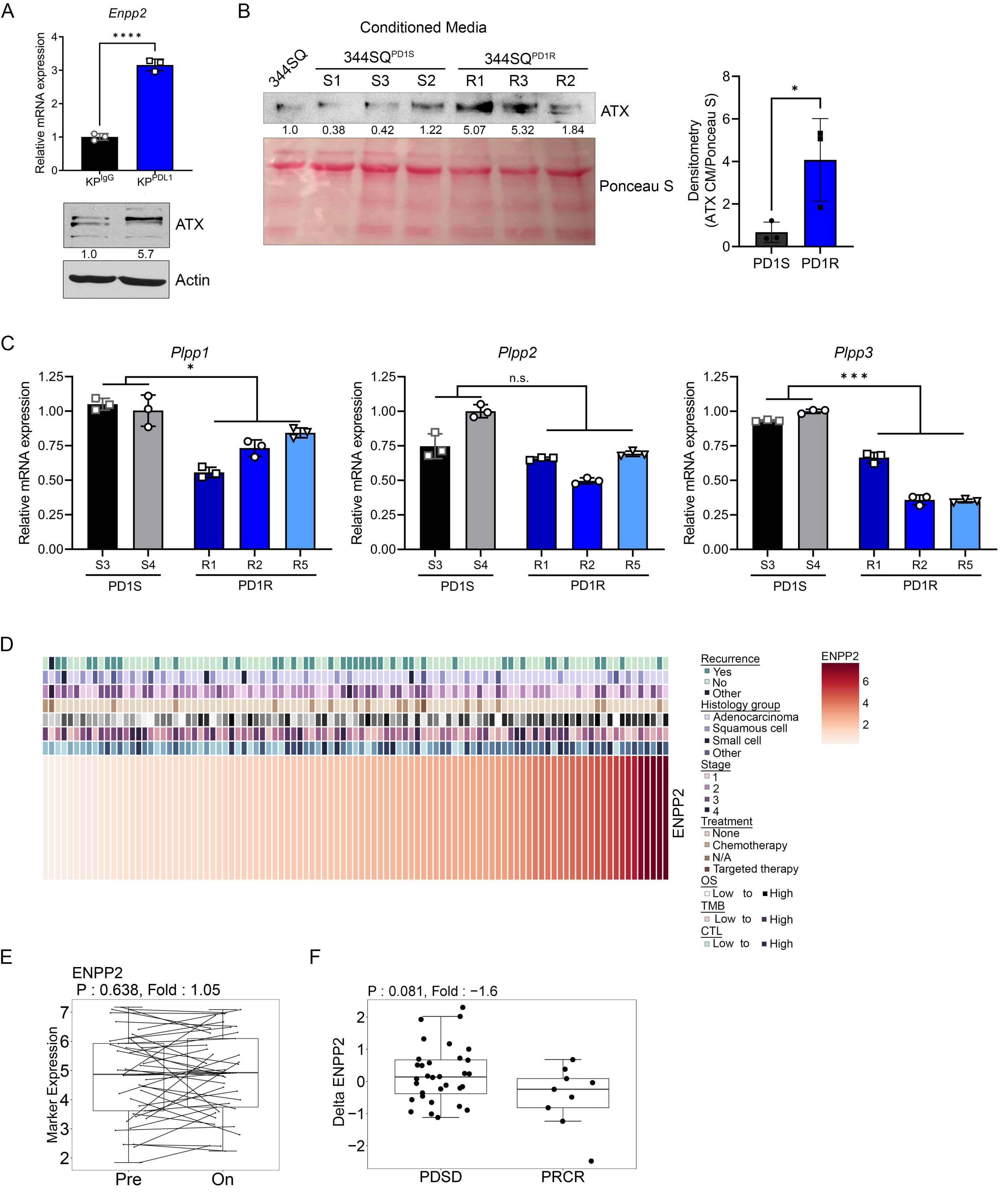

A
Enpp2
Relative mRNA expression
KP^IgG
KP^PDL1
ATX
1.0
5.7
Actin
B
Conditioned Media
344SQ
344SQ^PD1S
344SQ^PD1R
S1 S3 S2 R1 R3 R2
ATX
1.0 0.38 0.42 1.22 5.07 5.32 1.84
Ponceau S
Densitometry (ATX CM/Ponceau S)
PD1S
PD1R
C
Plpp1
Plpp2
Plpp3
n.s.
Relative mRNA expression
S3 S4 R1 R2 R5
PD1S
PD1R
D
Recurrence
Yes
No
Other
Histology group
Adenocarcinoma
Squamous cell
Small cell
Other
Stage
1
2
3
4
Treatment
None
Chemotherapy
N/A
Targeted therapy
OS
Low to High
TMB
Low to High
CTL
Low to High
ENPP2
E
ENPP2
P : 0.638, Fold : 1.05
Marker Expression
Pre
On
F
P : 0.081, Fold : −1.6
Delta ENPP2
PDSD
PRCR

Fig S4. Increased Enpp2/ATX is associated with activated immune signature and anti-PD-1 resistance in mouse and human tumors. A. The KP^{IgG} and KP^{PDL1} cell lines were collected for RNA and protein to analyze *Enpp2*/ATX expression via qPCR (top) and western blotting (bottom). ***p<0.001 by *t* test. **B.** 344SQ parental, anti-PD-1 sensitive, and anti-PD-1 resistant conditioned media was collected for western blot analysis of secreted ATX expression. Ponceau S stain shows total protein levels and acts as a loading control. Densitometry was performed by measuring the ATX band compared to the Ponceau S stain and all values were normalized to the 344SQ parental line. This densitometry was quantified in the graph to the right. *p<0.05 by *t* test. **C.** $344SQ^{PD1S}$ and $344SQ^{PD1R}$ tumors were collected for RNA and the expression of *Plpp1-3* were analyzed via qPCR. L32 was used as an internal control, and expression levels in all samples were normalized to the S4 sample. *p<0.05, **p<0.01, ***p<0.001 by *t* tests with grouped samples (PD1S versus PD1R). **D.** MD Anderson ICON patients were analyzed for expression of *ENPP2* correlated to numerous clinical features, including overall survival (OS), T cell cytolytic score (CYT), histology, treatment, tumor mutational burden (TMB). **E-F.** Analysis of the published dataset from melanoma patients on nivolumab therapy (GSE91061)(34). Individual paired tumors (pre-treatment and on-treatment) were analyzed for ENPP2 expression levels. **F.** The change in *ENPP2* expression (delta) between pre-treatment and on-treatment samples was calculated. Patients were separated into progressive/stable disease (PDSD) or partial/complete responders (PRCR), and the delta in ENPP2 expression was graphed in each of these patient categories.

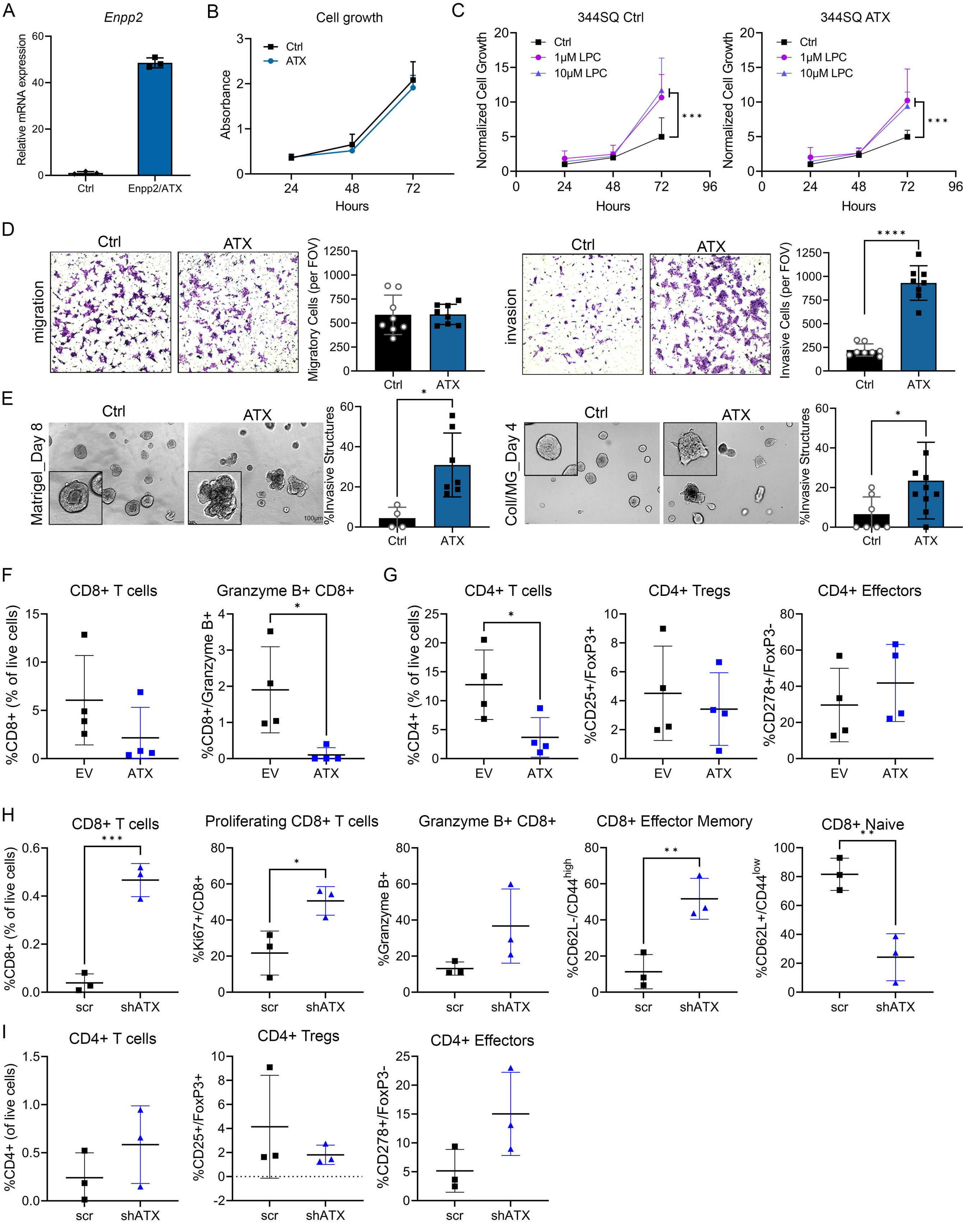

A
Enpp2
Relative mRNA expression
Ctrl
Enpp2/ATX
B
Cell growth
Absorbance
Ctrl
ATX
Hours
C
344SQ Ctrl
344SQ ATX
Normalized Cell Growth
Ctrl
1μM LPC
10μM LPC
Hours
D
Ctrl
ATX
migration
Migratory Cells (per FOV)
invasion
Invasive Cells (per FOV)
E
Matrigel_Day 8
Coll/MG_Day 4
%Invasive Structures
100μm
F
CD8+ T cells
%CD8+ (% of live cells)
Granzyme B+ CD8+
%CD8+/Granzyme B+
EV
ATX
G
CD4+ T cells
%CD4+ (% of live cells)
CD4+ Tregs
%CD25+/FoxP3+
CD4+ Effectors
%CD278+/FoxP3-
H
CD8+ T cells
%CD8+ (% of live cells)
Proliferating CD8+ T cells
%Ki67+/CD8+
Granzyme B+ CD8+
%Granzyme B+
CD8+ Effector Memory
%CD62L-/CD44high
CD8+ Naive
%CD62L+/CD44low
scr
shATX
I
CD4+ T cells
%CD4+ (of live cells)
CD4+ Tregs
%CD25+/FoxP3+
CD4+ Effectors
%CD278+/FoxP3-

Fig S5. ATX expression promotes invasion of 344SQ lung cancer cells and decreases CD8+ T cell phenotypes in tumors. A. 344SQ vector (ctrl) or ATX-overexpressing cells were analyzed via qPCR to confirm *Enpp2* levels. L32 was used as an internal control, and values are normalized to control cells. **B.** 344SQ ctrl and ATX-overexpressing cells were plated for a WST-1 proliferation assay over 3 days. Raw absorbance values are graphed. **C**. 344SQ ctrl and ATX cells were plated for a WST-1 assay as described in B. At the time of plating, DMSO control or lysophosphatidylcholine (LPC) was added to cells. Absorbance values are graphed relative to the 24hr no LPC control **** $p<0.0001$ by 2-way ANOVA with Tukey's correction. **D.** The migratory and invasive capabilities of the 344SQ ctrl and ATX cells were analyzed using Boyden Transwell assays without Matrigel (migration; left) or with Matrigel (invasion; right). n= 3 chambers/cell line. **E.** 344SQ ctrl and ATX overexpressing cells were plated in 3-D assays using either 100% Matrigel (left) or a 50:50 mixed Matrigel/Collagen type I matrix (right). Representative brightfield images are shown in each condition. Invasive structures were calculated as a percentage of total structures. n = 2-3 wells/condition, 5 images/well. *$p<0.05$ by *t* test. **F.** 344SQ ctrl and ATX-overexpressing cells were implanted into mice. After 3 weeks, tumors were excised, and immune populations were characterized by flow cytometry. Total CD8+ T cells were calculated as a fraction of live cells, and Granzyme B+ cells were calculated underneath the CD8+ population. *$p<0.05$ by *t* test. **G.** The tumors from F were analyzed for the CD4+ subpopulations. **H.** The $344SQ^{PD1R2}$-shATX#4 and scrambled control cells (described in Figure 4) were implanted into mice and after 3 weeks, tumors were excised and processed for flow cytometry as described in Figure 2. *$p<0.05$, **$p<0.01$ by *t* test. **I.** The tumors from H were analyzed for CD4+ subpopulations by flow cytometry.

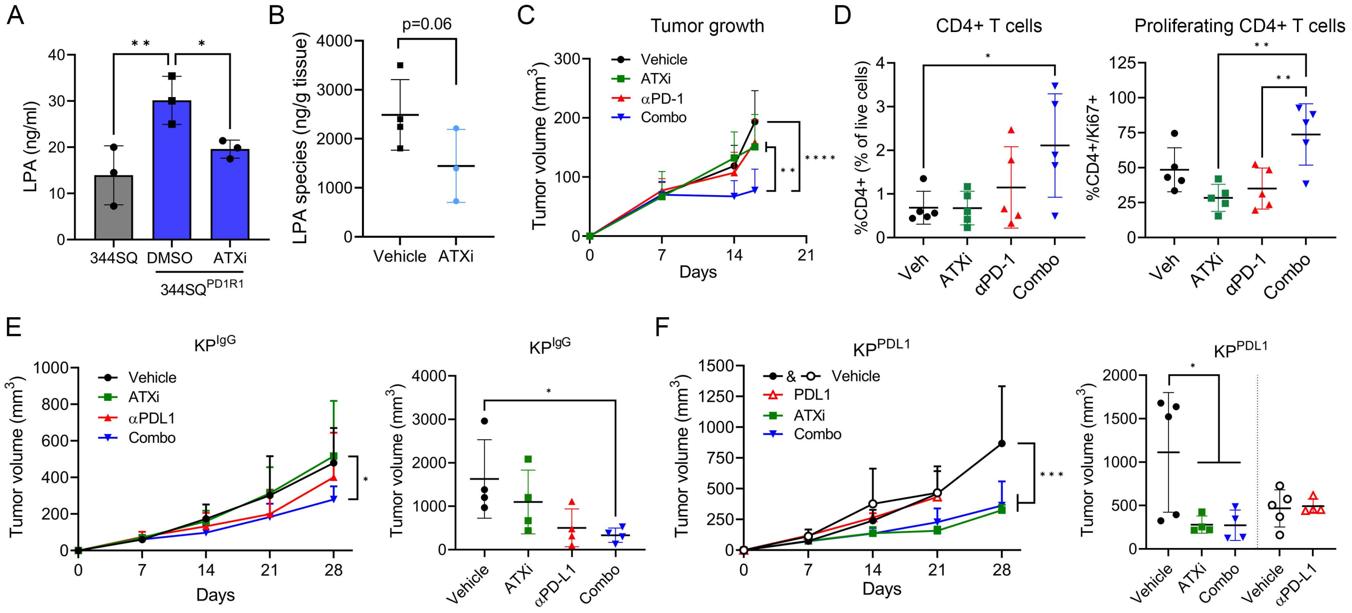

A
LPA (ng/ml)
344SQ
DMSO
ATXi
344SQPD1R1
B
p=0.06
LPA species (ng/g tissue)
Vehicle
ATXi
C
Tumor growth
Tumor volume (mm3)
Vehicle
ATXi
αPD-1
Combo
Days
D
CD4+ T cells
%CD4+ (% of live cells)
Proliferating CD4+ T cells
%CD4+/Ki67+
Veh
ATXi
αPD-1
Combo
E
KPIgG
Tumor volume (mm3)
Vehicle
ATXi
αPDL1
Combo
Days
KPIgG
Vehicle
ATXi
αPD-L1
Combo
F
KPPDL1
& Vehicle
PDL1
ATXi
Combo
Days
KPPDL1
Vehicle
ATXi
Combo
Vehicle
αPD-L1

Fig S6. ATX inhibition with anti-PD-1 increased CD4+ proliferation and efficaciously controlled tumor growth in KP anti-PD-1 sensitive and resistant tumor models. A. 344SQ and $344SQ^{PD1R1}$ cells were plated for an LPA ELISA. To the $344SQ^{PD1R1}$ cells, the ATX inhibitor PF-8380 was added at 1μM for 24 hours prior to media collection for ELISA. *p<0.05, **p<0.01 by one-way ANOVA corrected for multiple comparisons. **B.** 344SQ tumors collected from mice treated with the PF-8380 ATX inhibitor or vehicle control (from the experiment in Figure 5C) were processed for LPA ELISA. **C.** Tumor growth data for flow experiment described in Figure 5A. **p<0.01, ****p<0.0001 by 2-way ANOVA. **D.** Additional flow analysis from the experiment shown in Figure 5A. Total CD4+ T cells were gated under total T cells (CD45+/CD3+) (left). Proliferating CD4+ T cells were gated under total CD4+ T cells and were Ki67+ (right). **p<0.01 by one-way ANOVA corrected for multiple comparisons. **E.** The KP^{IgG} cell line (anti-PD-L1 sensitive) was implanted into wildtype mice and treated with vehicle control, the ATX inhibitor PF-8380, anti-PD-L1, or the combination. Tumor growth over time was measured weekly via calipers (left) and at endpoint (right). n = 4-5 mice/treatment. *p<0.05 by 1-way (right graph) or 2-way ANOVA (left graph). **F.** The KP^{PDL1} cell line (anti-PD-L1 resistant) was implanted into wildtype mice. Mice were treated and tumor growth measured as described in D. Open shapes are from one experiment that lasted 3 weeks, and closed shapes are from another experiment that went for 4 weeks. n = 4-5mice/treatment. ***p<0.001 by 2-way ANOVA (left graph), *p<0.05 by 1-way ANOVA (right graph).

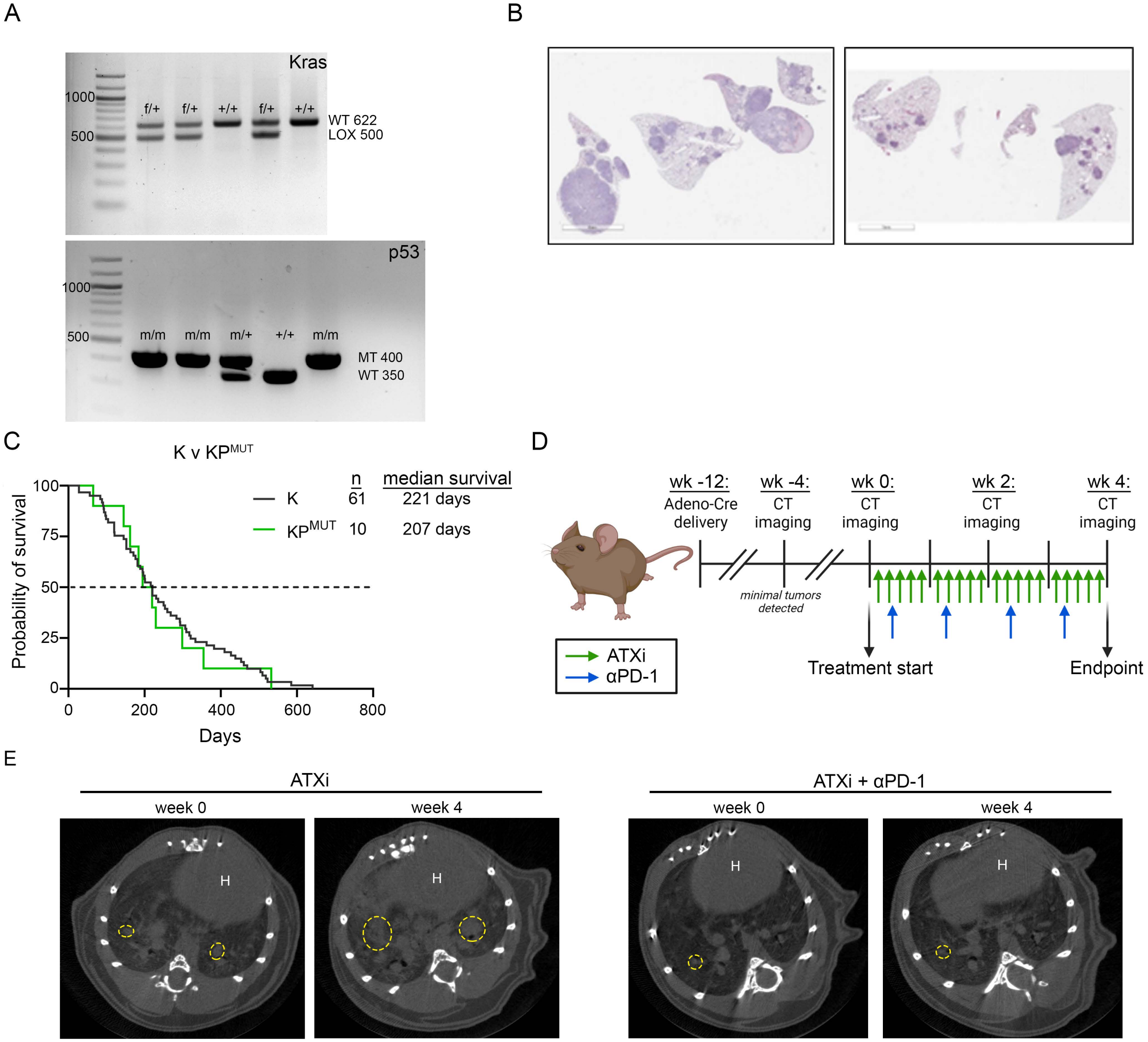

A
Kras
1000
500
f/+
f/+
+/+
f/+
+/+
WT 622
LOX 500
p53
1000
500
m/m
m/m
m/+
+/+
m/m
MT 400
WT 350
B
C
K v KP^MUT
Probability of survival
100
75
50
25
0
0
200
400
600
800
Days
n
median survival
K
61
221 days
KP^MUT
10
207 days
D
wk -12:
Adeno-Cre delivery
wk -4:
CT imaging
wk 0:
CT imaging
wk 2:
CT imaging
wk 4:
CT imaging
minimal tumors detected
Treatment start
Endpoint
ATXi
αPD-1
E
ATXi
ATXi + αPD-1
week 0
week 4
week 0
week 4
H

Fig S7. $Kras^{LSL\text{-}G12D}/p53^{wm\text{-}R172H}$ mutant mice develop primary lung adenocarcinoma after delivery of intratracheal Cre-recombinase. A. Genomic DNA was collected from ear clips from $Kras^{LSL\text{-}G12D}/p53^{wm\text{-}R172H}$ (KP), and genotyping for the mice to confirm mutational status, ran on 2% agarose gel. The genotyping for the status of the Kras allele is shown in top panel. Wildtype (WT) bands are 622bp in size and the LOX bands are 500bp (indicating the insertion of Lox-Stop-Lox cassette). From left to right, these mice are heterozygous (Het), Het, WT, Het, and WT. The genotyping for the status of the Trp53 allele is shown in the bottom panel. The mutant allele is shown at 400bp and the WT allele at 350bp. From left to right, these mice homozygous mutant (Hom), Hom, Het, WT, Hom. **B.** Microscopy images of H&E staining of $KRas^{LSL\text{-}G12D}/p53^{wm\text{-}R172H}$ mice. Mice were sacrificed and necropsied at humane endpoints when their survival was no longer assured. Left image: 162 days, Right: 195 days post-viral induction. **C.** Kaplan-Meier survival curves of Kras vs KP mice. All KP mice are homozygous for p53. **D.** Schematic illustrating the GEMM treatment experiment. KP conditional mice were induced with adenoviral Cre-recombinase and micro-CT imaging began 8 weeks later (week -4). After another 4 weeks to allow more tumor development, mice were imaged again as the pre-treatment measurement (week 0), randomized into either ATX inhibitor alone or combination ATX inhibitor plus anti-PD-1, and treatment started. This treatment regime continued for 4 weeks, with one on-treatment imaging session (week 2) and an endpoint imaging session (week 4). **E.** Representative pre-treatment (week 0) and endpoint (week 4) micro-CT images from the experiment described in Figure 5G, H. Dashed yellow circles indicate lung tumors. H = heart.

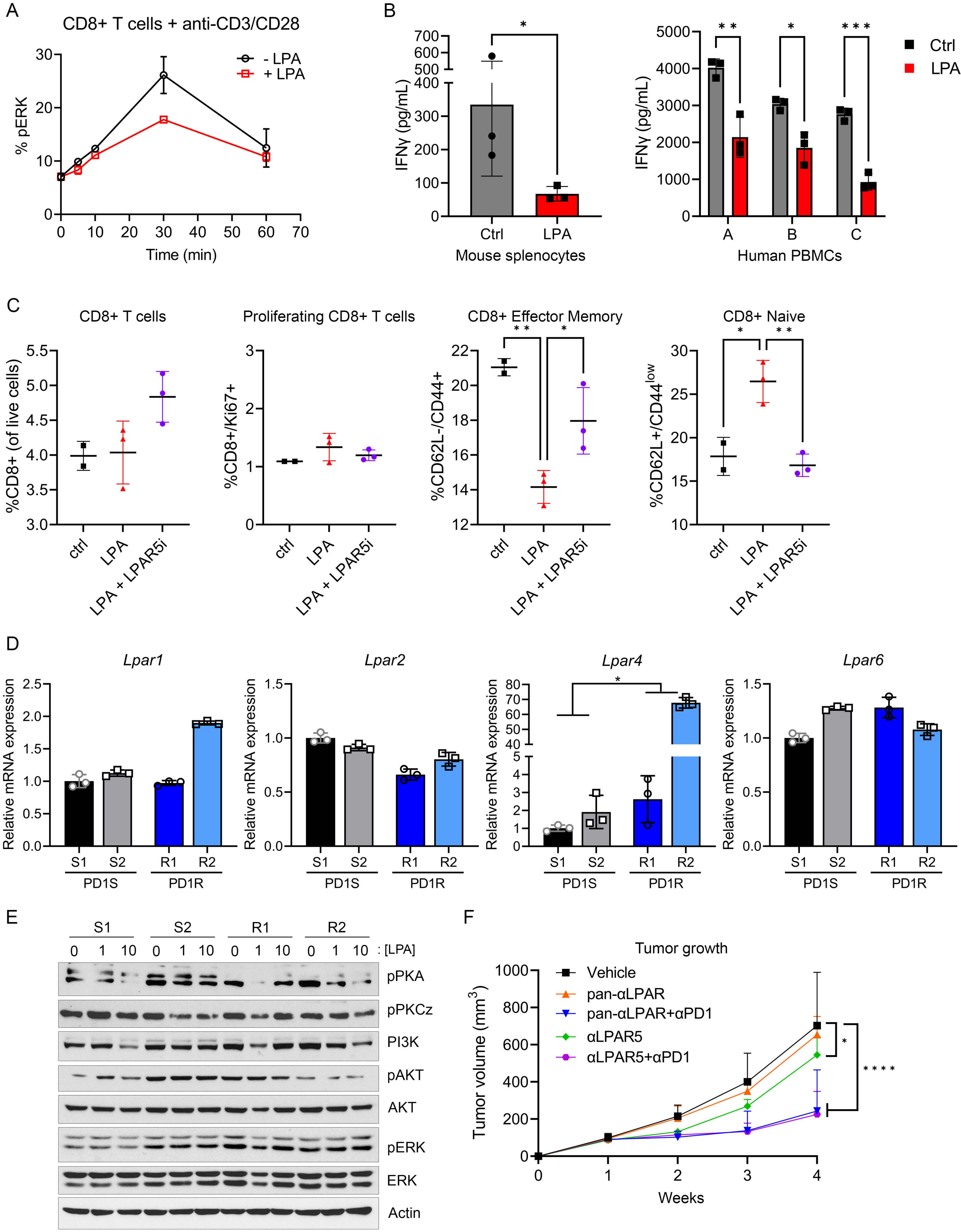

A
CD8+ T cells + anti-CD3/CD28
- LPA
+ LPA
% pERK
Time (min)
B
IFNγ (pg/mL)
Ctrl
LPA
Mouse splenocytes
Human PBMCs
C
CD8+ T cells
Proliferating CD8+ T cells
CD8+ Effector Memory
CD8+ Naive
%CD8+ (of live cells)
%CD8+/Ki67+
%CD62L-/CD44+
%CD62L+/CD44low
ctrl
LPA
LPA + LPAR5i
D
Lpar1
Lpar2
Lpar4
Lpar6
Relative mRNA expression
S1
S2
R1
R2
PD1S
PD1R
E
: [LPA]
pPKA
pPKCz
PI3K
pAKT
AKT
pERK
ERK
Actin
F
Tumor growth
Vehicle
pan-αLPAR
pan-αLPAR+αPD1
αLPAR5
αLPAR5+αPD1
Tumor volume (mm3)
Weeks

Fig S8. LPA diminishes CD8+ T cell functions. A. Naïve CD8+ T cells were purified from murine splenocytes and activated using anti-CD3 and anti-CD28 antibodies, alone or with treatment of exogenous lysophosphatidic acid (LPA). T cells were then collected at various timepoints of stimulation and stained for flow cytometry analysis of phospho-ERK levels. n = 2 replicates/condition. **B.** Naïve immune cells from murine splenocytes (left) or human PBMCs from healthy donors (right) were stimulated with anti-CD3 and anti-CD28 in the presence of LPA (20 μM) or solvent control for 24 hours. Conditioned media was then collected for IFN-γ ELISA analysis. *p<0.05, **p<0.01, ***p<0.001 by multiple *t* tests. **C.** Naïve immune cells isolated from mouse splenocytes were stimulated with anti-CD3 and anti-CD28 in the presence of LPA (20 μM) and/or the LPAR5 inhibitor AS2717638 (5 μM). After 72 hours, immune cells were collected and stained for multicolor flow cytometry analysis. *p<0.05, **p<0.01 by 1-way ANOVA. **D.** 344SQ anti-PD-1 sensitive (S1 and S2) and anti-PD-1 resistant (R1 and R2) were analyzed via qPCR for expression of Lpar1, Lpar2, Lpar3 (no amplification; not shown), Lpar4, and Lpar6. L32 was used as an internal normalization control, and samples were all normalized to the S1 line. **E.** The cells from D were stimulated with increasing concentrations of LPA (1μM and 10μM) or control and collected for western blot analysis of downstream signaling pathways, including phospho-PKA, phospho-PKC, phospho-AKT, and phospho-ERK. Actin was used as a loading control. **F.** Mice bearing 344SQ tumors were treated with the pan-LPAR inhibitor BrP-LPA or the LPAR5 inhibitor AS2717638 alone and in combination with anti-PD-1 as described in Figure 6G. *p<0.05, ****p<0.0001 by 2-way ANOVA with multiple corrections.

Figure 1F

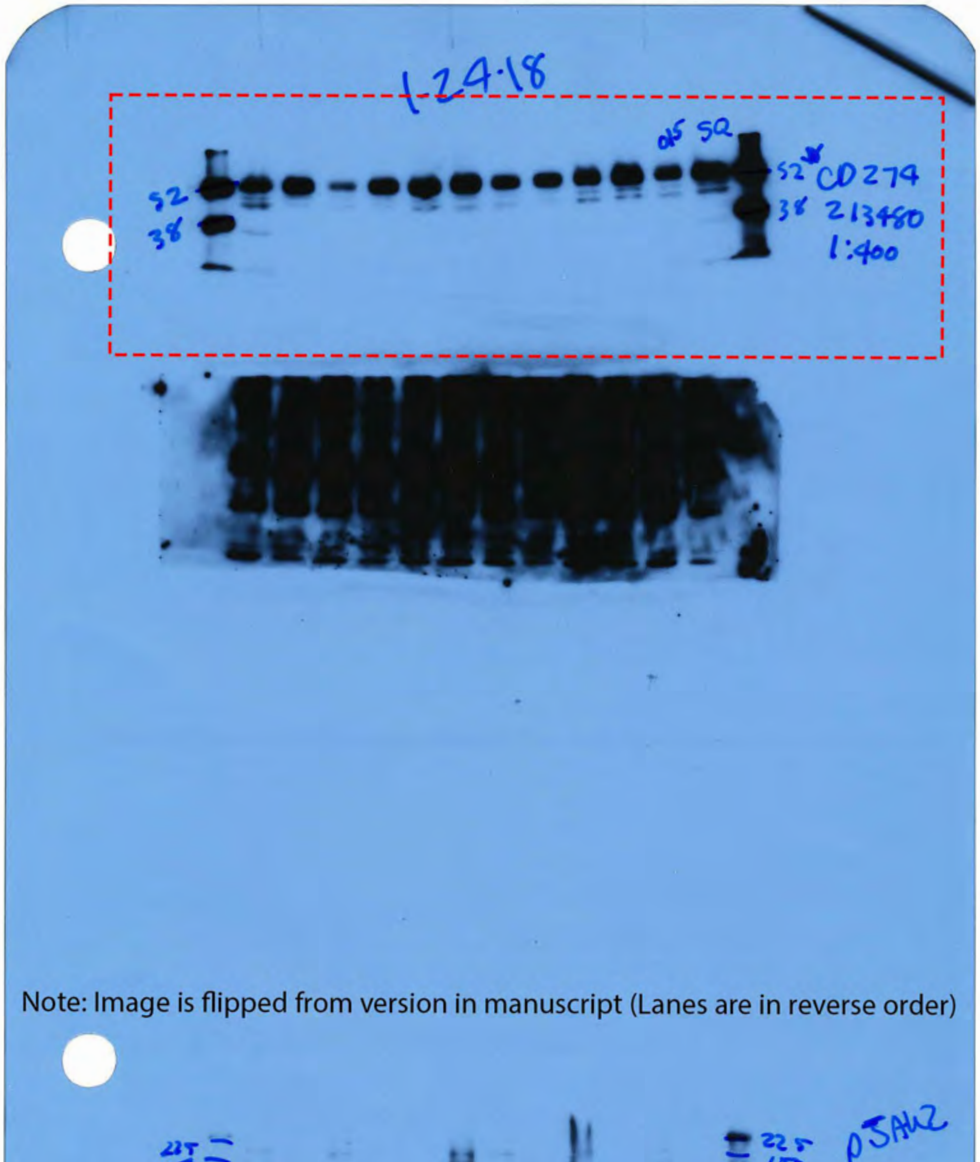

Note: Image is flipped from version in manuscript (Lanes are in reverse order)

Figure 1F

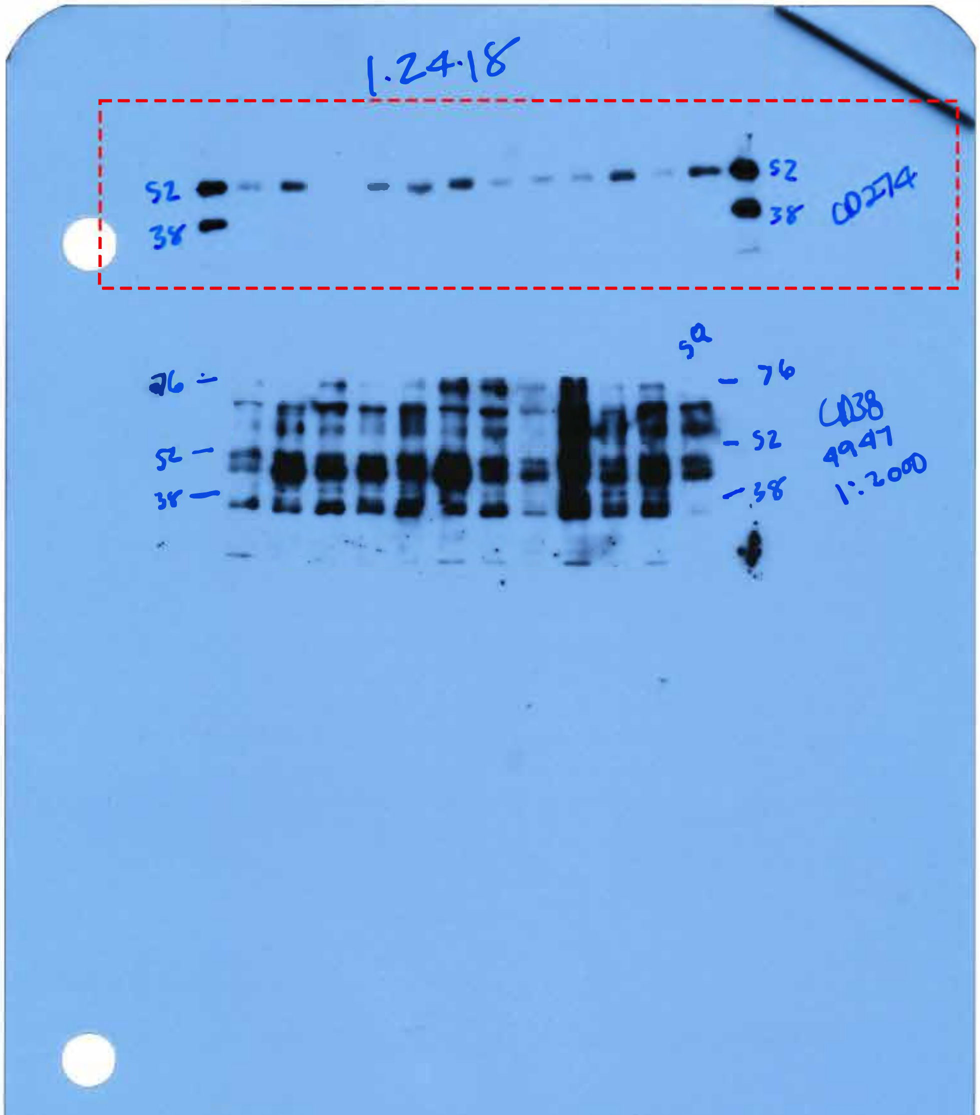

Note: Image is flipped from version in manuscript (Lanes are in reverse order)

Figure 1F

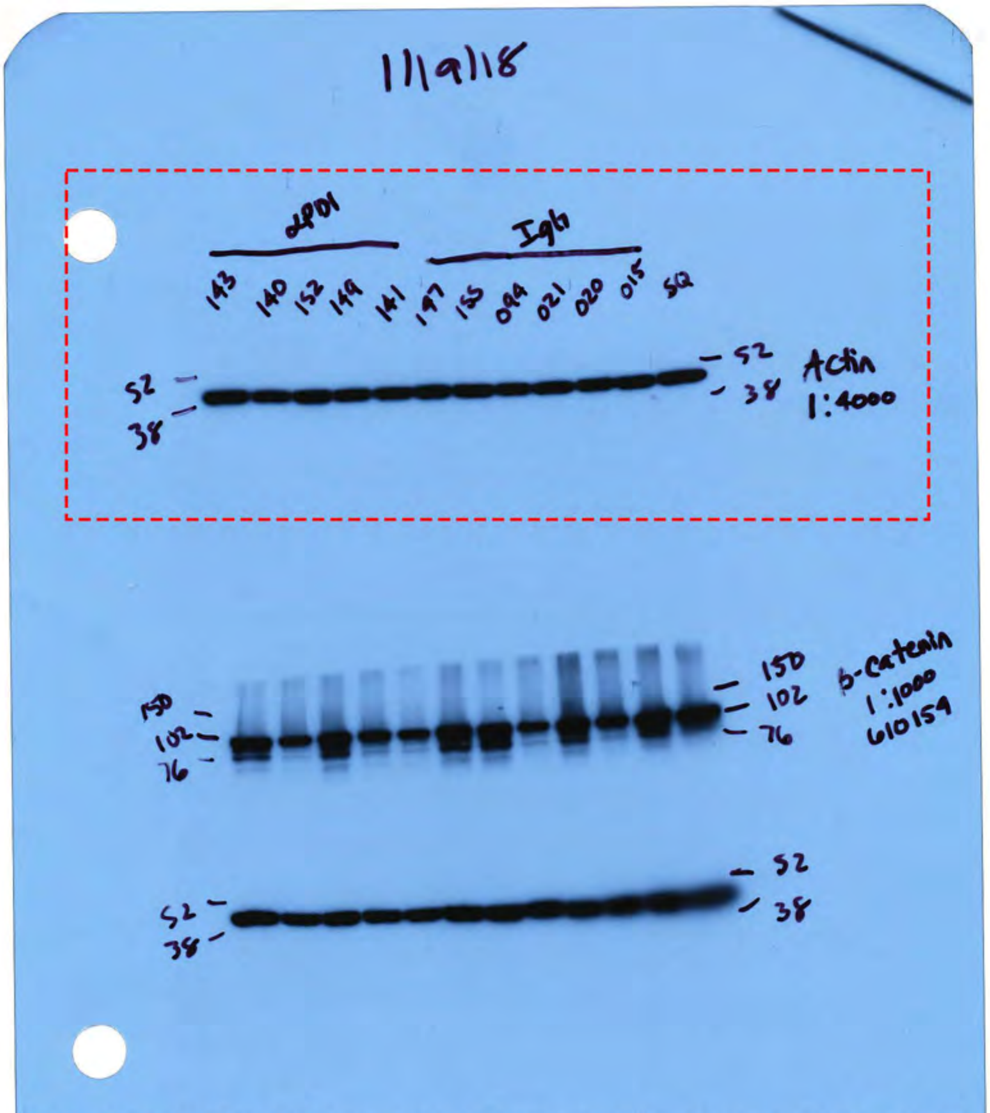

Note: Image is flipped from version in manuscript (Lanes are in reverse order)

Figure 3C

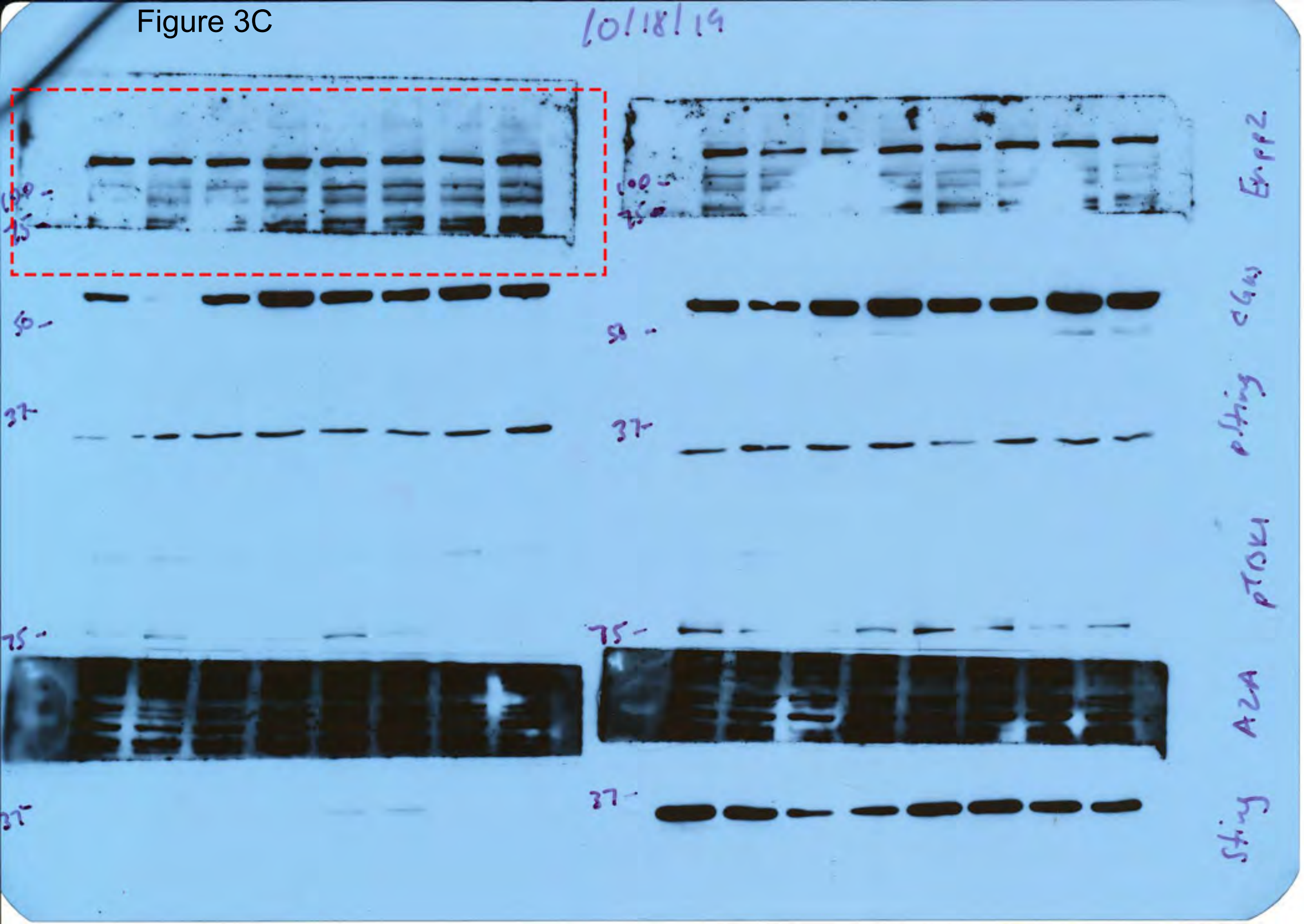

Figure 3C

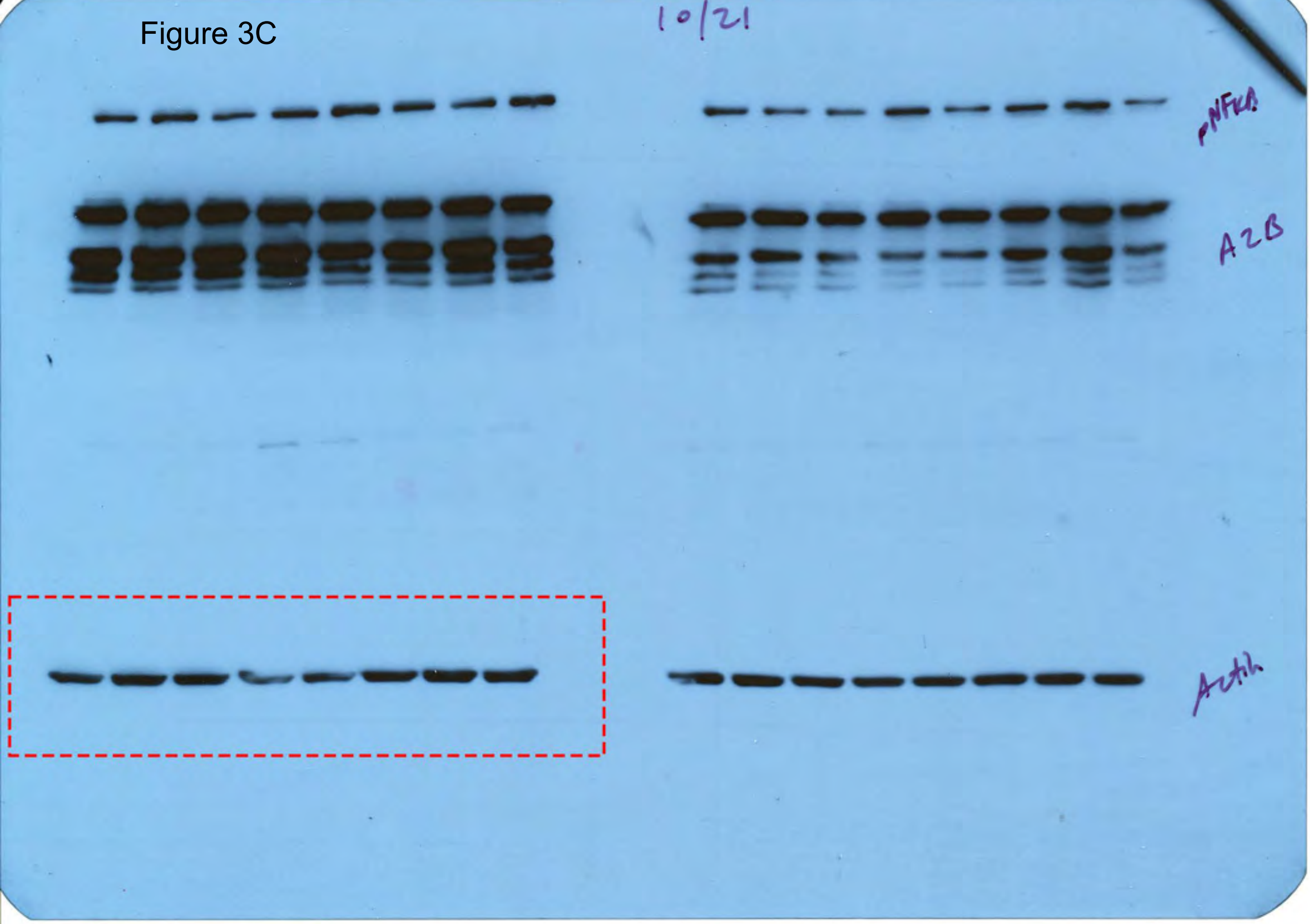

PD1S, PD1R tumors

Figure 3C

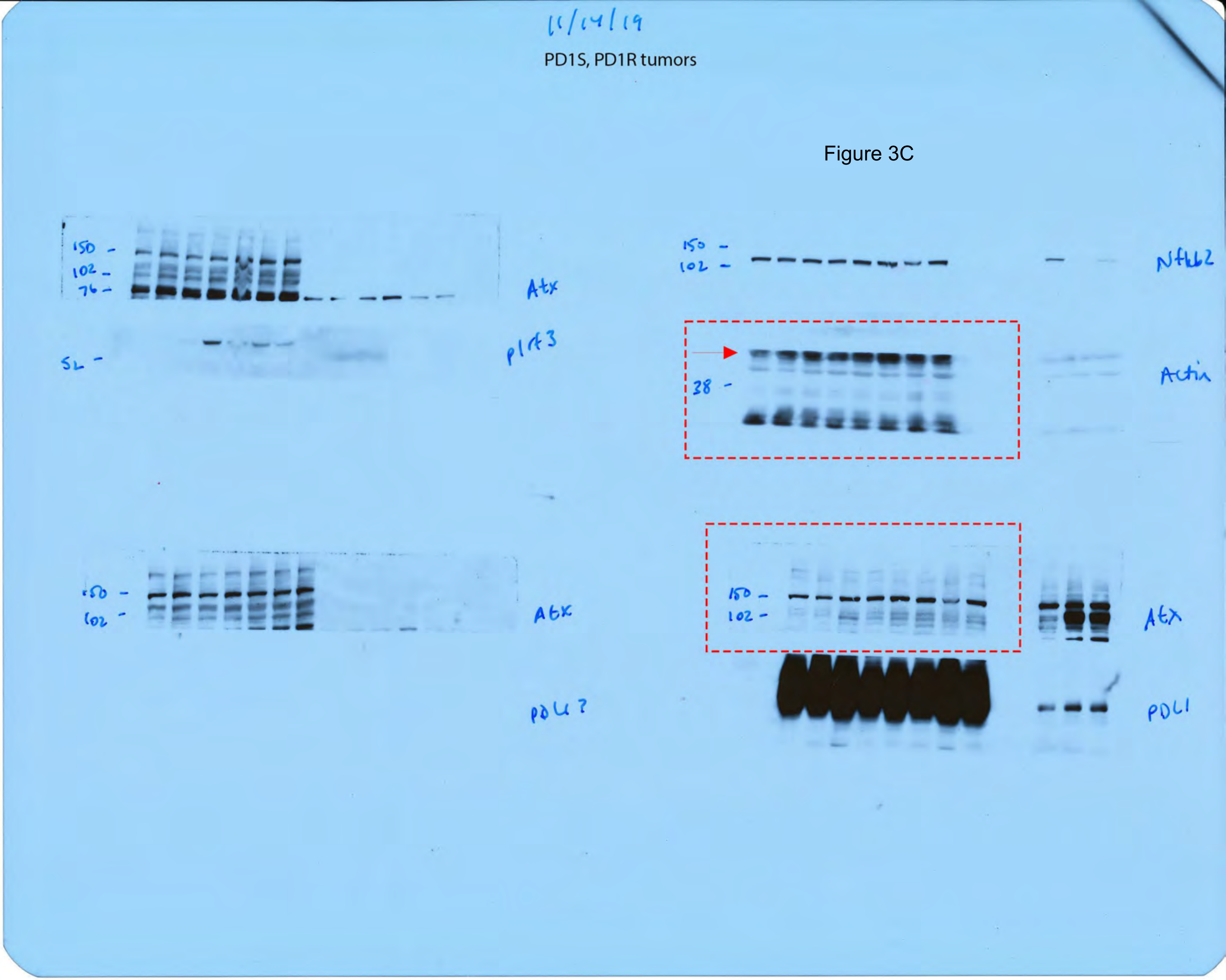

Figure 4A

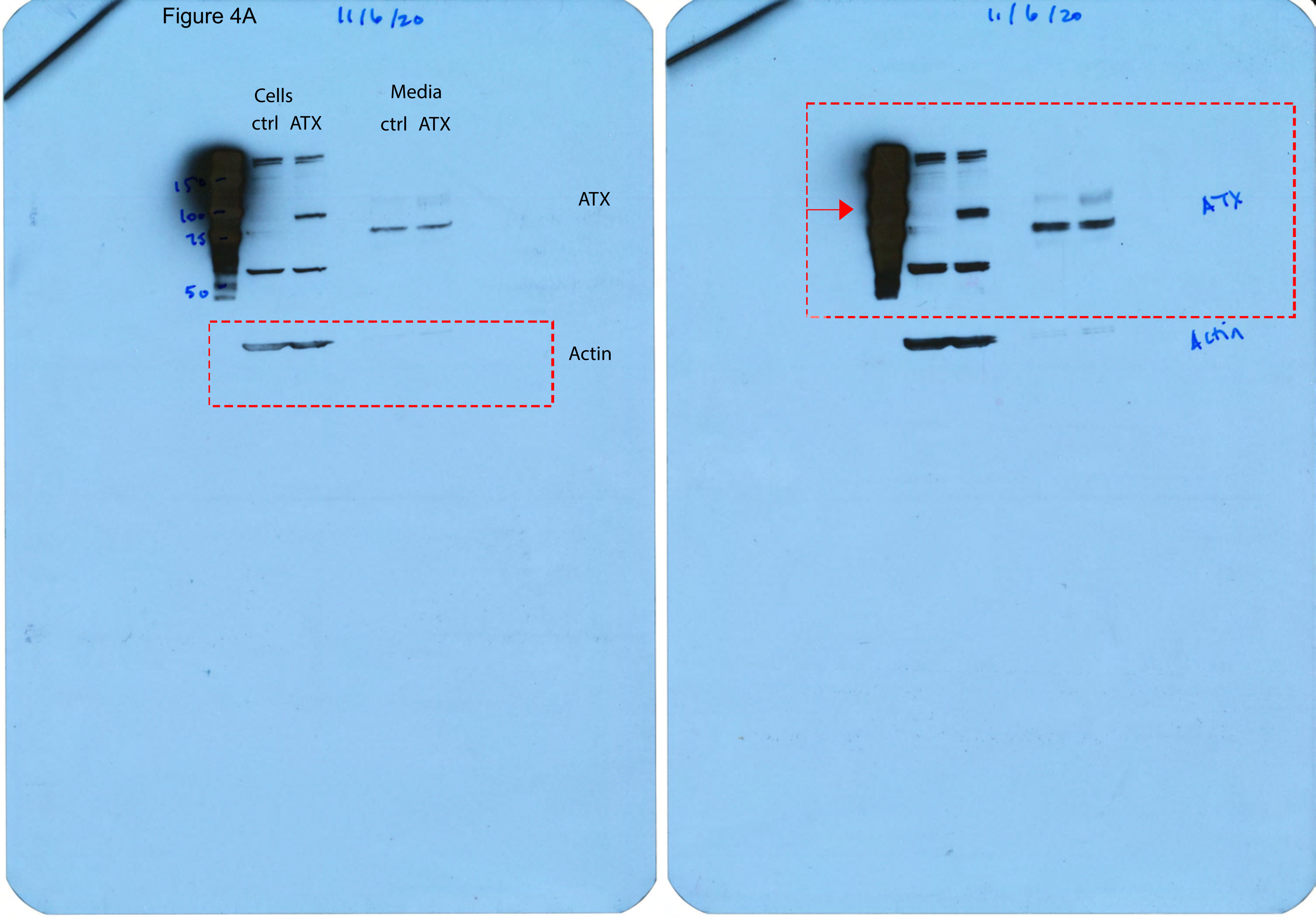
11/6/20
Cells
ctrl ATX
Media
ctrl ATX
150
100
75
50
ATX
Actin
11/6/20
ATX
Actin

Figure 4E

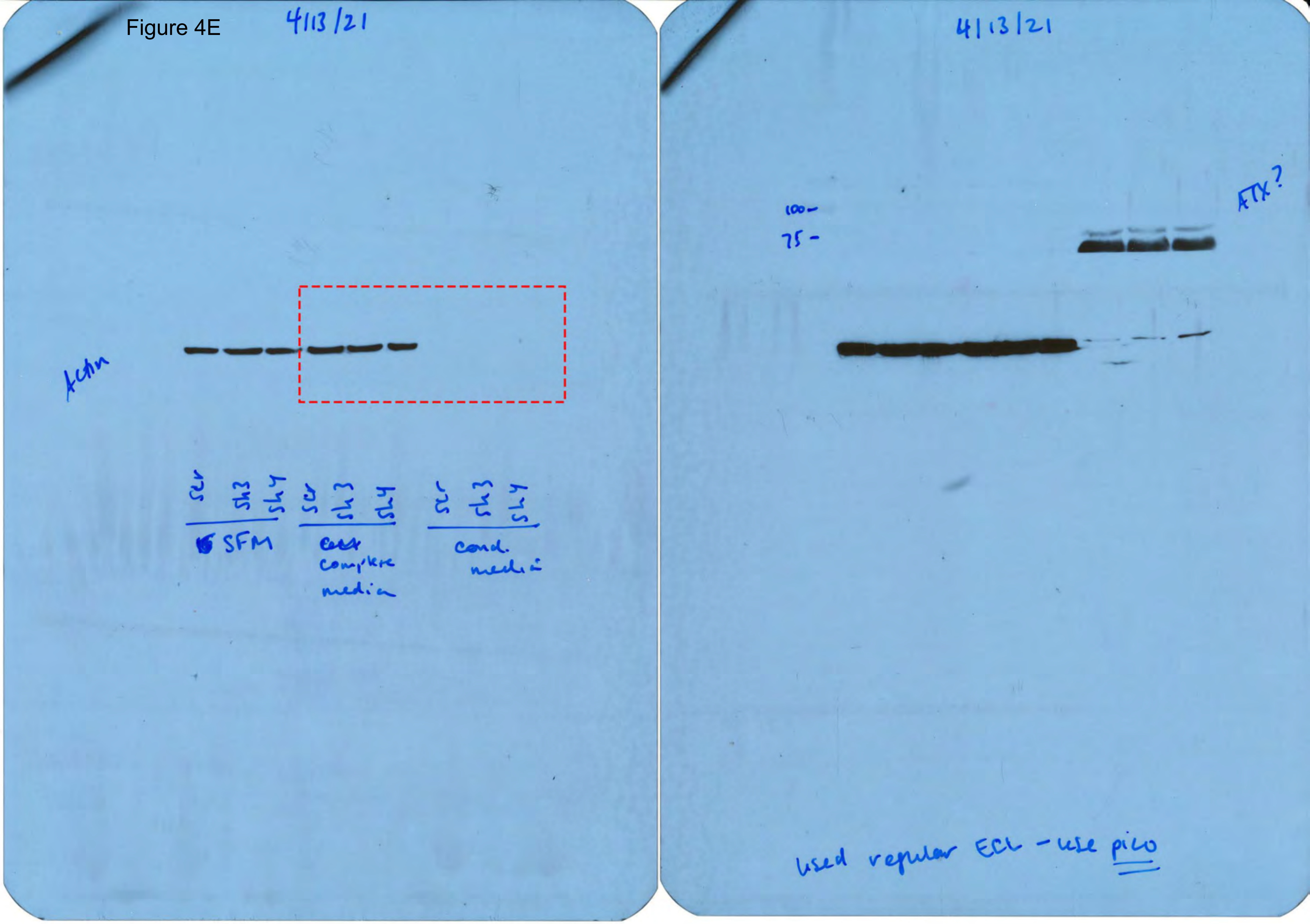
4/13/21
Actin
scr
sh3
sh4
scr
sh3
sh4
scr
sh3
sh4
SFM
complete media
cond. media
4/13/21
100-
75-
ATX?
used regular ECL - use pico

Figure 4E

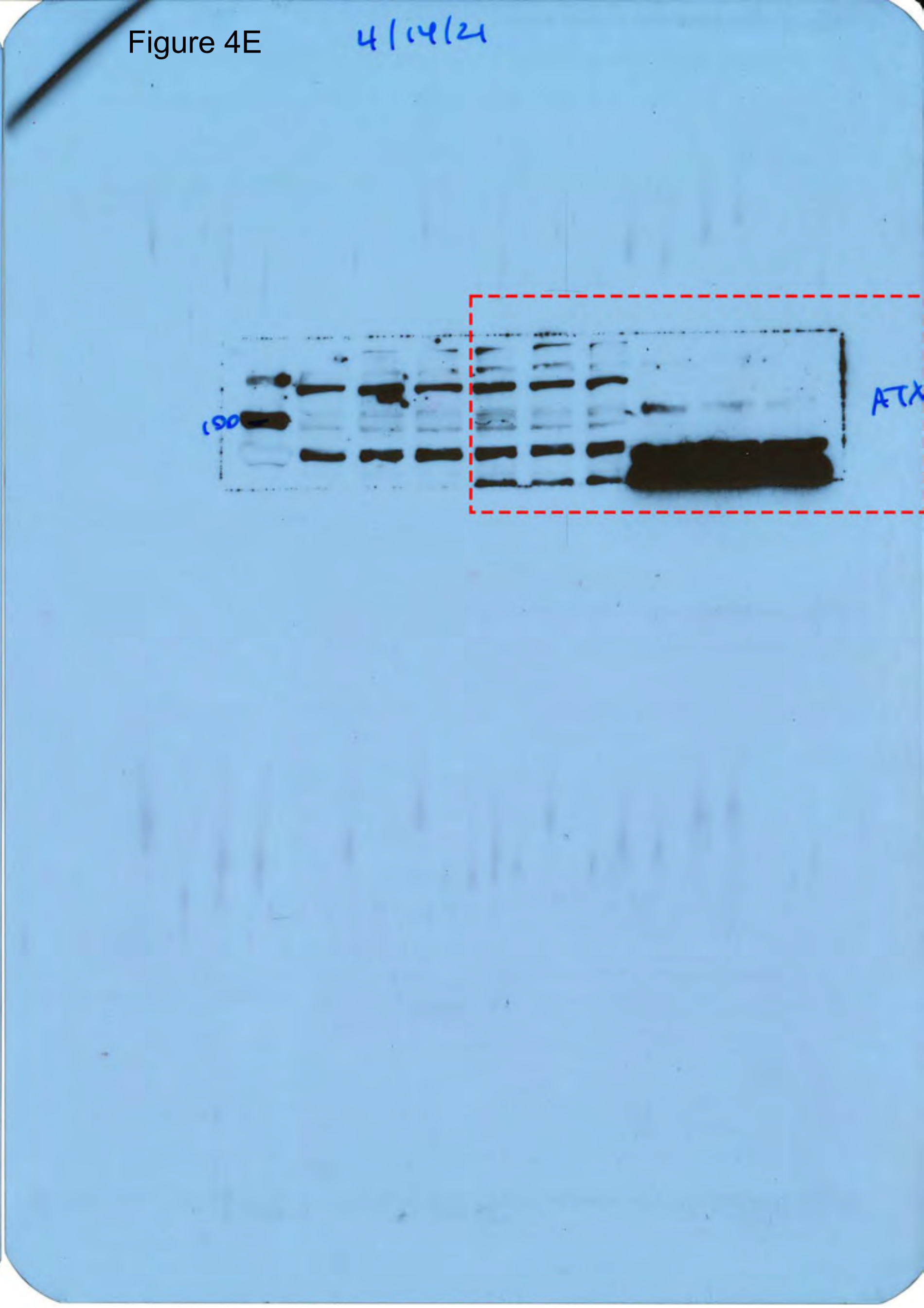

Figure S1C

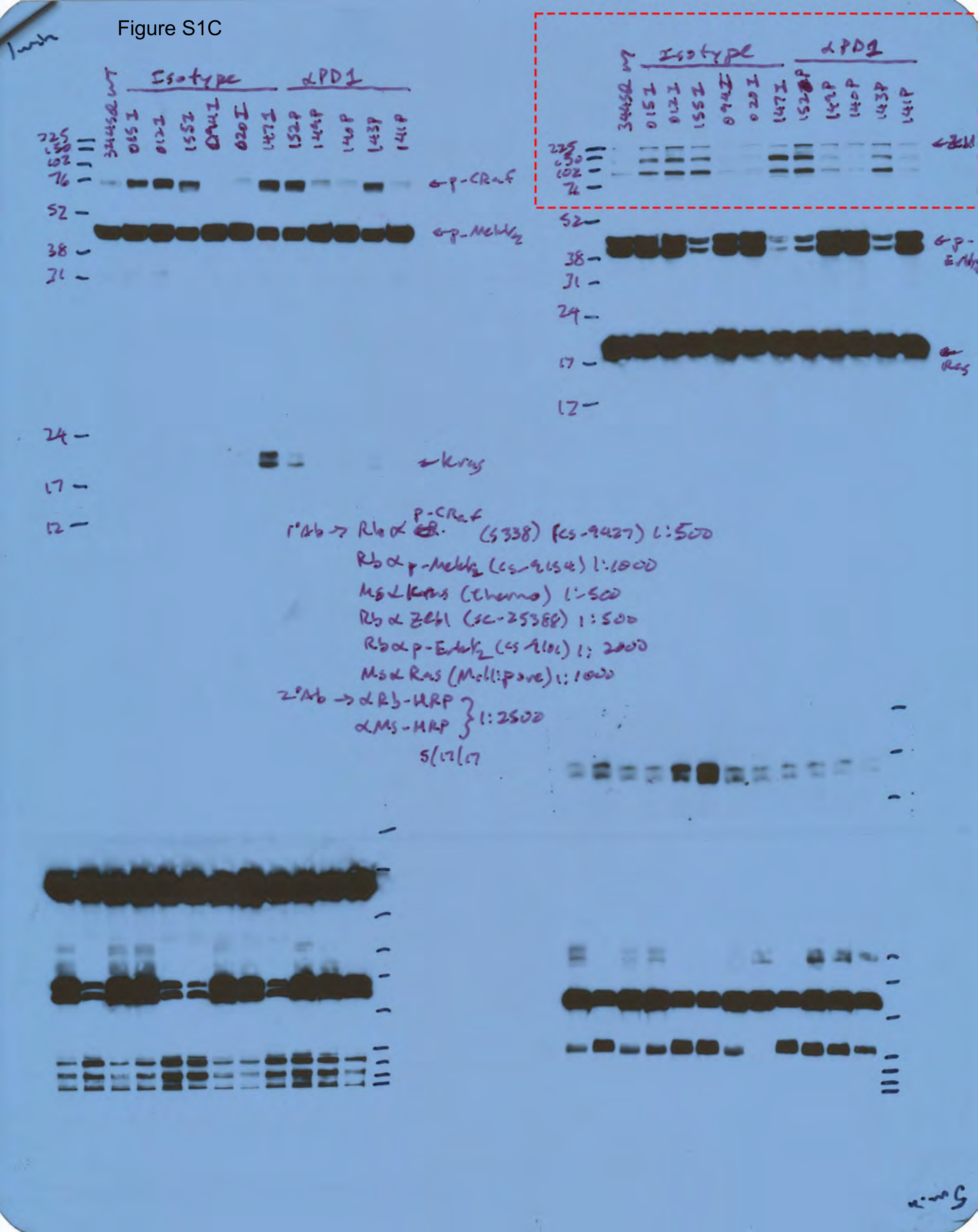
Isotype
αPD1
p-CRaf
p-Mek1/2
Kras
Zeb1
Ras
1°Ab → Rb α p-CRaf (S338) (CS-9427) 1:500
Rb α p-Mek1/2 (CS-9154) 1:1000
Ms α Kras (thermo) 1:500
Rb α Zeb1 (sc-25388) 1:500
Rb α p-Erk1/2 (CS 9101) 1:2000
Ms α Ras (Millipore) 1:1000
2°Ab → αRb-HRP
αMs-HRP
1:2500
5/17/17

Figure S1C

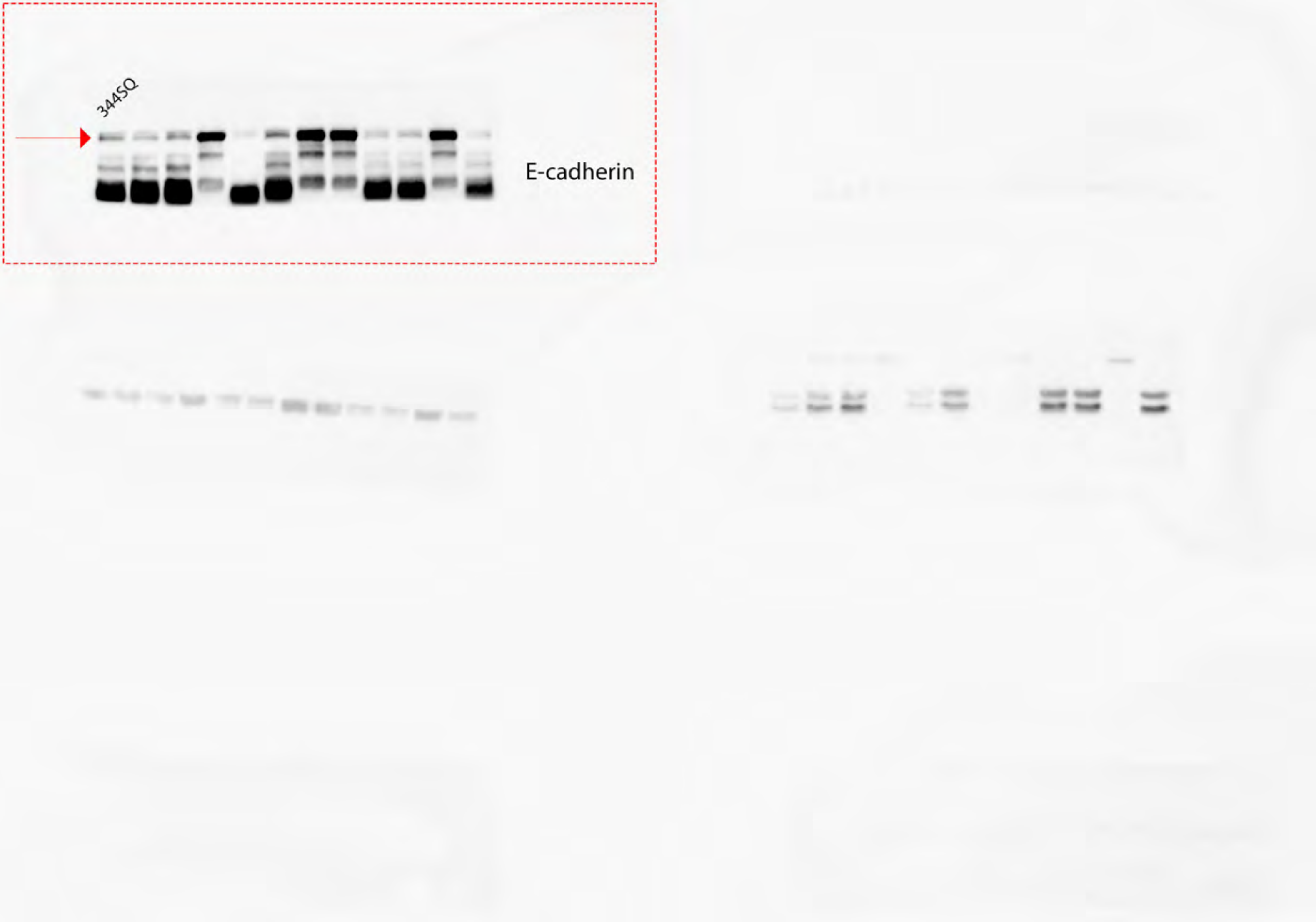

Figure S1C

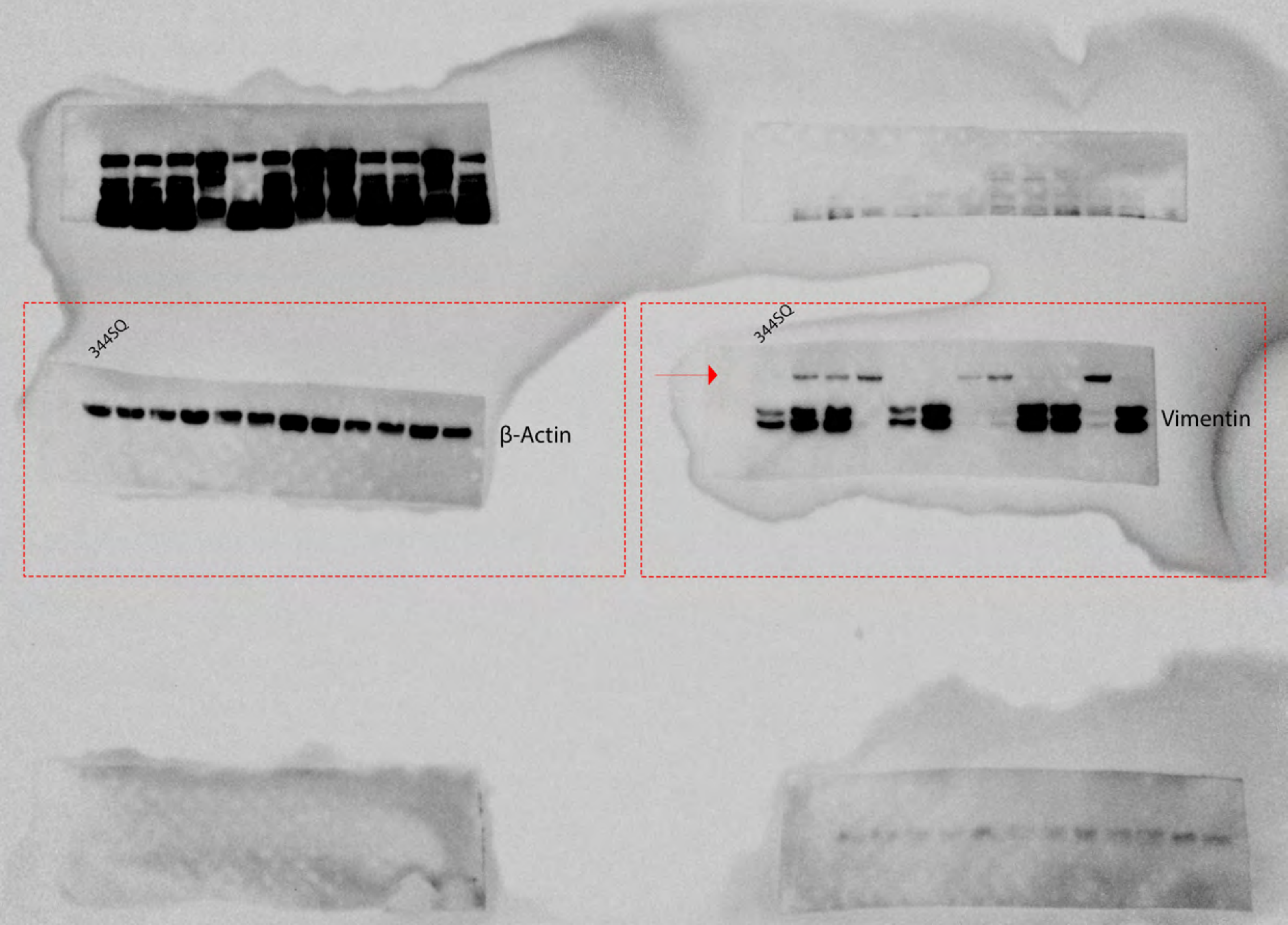

Figure S1C

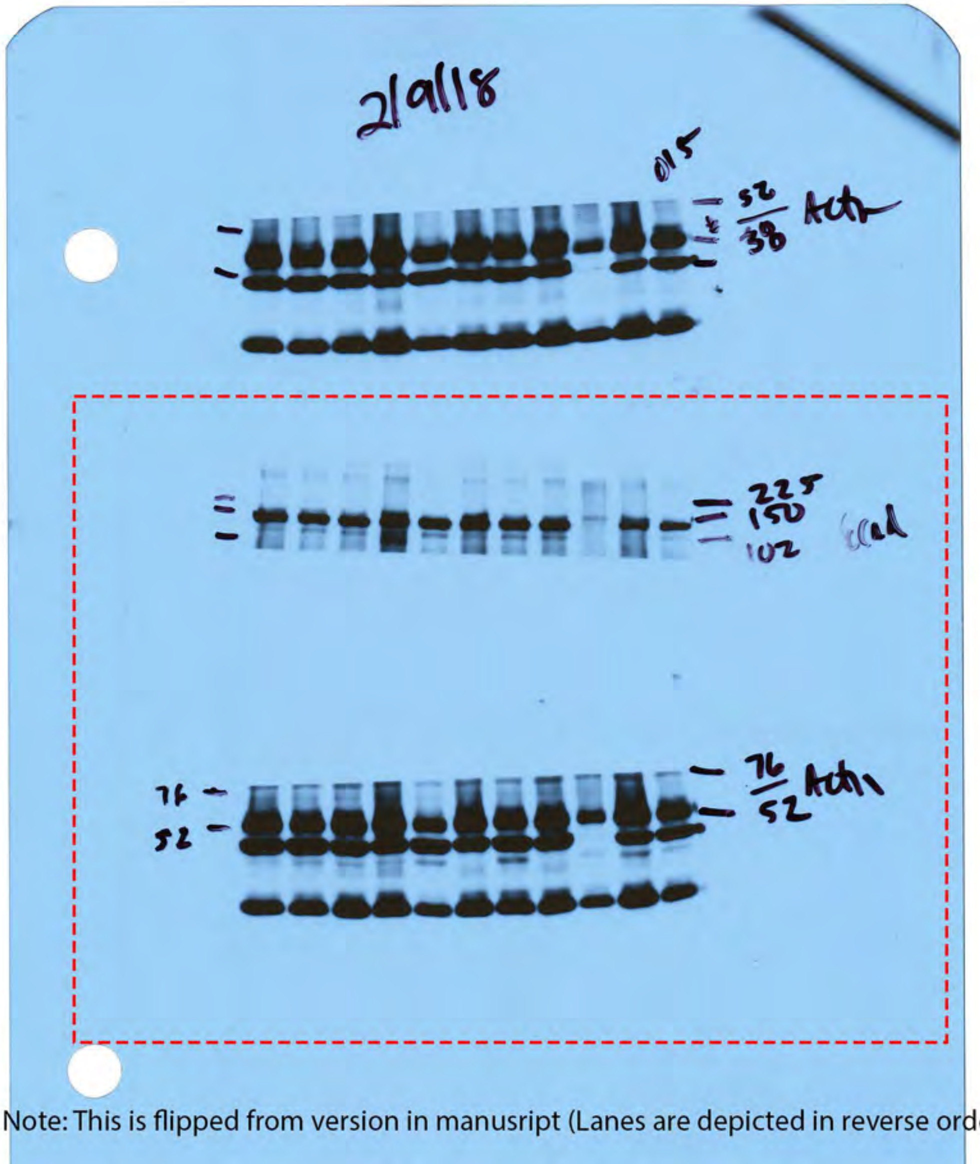

Note: This is flipped from version in manusript (Lanes are depicted in reverse order)

Figure S1C

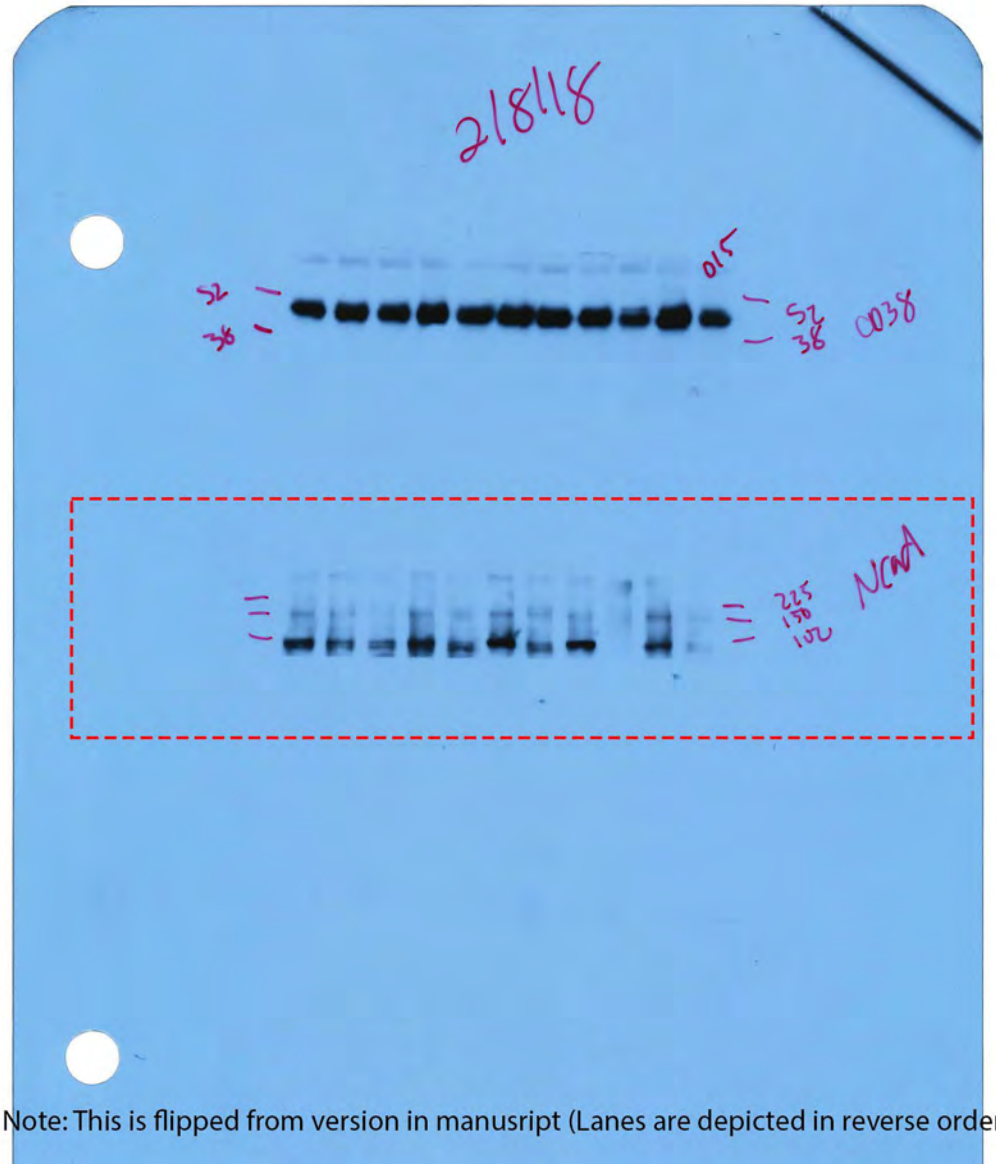

Note: This is flipped from version in manusript (Lanes are depicted in reverse order)

Figure S1C

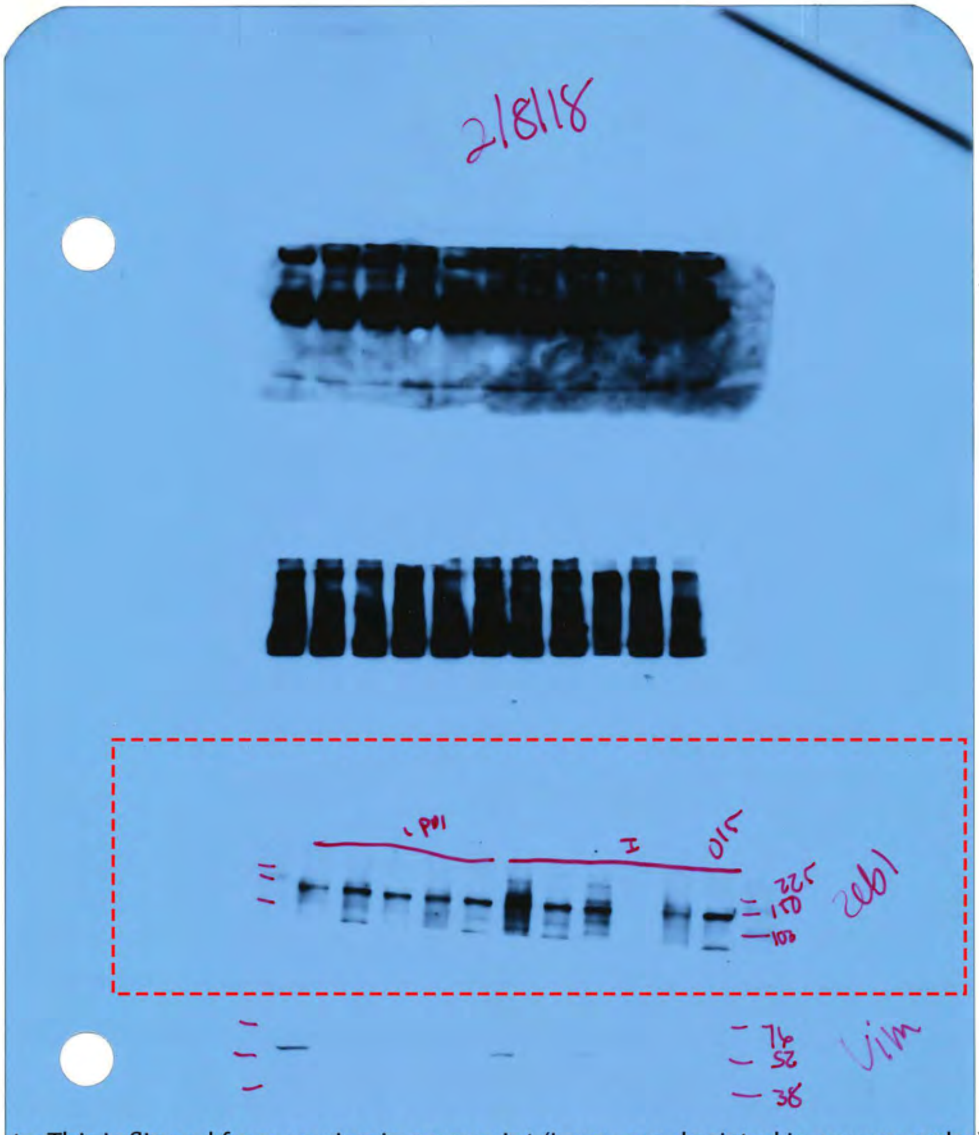

Note: This is flipped from version in manusript (Lanes are depicted in reverse order)

Figure S2B

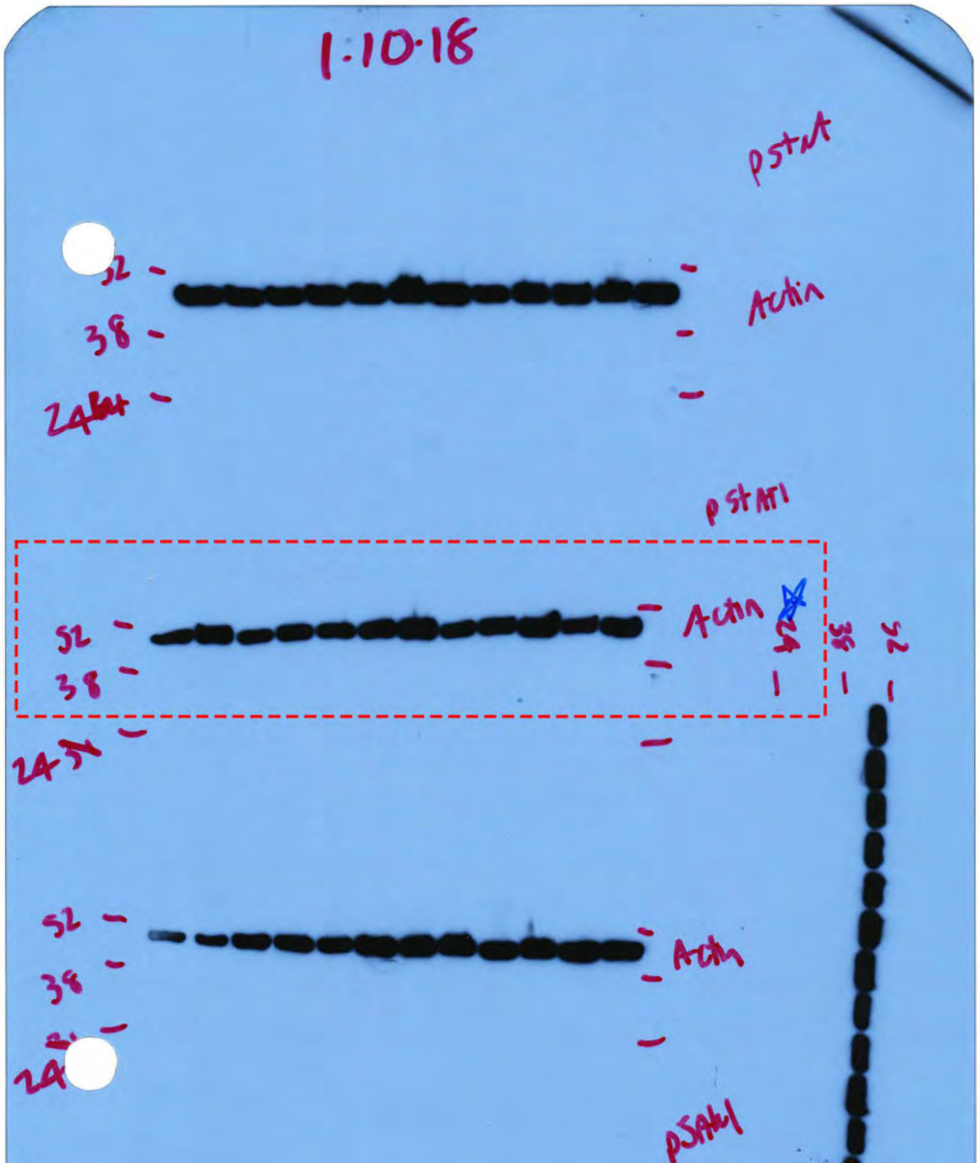

Figure S2B

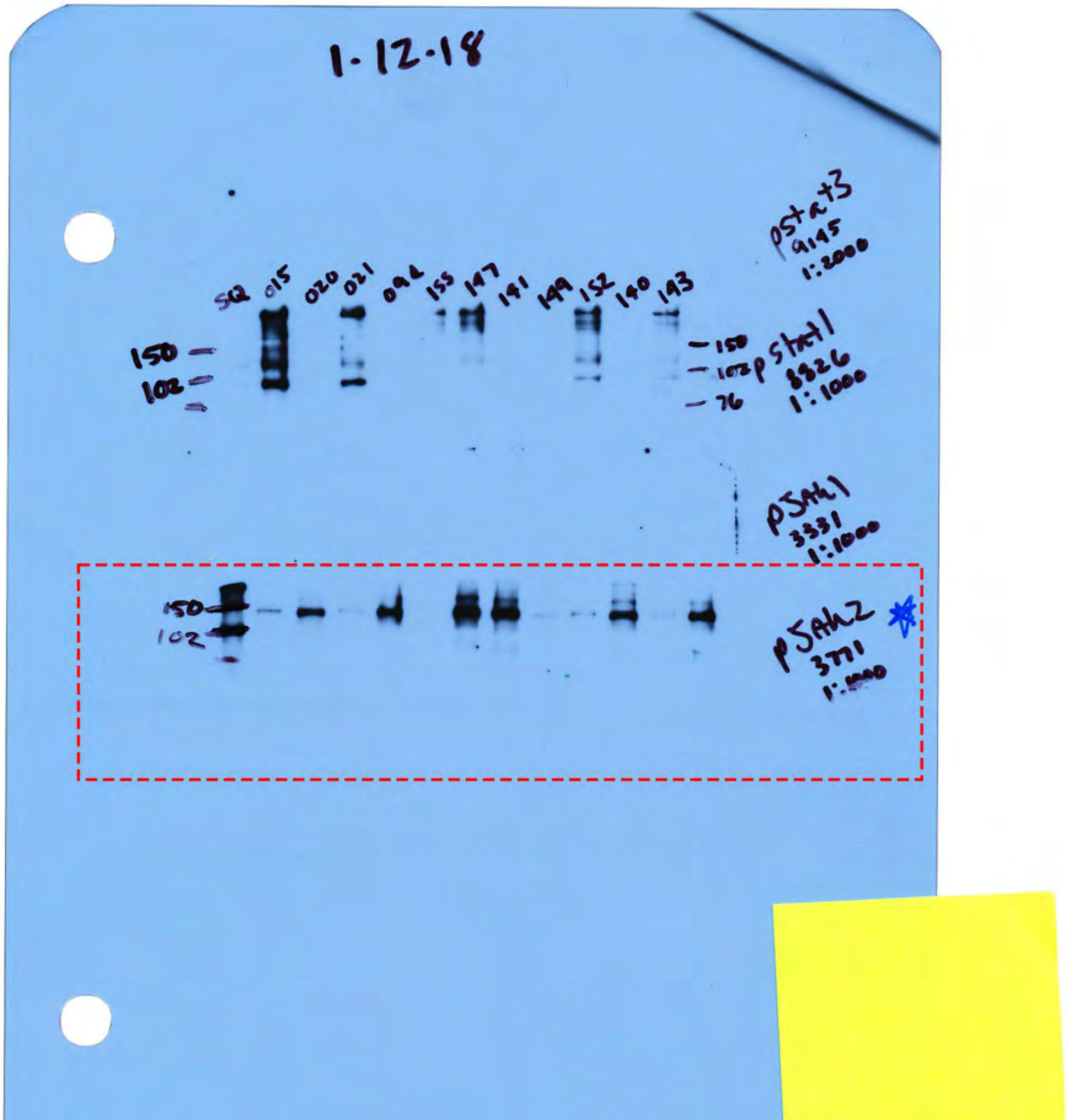
1.12.18
pstat3
9145
1:2000
SQ 015 020 021 092 155 147 141 149 152 140 143
150
102
pstat1
8826
1:1000
pSmad1
3331
1:1000
pSmad2

Figure S2B

Note: blot was placed backwards during developing, so lanes are in reverse order

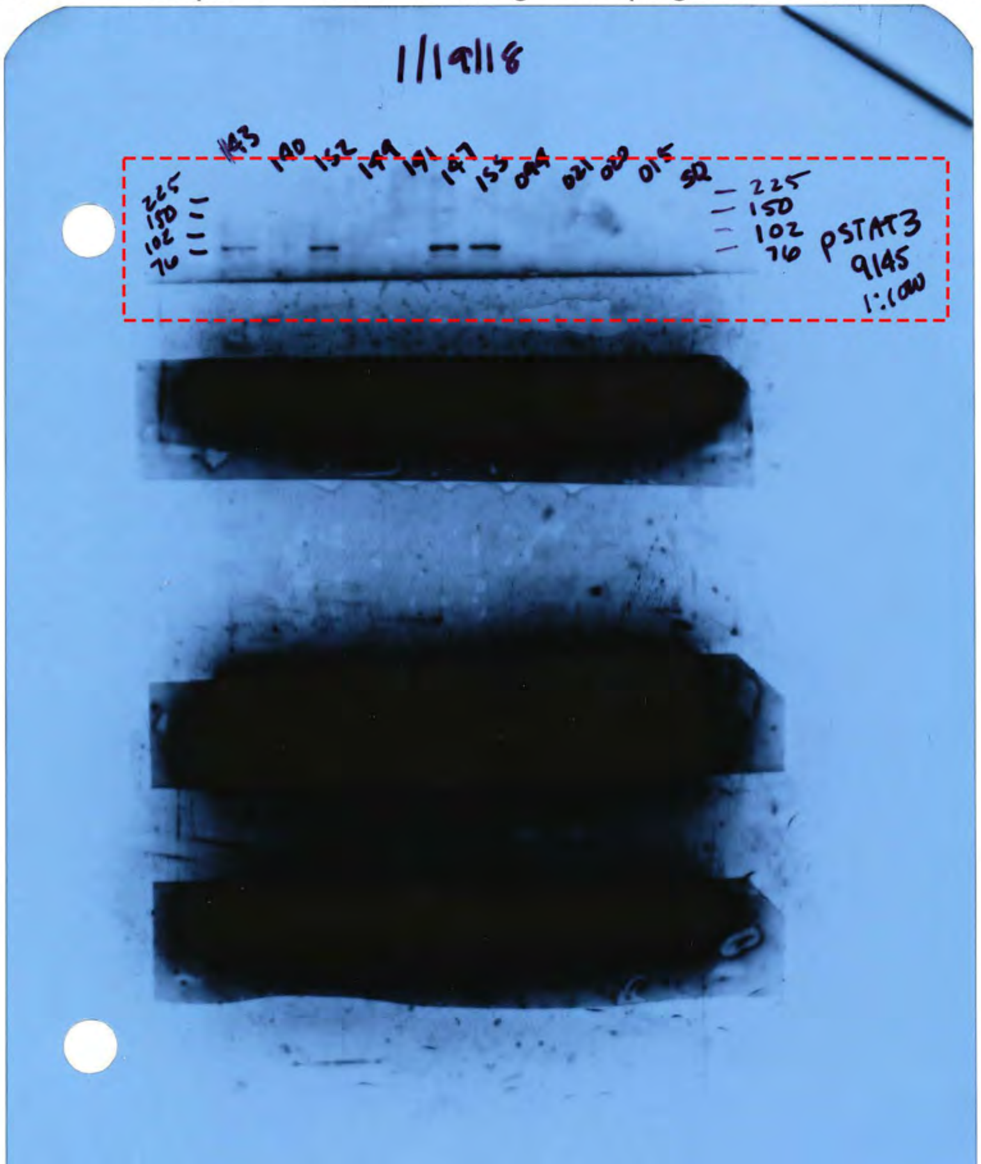

Figure S2B

Note: blot was placed backwards during developing, so lanes are in reverse order

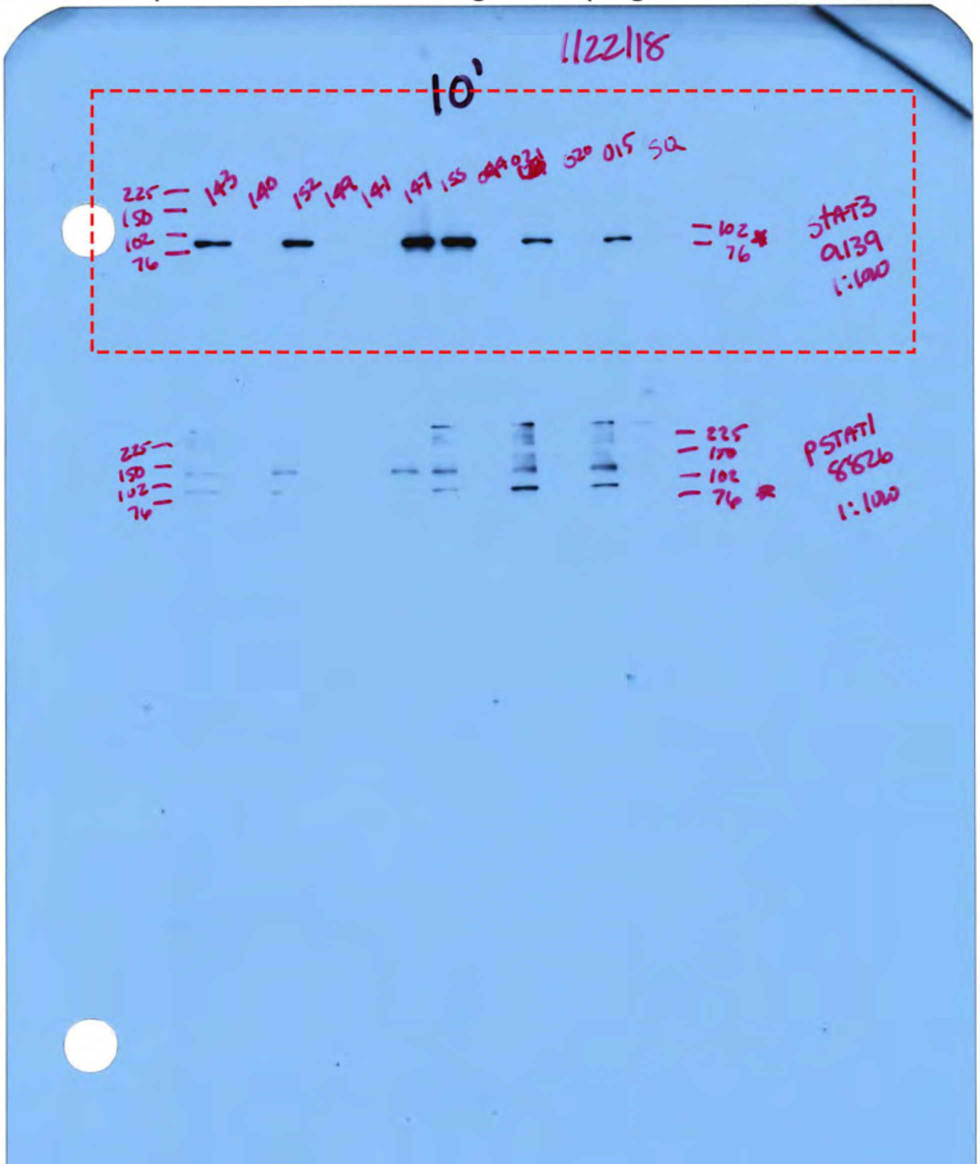

Figure S2B

Note: blot was placed backwards during developing, so lanes are in reverse order

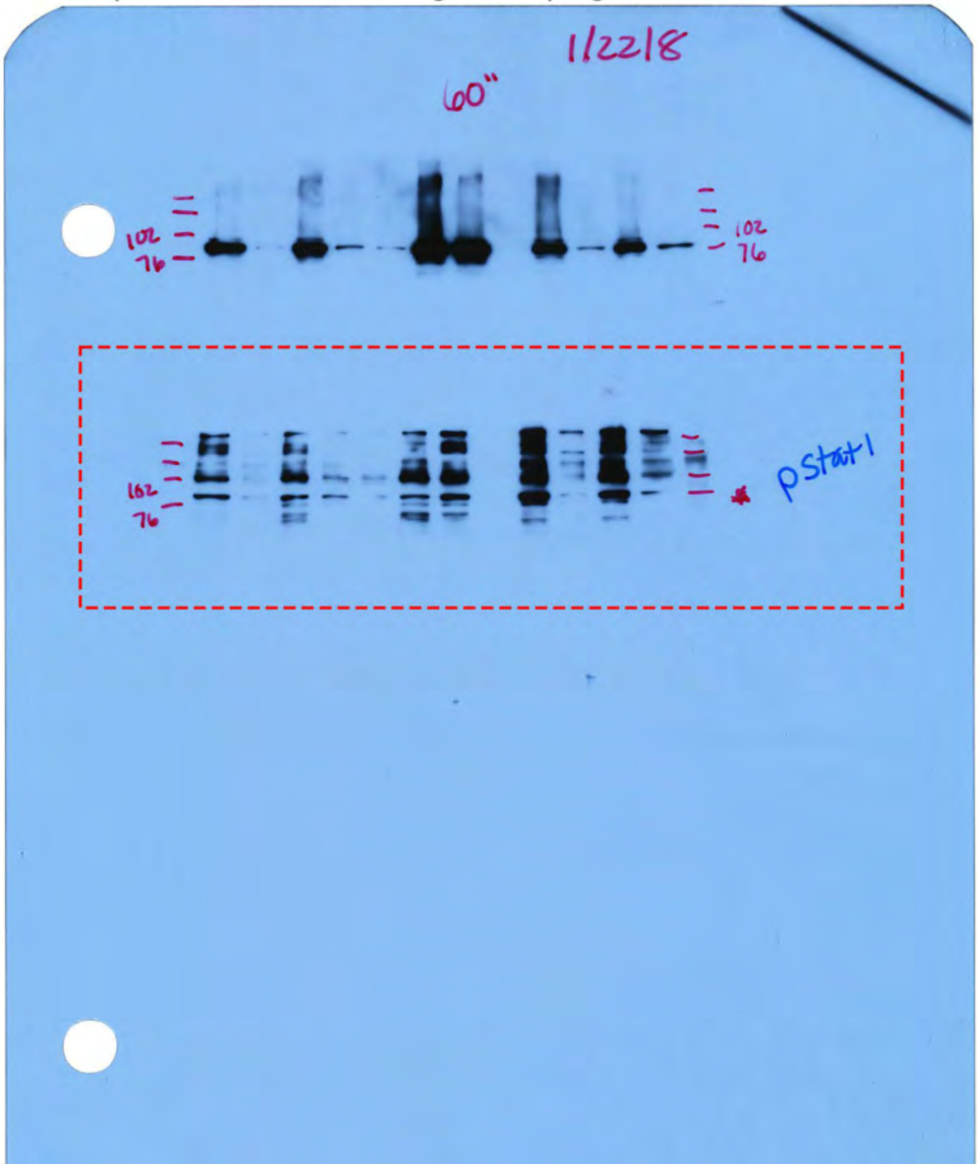

Figure S2B

Note: blot was placed backwards during developing, so lanes are in reverse order

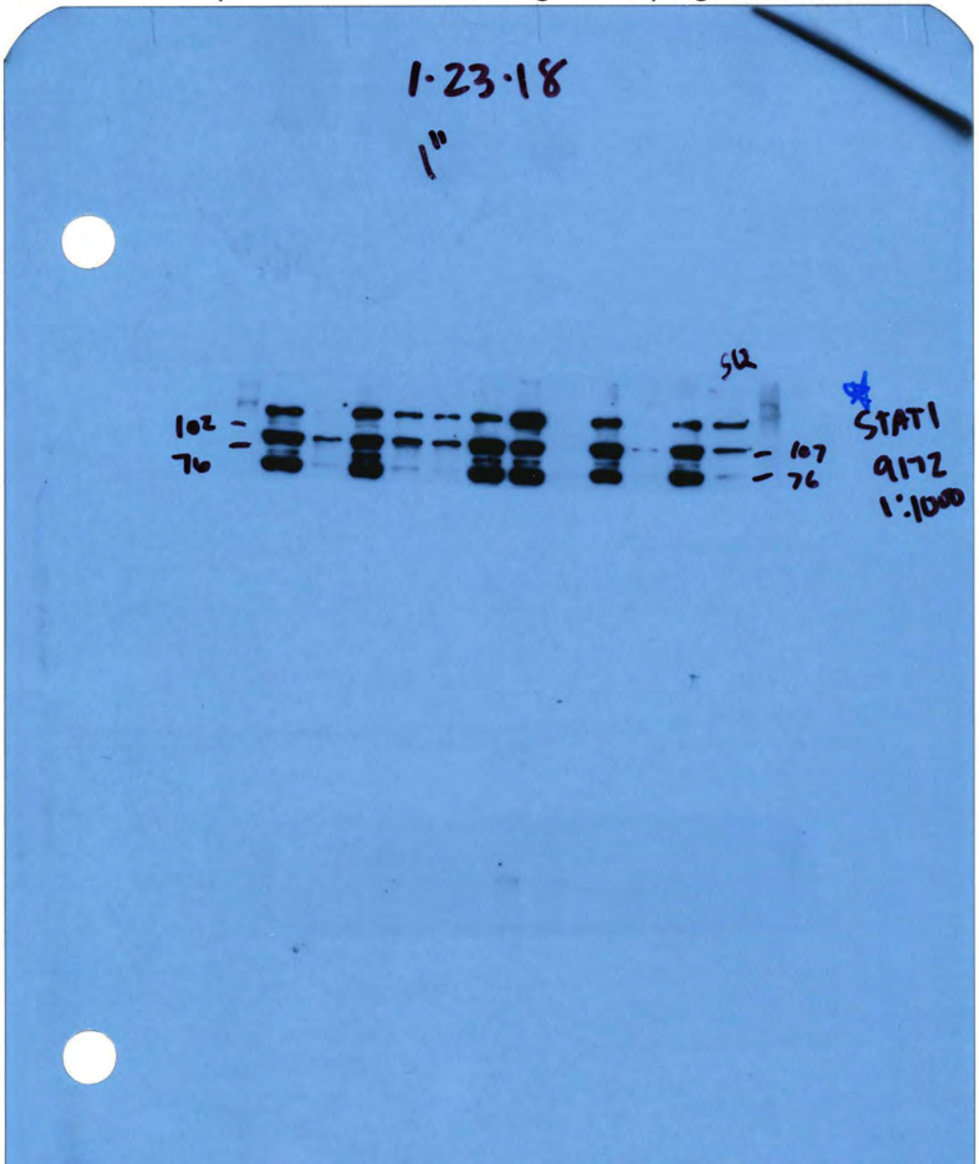

Figure S2B

Note: blot was placed backwards during developing, so lanes are in reverse order

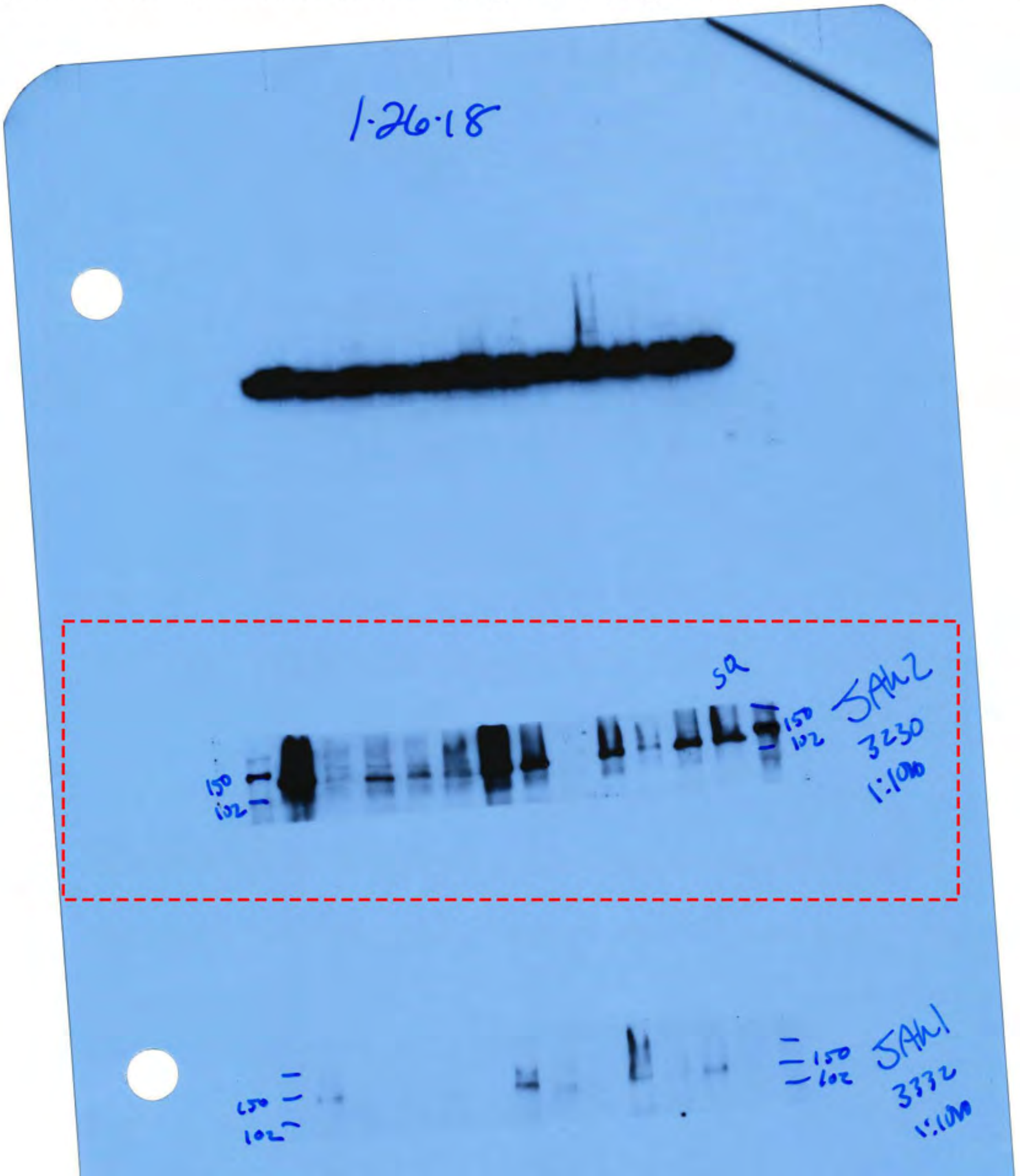

Figure S2G

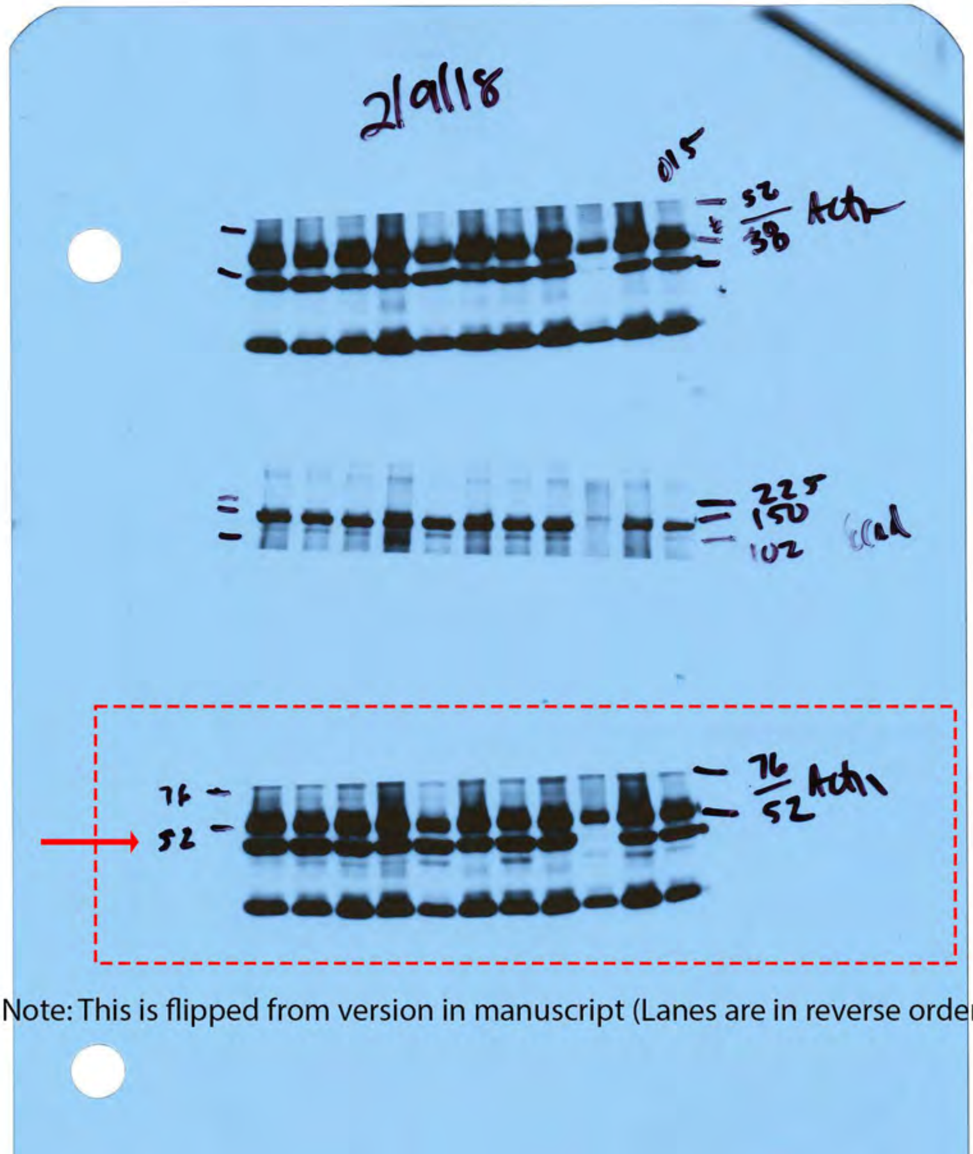

Note: This is flipped from version in manuscript (Lanes are in reverse order)

Figure S2G

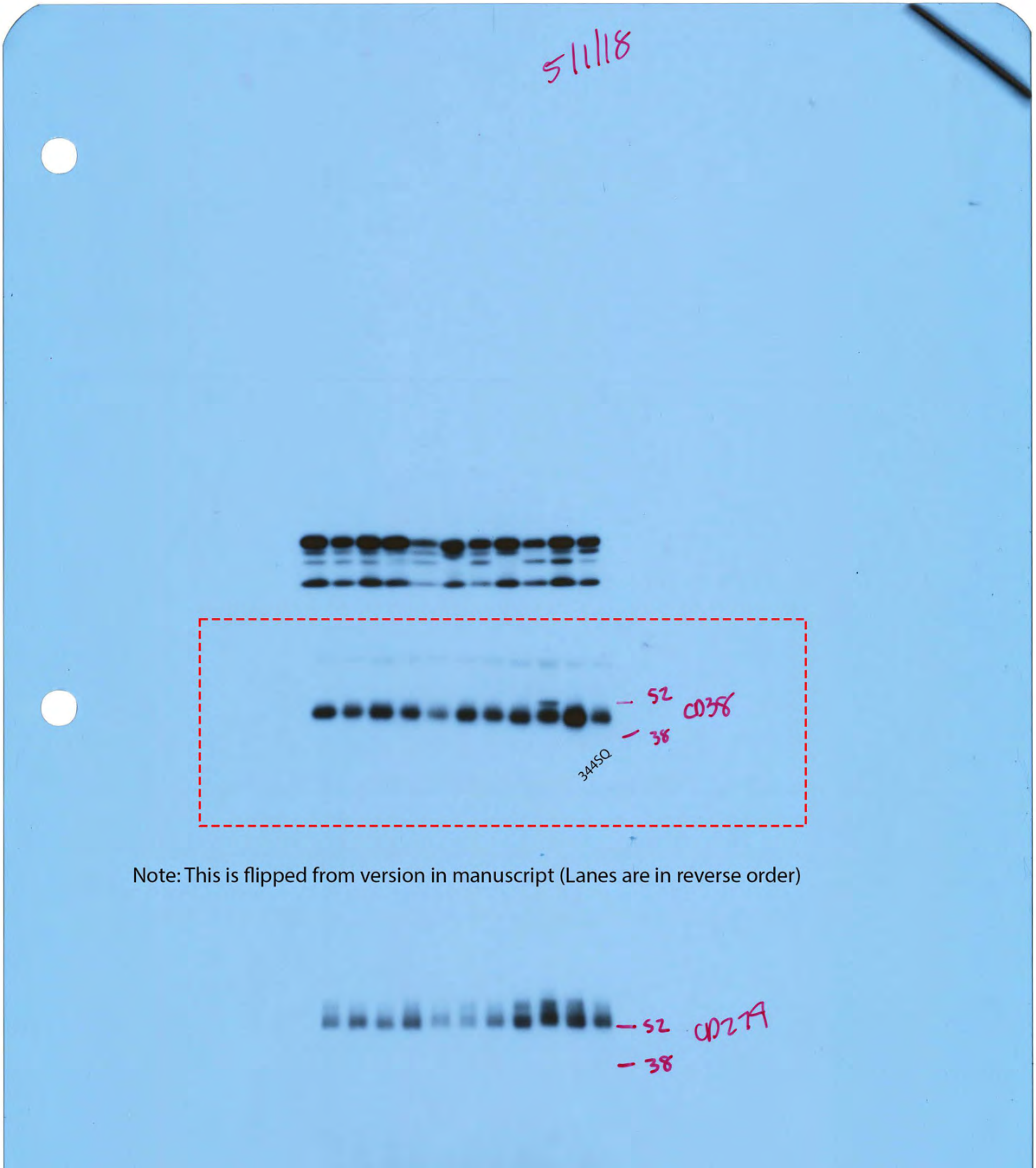

Note: This is flipped from version in manuscript (Lanes are in reverse order)

Figure S2G

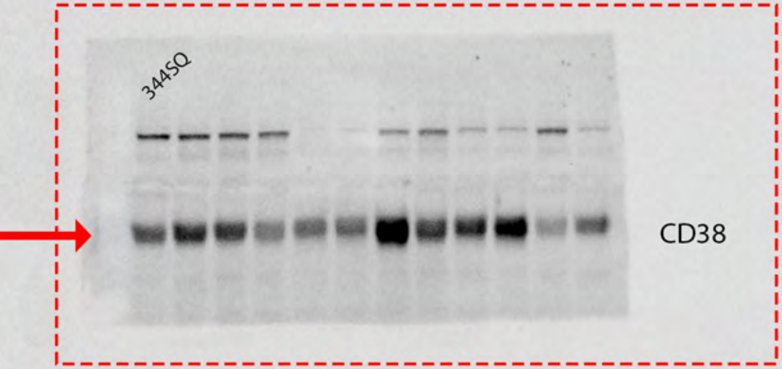

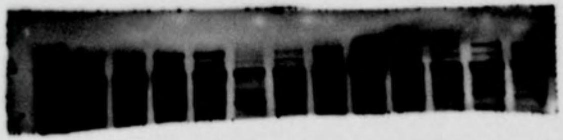

Figure S2G

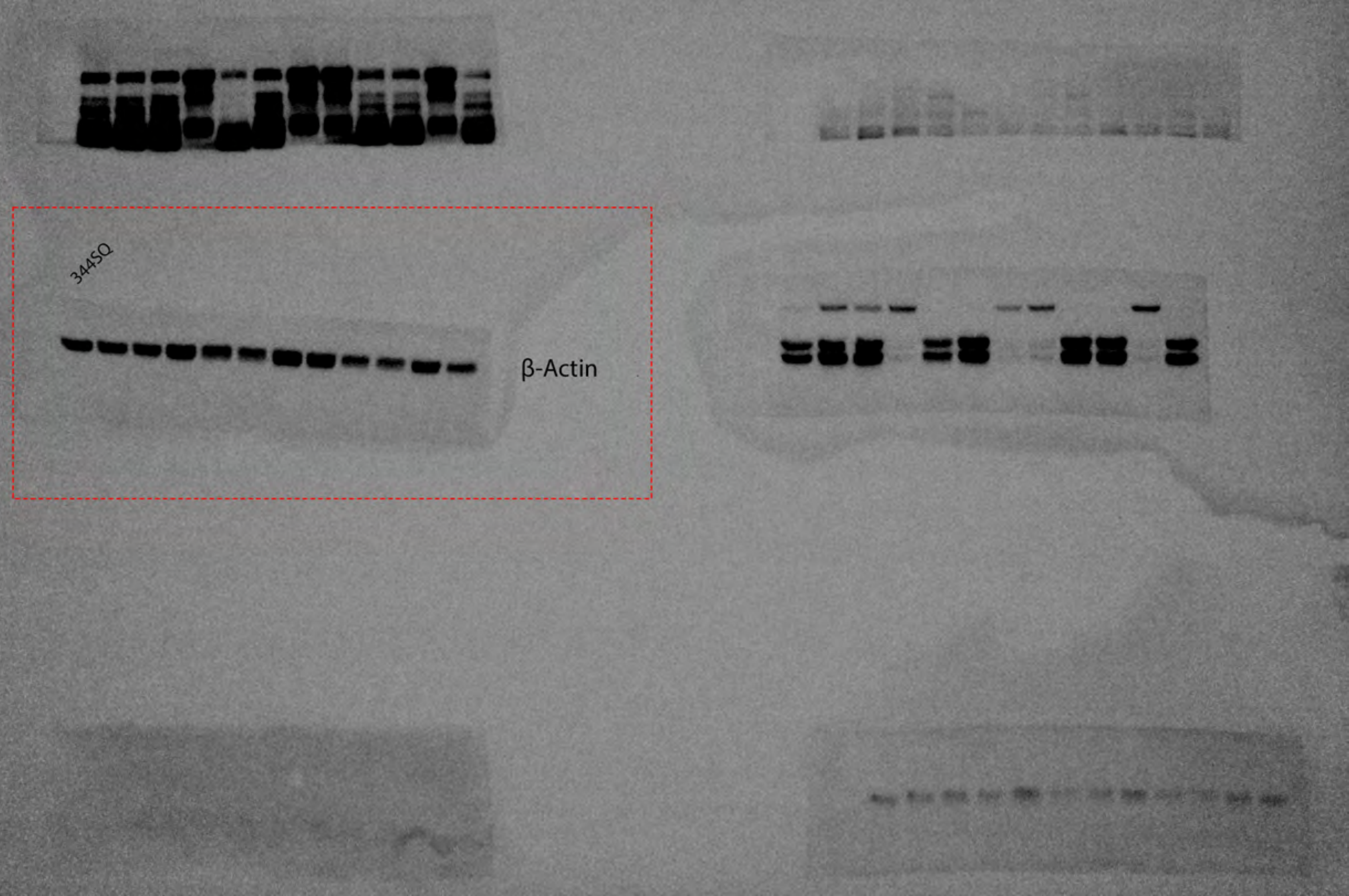

Figure S4A

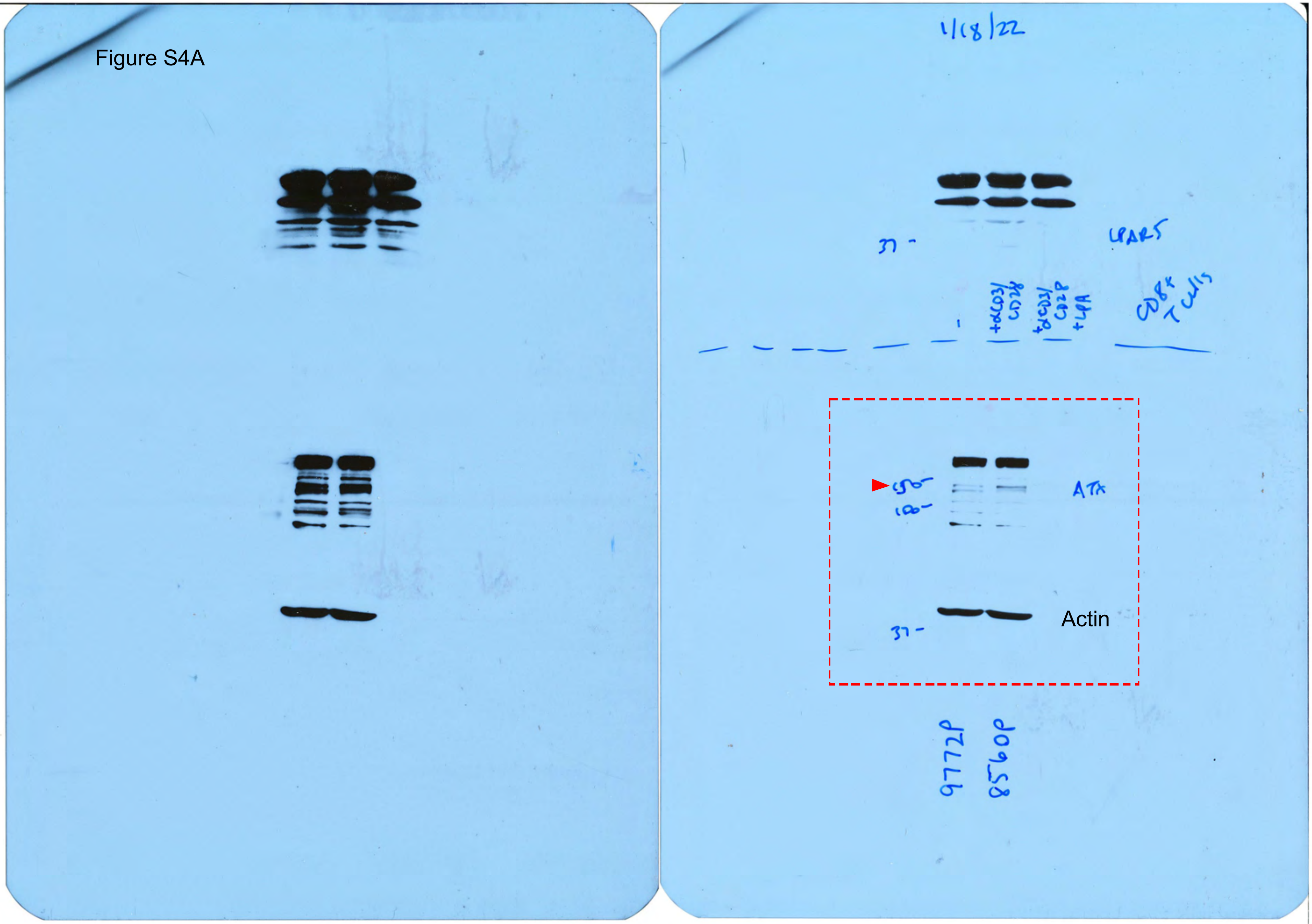

Figure S8E

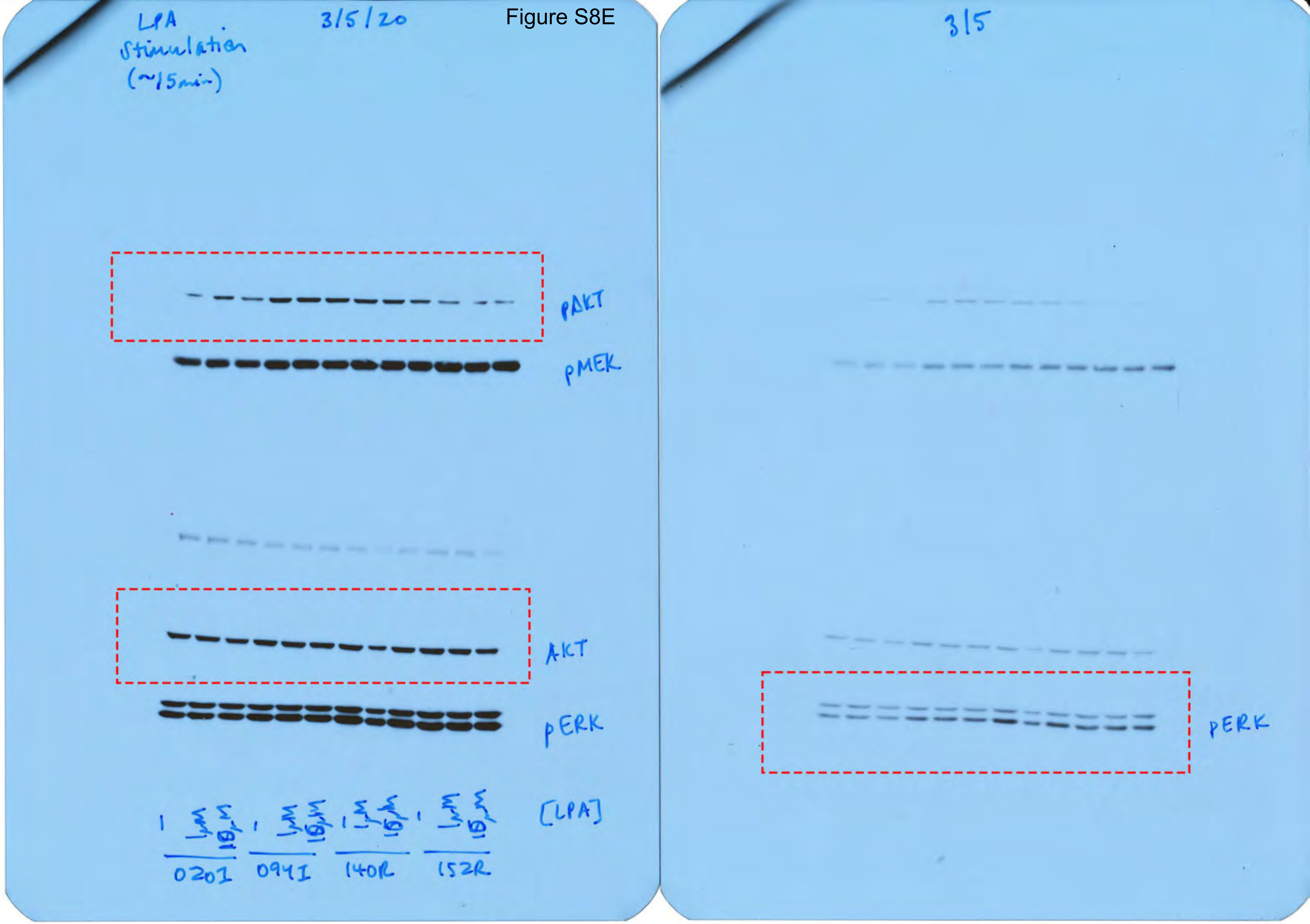
LPA stimulation (~15min)
3/5/20
pAKT
pMEK
AKT
pERK
[LPA]
0201
094I
140R
152R
3/5
pERK

Figure S8E

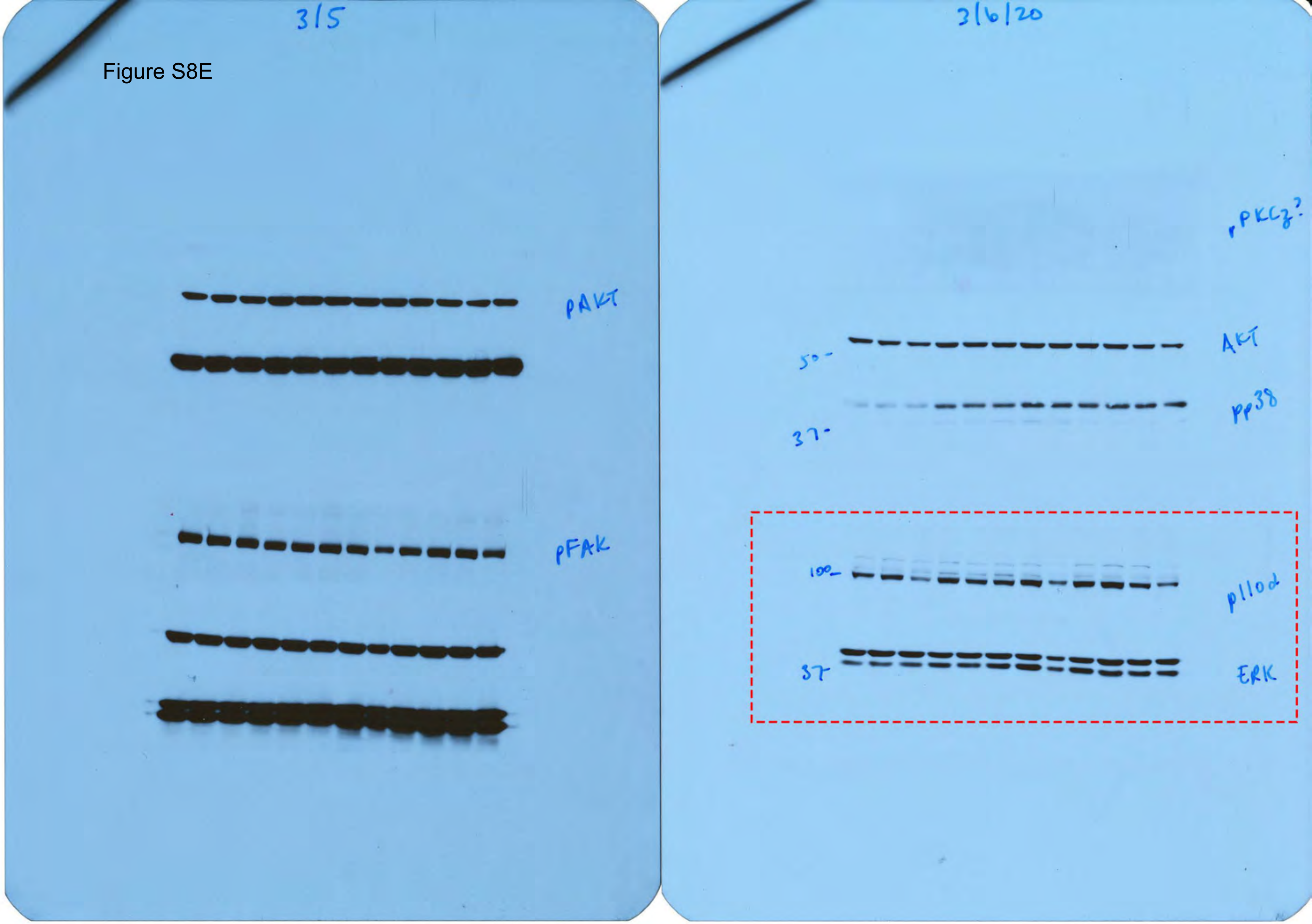

Figure S8E

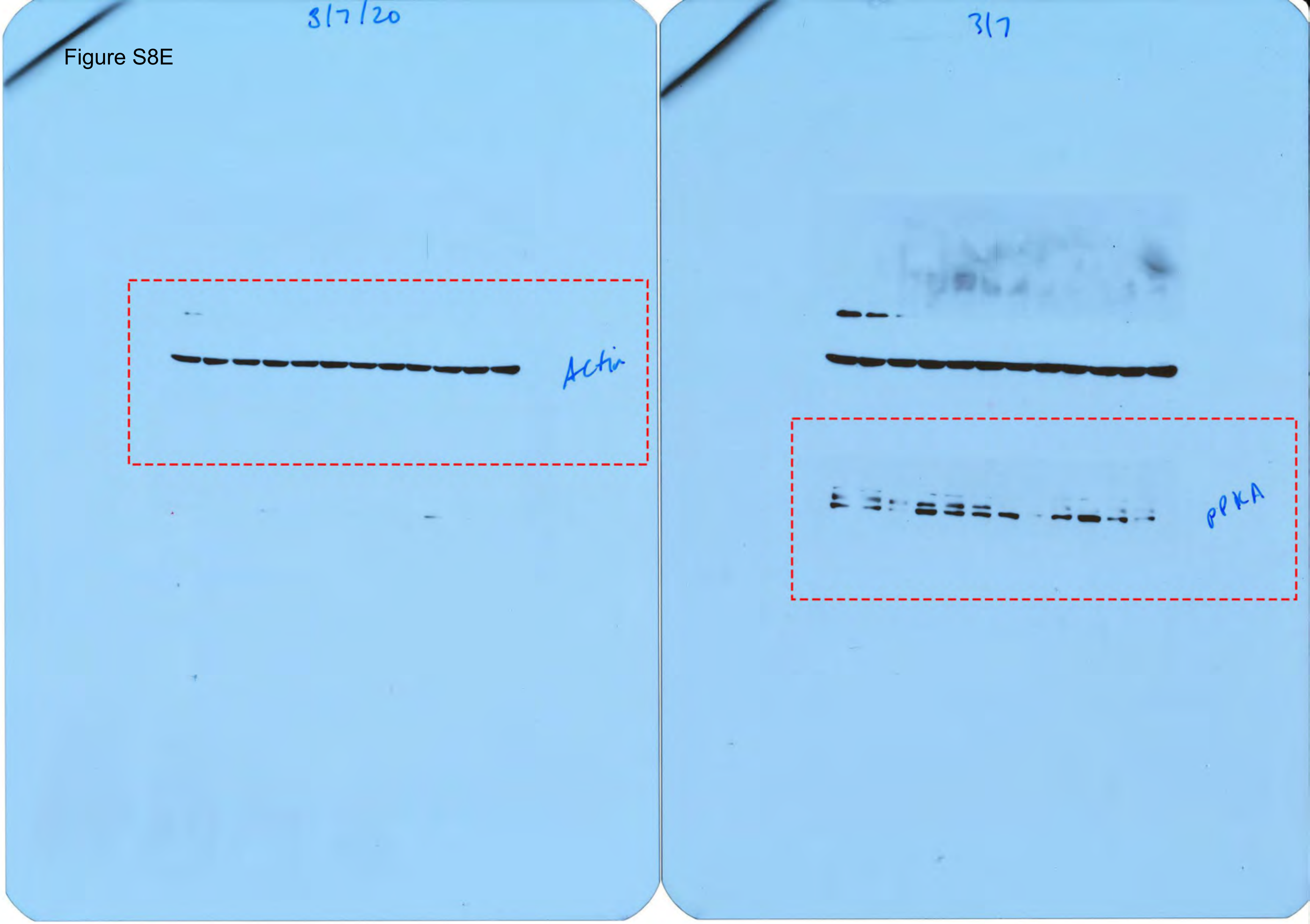

Figure S8E

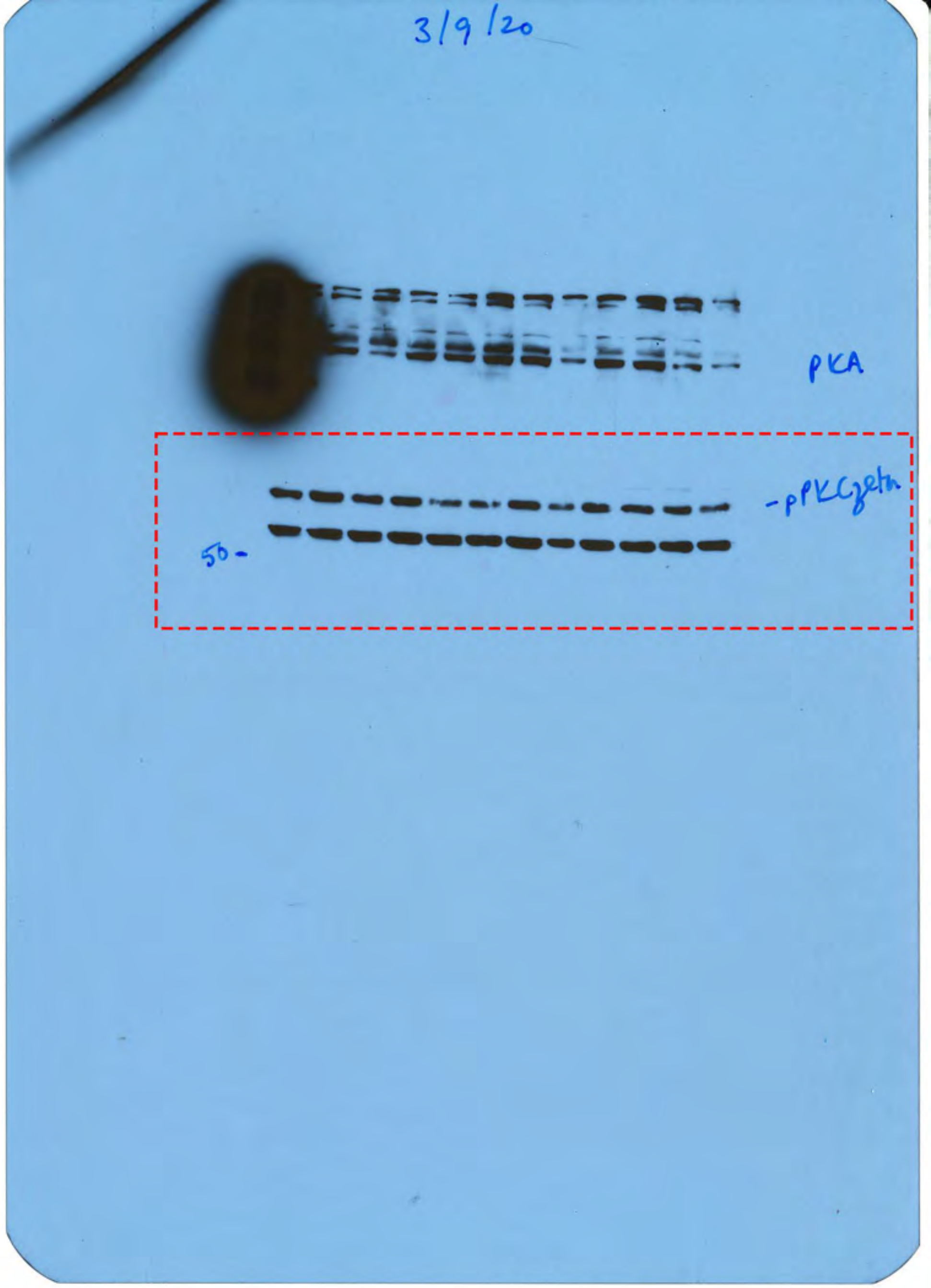

Supplement: Supplemental data [file jci-133-163128-s123.pdf]
